# Risk and protective factors for canine visceral leishmaniasis in the Americas: a systematic review update with meta-analysis

**DOI:** 10.1186/s13071-026-07325-0

**Published:** 2026-03-18

**Authors:** Anna Gabryela Sousa Duarte, Guilherme Loureiro Werneck, Sarah de Farias Lelis, Eduardo Sérgio da Silva, Álisson Oliveira dos Santos, Fábio Raphael Pascoti Bruhn, Tiago Silveira Gontijo, Lucas Edel Donato, David Soeiro Barbosa, Paulo Henrique Araújo Soares, Vinícius Silva Belo

**Affiliations:** 1 Universidade Federal de São João del Rei (UFSJ), Campus Centro-Oeste Dona Lindu, Divinópolis, MG Brazil; 2 Prefeitura Municipal de Divinópolis-Minas Gerais, Secretaria Municipal de Saúde, Divinópolis, MG Brazil; 3 Departamento de Epidemiologia, Universidade do Estado do Rio de Janeiro, Rio de Janeiro, RJ Brazil; 4 Ministério da Saúde do Brasil, Secretaria de Vigilância em Saúde e Ambiente, Brasília, DF Brazil; 5 Universidade Federal de Mato Grosso do Sul (UFMS), Campus de Três Lagoas (CPTL), Três Lagoas, MS Brazil; 6 Departamento de Veterinária Preventiva, Universidade Federal de Pelotas (UFPel), Pelotas, RS Brasil; 7 Departamento de Parasitologia, Universidade Federal de Minas Gerais (UFMG), Belo Horizonte, MG Brazil; 8 Instituto Federal do Norte de Minas Gerais, Campus Salinas, Salinas, MG Brazil

**Keywords:** Protozoan infections, Vector-borne diseases, *Leishmania infantum*, Meta-analysis, Bias, Risk factors, Quality of evidence

## Abstract

**Background:**

Identification of risk and protective factors for canine visceral leishmaniasis (CVL) is essential to understand the epidemiology of the disease and direct prevention and control strategies. Building on a previous systematic review, this study presents new findings relating to the associations between CVL and a range of variables.

**Methods:**

The systematic review included articles from the previous review (up to September 2011) and additional studies published thereafter regarding factors associated with CVL in the American continent. The inclusion criteria encompassed studies that analyzed associations between CVL and socioeconomic, environmental, household-level, or dog-level variables, regardless of the diagnostic method employed. Meta-analyses were conducted using random-effects models and subgroup analyses, while the Grading of Recommendations Assessment, Development and Evaluation (GRADE) approach was used to classify levels of evidence.

**Results:**

Of the 111 studies included in the analysis, the vast majority were cross-sectional (87.4%), conducted in Brazil (95.5%), and employed serological diagnostic tests alone (77.5%). The variables most consistently associated with CVL, based on moderate levels of evidence, were short-haired dogs, dogs dwelling in the peridomicile or with free access to the street, and proximity of the domicile to green areas. Male dogs, large dogs, ectoparasite-infested animals, dogs in contact with horses, and living in homes with yards also had higher odds of infection, although the levels of evidence were low. Other variables associated with CVL but assigned with very low levels of evidence included the presence of chickens, cats, sand flies, and other dogs in the dog environment; previous cases of CVL in the domicile; substandard custodianship; and guardians with poor education and low income. The quality of the studies has improved since the last review, although methodological limitations were still present, in particular, the absence of control for confounders.

**Conclusions:**

The analyses performed in this review strengthen the current knowledge of CVL and highlight the importance of further research to better understand some of the associated variables. Additional cohort and case–control studies are required, particularly those utilizing molecular diagnostics and adequate control for confounding factors. This review represents progress in understanding the determinants of CVL in the Americas and provides support for improving prevention and control strategies.

**Graphical abstract:**

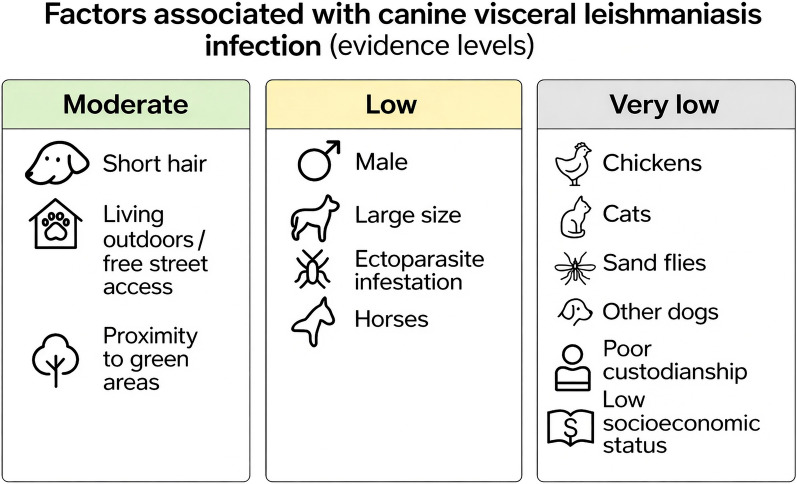

**Supplementary Information:**

The online version contains supplementary material available at 10.1186/s13071-026-07325-0.

## Background

Human and canine visceral leishmaniasis (HVL and CVL, respectively) are neglected diseases that pose serious public health problems in the Americas [1]. These conditions are caused by the protozoan *Leishmania infantum*, transmitted by the bites of female sand flies [2]. Dogs are the main reservoirs of visceral leishmaniasis (VL) in urban environments [3], and humans are the accidental hosts [4].

Considering the importance of dogs in the epidemiology of infections by *L. infantum* [5] and the numerous sociodemographic and environmental aspects involved in transmission, effective control of VL requires an integrated approach encompassing actions across all four pillars of the One Health concept [6, 7]. In this context, identifying determinants of risk and protection in CVL is essential for directing prevention and control actions most appropriately [8, 9] and for allocating financial resources more effectively [10].

A systematic review of the factors associated with CVL in Brazil was published in 2013 [11] and included studies from 1980 to 2011. Although this review addressed several relevant aspects and highlighted important knowledge gaps, numerous studies in the subject area have been published in recent years [8, 12–15]. Noting that conclusions drawn in systematic reviews based on outdated literature may no longer be valid, it is appropriate to update the earlier review by incorporating new evidence and by reassessing associations identified earlier alongside previously underexplored variables. Furthermore, a review upgrade allows new analytical methods to be applied and the evolution of methodological quality to be assessed [16].

In light of the above, this review builds on that published in 2013 [11] by incorporating new studies on CVL-associated factors performed throughout the Americas. In this updated version, new methodologies have been applied to evaluate the levels of evidence of identified associations and to assess the quality of research.

## Methods

### Type of study and inclusion and exclusion criteria

This systematic review with meta-analysis was performed according to the guidelines of the Meta-analysis of Observational Studies in Epidemiology (MOOSE) statement [17] and the 2020 Preferred Reporting Items for Systematic Reviews and Meta-Analyses (PRISMA) [18]. The protocol was registered on the International Prospective Register of Systematic Reviews (PROSPERO) platform under the number CRD42020197056.

The inclusion criteria were: (1) all studies included in the 2013 review [11] whose literature search was completed in September 2011 and (2) any investigation, performed within the American continent and published between October 2011 and June 2024, whose subject met previous conditions, namely a cross-sectional, cohort, or case–control study that analyzed an association between CVL and a socioeconomic, environmental, household-level, or dog-level variable regardless of the diagnostic method employed. To reduce publication and dissemination bias and ensure a comprehensive retrieval of evidence, peer-reviewed articles, doctoral theses, and dissertations were considered eligible. When theses or dissertations resulted in subsequent peer-reviewed publications, only the journal articles were retained to avoid duplicate data.

The exclusion criteria encompassed articles that were entirely descriptive and those with inconsistent or unavailable data, even after attempts to contact the authors directly. Moreover, studies dealing with variables relating to control measures were not included.

### Search and selection of studies and data extraction

Searches were conducted independently in June 2024 by two researchers and included studies published up to and including June 2024. In PubMed, the search strategy combined the terms (“Visceral Leishmaniasis” OR *Leishmania infantum*) AND (“risk factors” OR “associated factors” OR “epidemiological studies” OR immunology OR epidemiology). In LILACS, the terms (“visceral leishmaniasis” OR “leishmaniose visceral”) AND (“risk factors” OR immunology) were used. In the CAPES Databank, searches were performed using “leishmaniose visceral” AND epidemiol*. Google Scholar searches were conducted using the strategy allintitle: “leishmaniose visceral.” These search terms were the same as those used in the 2013 review [11] and were also applied in searches addressing factors associated with HVL [7]. Species-specific terms such as “dog” or “canine” were not included to avoid unnecessarily restricting the retrieval of relevant studies.

The titles and abstracts of articles that matched the inclusion criteria were retrieved and analyzed for relevancy. After removing duplicate articles and those considered irrelevant, the remaining studies were read in full to determine whether they should be included in the review. Any disagreements between the two reviewers were resolved by consensus, with the involvement of a third researcher.

### Assessment of susceptibility to bias

The limitations and susceptibility to bias of studies included in both the 2013 and current reviews were analyzed using Joanna Briggs Institute tools, which classify the quality of publications as good, moderate, or low [19]. The assigned classifications and scores obtained for the two sets of studies were compared using descriptive statistics.

### Meta-analyses

Measures of association between CVL and variables recorded in at least four primary studies in which the same association had been evaluated were combined to provide a more robust summary relationship. A random-effects model was used to calculate the pooled odds ratios (OR) and 95% confidence interval (95% CI).

The *I*^2^ statistic was calculated to quantify the degree of heterogeneity. Then, to explore potential sources of heterogeneity, studies were stratified according to the following subgroups: type of study: (1) cross-sectional, (2) cohort, (3) case–control; control for confounding variables: (1) performed, (2) not performed; diagnostic method: (1) serological, (2) serological or molecular/parasitological, (3) serological or other, (4) molecular/parasitological; and review period: (1) 2013 review, (2) current (present) review. Subgroup analyses were conducted only for variables that had been considered in a sufficient number of studies. Except for the subgroup “control for confounding variables,” the results were presented only for variables with adequate explanatory capacity for heterogeneity, namely, high heterogeneity between subgroups and low heterogeneity within subgroups. The *Q* test was used to assist in this determination. In addition, sensitivity analyses were conducted for the overall pooled estimates of associations supported by moderate levels of evidence by excluding doctoral theses and dissertations, aiming to assess the robustness of the results.

Concerning studies in which more than one diagnostic technique was employed, the results obtained were prioritized for the purposes of meta-analyses in the order: molecular/parasitological tests; molecular/parasitological test combined with serological tests; combined serological tests; enzyme-linked immunosorbent assay (ELISA), dual-path platform (DPP), and immunofluorescence antibody test (IFAT).

Publication bias was analyzed through funnel plots, the Egger test, and Durval and Tweedie’s trim-and-fill method in each meta-analysis that included at least ten studies. The meta-analyses were performed with the aid of Comprehensive Meta-Analysis version 4 software (Biostat, Englewood, New Jersey, USA), while variables that could not be subjected to meta-analysis were summarized using a narrative approach.

### Evaluation of the quality of evidence

The Grading of Recommendations, Assessment, Development and Evaluations (GRADE) approach [20] was employed to classify the associations submitted to meta-analyses according to levels of evidence. Since all studies analyzed were observational, assessment commenced by considering the associations to be of potentially low quality [7, 20–22]. Evaluation continued by examining the criteria capable of reducing the level of evidence (risk of bias, inconsistency, indirect evidence, imprecision, and publication bias). Inconsistency was assessed not only by using statistical measures of heterogeneity but also through a qualitative evaluation of the direction of the associations across studies, as observed in the forest plots. Subsequently, criteria that could increase the level of evidence were considered (magnitude of effect, dose–response gradient, and presence of confounding variables that may have underestimated the observed associations) [20]. For each association, the level of evidence was classified as high, moderate, low, or very low. According to GRADE, high-quality evidence indicates high confidence that the true effect is close to the estimated effect; moderate quality indicates that the true effect is likely close to the estimate, but there is a possibility that it is substantially different; low quality reflects limited confidence in the estimate; and very low quality reflects very limited confidence, with the true effect likely to be substantially different from the estimate [22]. Finally, a GRADE summary of findings table was constructed to summarize the pooled effect estimates, the number of dogs, and the certainty of evidence for the exposures with a moderate level of evidence.

## Results

**Fig. 1:**
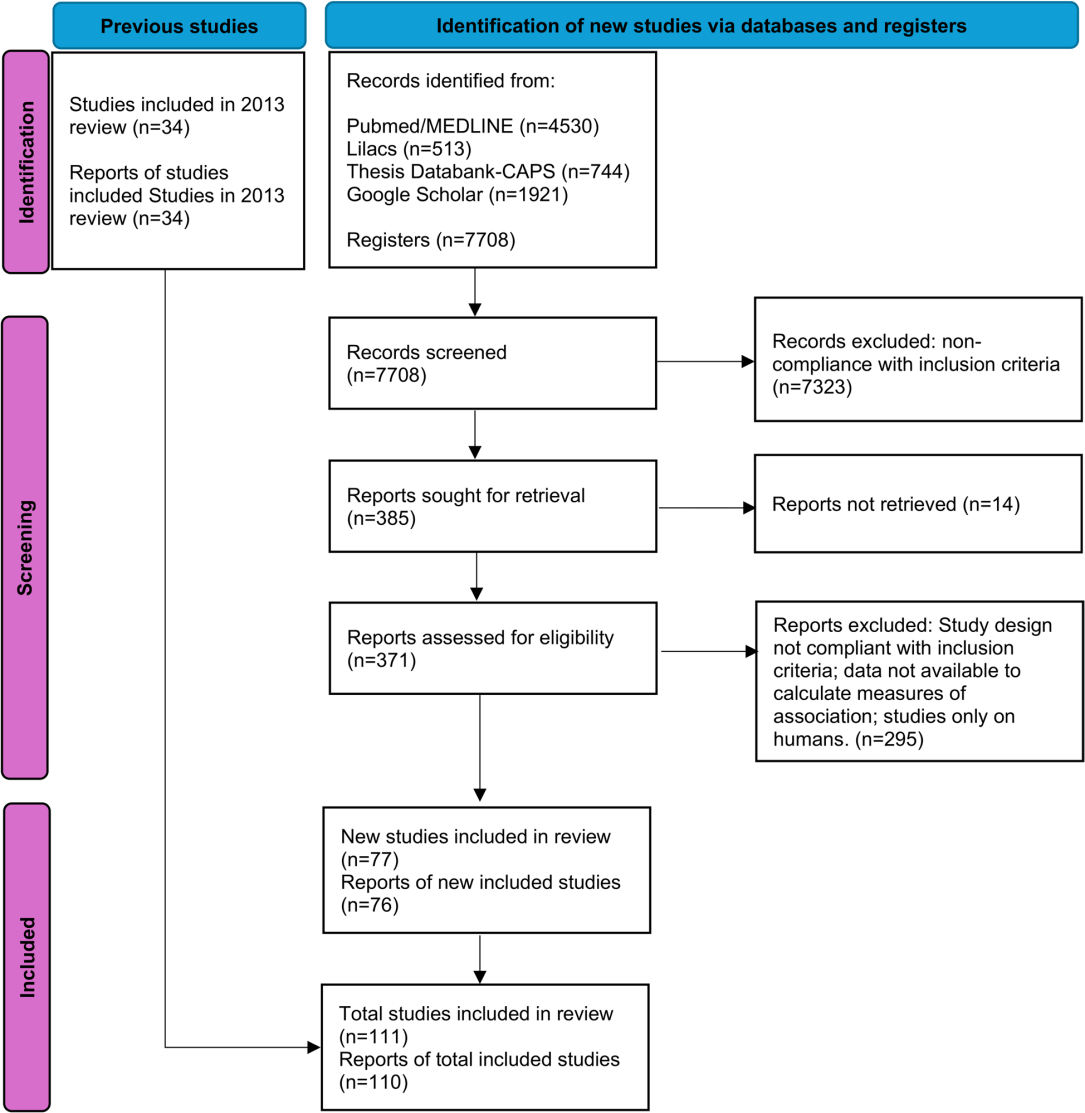
Flow chart summarizing the search and selection process of the reports (individual publications) and studies (unique investigations) included in the review

The 2013 review encompassed 34 primary studies (34 reports) [23–56], while a further 77 primary studies (76 reports) [8, 9, 12–15, 57–126] were incorporated into this review, giving a total of 111 studies (110 reports). The PRISMA flow chart (Fig. 1) summarizes the search and selection process of the studies included.

Regarding the study designs, 97 (87.4%) were cross-sectional, 9 (8.1%) were cohort, and 5 (4.5%) were case–control. Most (106; 95.5%) of the studies were carried out solely in Brazil, although one was performed conjointly in Brazil, Argentina, and Paraguay, while the remainder were conducted in other Latin American countries, namely, two in Colombia, and one each in Mexico and Bolivia. Regarding diagnostic approaches, 77.5% of the studies relied exclusively on serological methods, with ELISA and IFAT being the most commonly used tests, followed by DPP. Detailed information regarding each of the studies included is available in Additional File 1.

### Susceptibility to bias

Regarding the cross-sectional studies, 46 [8, 9, 12, 25, 38, 43, 49, 56, 59, 61–64, 71, 74, 77, 80, 82, 85, 88, 90, 93–100, 104–109, 111, 113–115, 118, 121–125] were classified as high quality; 50 [13–15, 23, 26, 29, 30, 32–34, 36, 37, 39–42, 44–48, 50–54, 57, 58, 60, 67, 70, 73, 76, 78, 79, 81, 83, 86, 87, 89, 92, 101–103, 110, 112, 116, 117, 119, 120] as moderate quality; and only 1 [35] as low quality (Additional File 2). Among the cohort studies, four [27, 68, 69, 91] were classified as high quality, three [31, 72, 126] as moderate quality, and two [24, 28] as low quality. All five of the case–control studies [55, 65, 66, 75, 84] were classified as moderate quality.

Comparison of the quality of studies included in the 2013 review [11] and those incorporated into this review revealed a noticeable improvement over the years. Thus, while two (50%) of the four cohort studies in the 2013 review were classified as low quality and just one (25%) as high quality, in this review, three (60%) of the five newly incorporated cohort studies were categorized as high quality. Furthermore, only 17.2% (5/29) of the cross-sectional studies in the 2013 review were classified as high quality, while the proportion increased to 63.3% (41/68) in the newly incorporated studies. The improvement in methodological quality over time was mainly related to clearer descriptions of study populations and settings; more standardized and valid measurement of exposures and outcomes; and increased use of appropriate multivariable statistical analyses, including better identification and handling of potential confounders.

However, despite the improvement in more recent studies, various limitations were identified, notably the lack of details regarding discontinued participation in the study (losses to follow-up or refusals) and the strategies adopted to deal with such occurrences. In addition, inadequate identification and control of potential confounding variables remained a common limitation.

### Overview of the associations and levels of evidence

The results describing the associations between CVL and the variables of interest are organized into thematic categories. Meta-analysis results with subgroup and publication bias assessments are presented first, followed by meta-analyses that could not be stratified into subgroups. Finally, associations that were not eligible for meta-analysis are described narratively. Variables with similar definitions were incorporated into existing categories, regardless of the analytical approach applied. Subgroup analyses that adequately explained heterogeneity are described in the corresponding sections, whereas the detailed results of the heterogeneity assessments are presented in Additional file 3.

This review covered a significantly greater number of studies than the 2013 review [11], enabling an in-depth examination of both previously analyzed and additional variables, along with the identification of levels of evidence for various associations. However, there was a predominance of heterogeneous associations and, in the majority of cases, subgroup analyses were unable to explain the heterogeneities found. Tables 1, 2, and 3 compare the conclusions from the 2013 review with those from the present review regarding the associations between CVL and the targeted variables, grouped according to dog-related characteristics; environmental characteristics and animal presence; and dog care–related and other variables, and summarize the corresponding levels of evidence.

**Table 1:** Comparison of meta-analysis results from the 2013 review and the current review (studies published up to June 2024) regarding associations between *L. infantum* infection and dog-level characteristics, with corresponding levels of evidence

Variable	Number of articles		Main results		Levels of evidence
	2013 Review (search completed up to September 2011)	Current review (cumulative)	2013 review	Current review	
Dog sex	23	69	Slightly higher odds of infection in males (1.12; 0.99–1.27). Presence of heterogeneity	Association similar to that of 2013 review (1.08; 1.01–1.16) Summary measure significant in the combination of studies without control for confounders and presence of heterogeneity	Low^a^ ⊕ ⊕ ⊝ ⊝
Dog age ≤ 1 versus > 1 years ≤ 2 versus > 2 years	12 6	32 16	The odds of infection were lower among younger dogs in both comparisons (≤ 1: 0.89; 0.67–1.17 and ≤ 2: 0.72; 0.48–1.08). Presence of heterogeneity	The odds of infection were also lower among younger dogs in both comparisons (≤ 1: 0.72; 0.61–0.84 and ≤ 2: 0.82; 0.57–1.18). Inverse association in studies with control for confounders. Presence of heterogeneity	Very low^b^ ⊕ ⊝ ⊝ ⊝
Dog breed: mixed breed versus pure breed	7	38	Mixed-breed dogs showed significantly lower odds of infection (0.75; 0.70–0.81). No heterogeneity was observed	Association in the same direction as the 2013 review, but weaker (0.94; 0.84–1.06) Inverse association observed after confounding control. Presence of heterogeneity	Very low^c^ ⊕ ⊝ ⊝ ⊝
Dog hair length: short versus long	11	36	Higher odds of infection in short-haired dogs (1.39; 1.05–1.85). Presence of heterogeneity	Results similar to those of the 2013 review (1.34; 1.15–1.56). Summary measure stronger in studies with control for confounders. Presence of heterogeneity	Moderate^d^ ⊕ ⊕ ⊕ ⊝
Dog size Dog size (nonspecific) Small versus medium Small versus large	2 – –	– 13 15	No meta-analyses were performed	Small dogs had significantly lower odds of infection compared with large dogs (0.63; 0.48–0.84). Weaker association in the comparison between small versus medium-sized dogs (0.84; 0.62–1.13). Presence of heterogeneity	Low^e^ ⊕ ⊕ ⊝ ⊝
Dog hair color: light versus dark	–	10	No meta-analyses were performed	Association between hair color and infection close to null (0.98; 0.82–1.18). Presence of heterogeneity	Very low^b^ ⊕ ⊝ ⊝ ⊝

Association measures are reported as odds ratios (ORs) with 95% confidence intervals (CI), expressed as OR; lower–upper CI

^a^No downgrades applied

^b^Downgraded two levels because of imprecision and high heterogeneity

^c^Downgraded two levels because of imprecision and publication bias

^d^Upgraded one level on the basis of the magnitude of effect in studies with control for confounding factors

^e^Downgraded one level because of high heterogeneity and upgraded one level on the basis of dose–response gradient

*HVL* human visceral leishmaniasis, *CVL* canine visceral leishmaniasis

**Table 2:** Comparison of meta-analysis results from the 2013 review and the current review (studies published up to June 2024) regarding associations between *L. infantum* infection and environmental characteristics and animal presence, with corresponding levels of evidence

Variable	Number of articles		Main results		Levels of evidence
	2013 Review (search completed up to September 2011)	Current review (cumulative)	2013 review	Current review	
Presence of chickens/chicken coop	7	26	Greater odds of infection in dogs raised in the presence of chickens (1.32; 0.78–2.24). Presence of heterogeneity	Association similar to that of the 2013 review (1.29; 1.04–1.60). Summary measure stronger in studies without control for confounders Presence of heterogeneity	Very low^a^ ⊕ ⊝ ⊝ ⊝
Existence of yard and/or nearby vegetation Vegetation Yard Organic material Unpaved yard	6 3 – –	22 6 8 5	No meta-analyses were performed	Higher odds of infection in dogs raised in the presence of vegetation (1.58; 1.26–1.98) especially in the combination of studies with control for confounders. Existence of yard (2.05; 1.25–3.38), organic material (1.56; 1.09–2.22), and unpaved yards (1.41; 0.92–2.15) associated with infection Presence of heterogeneity in all meta-analyses	Moderate^b^ for existence vegetation ⊕ ⊕ ⊕ ⊝ Low^c^ for presence of yard, organic material, and unpaved yard ⊕ ⊕ ⊝ ⊝
Presence of other dogs in the domicile	2	14	No meta-analyses were performed	Higher odds of infection among dogs that lived with other dogs (1.25; 0.89–1.76). Stronger with confounding control. Inverse associations in case–control and cohort studies. Heterogeneity present in all meta-analyses	Very low^d^ ⊕ ⊝ ⊝ ⊝
Presence of other animals Horses/cows Horses Pigs Pigs/pigsties Cats Small rodents Foxes	1 – 1 – – – 2	– 11 – 11 14 7 –	No meta-analyses were performed	Higher odds of infection in the presence of horses (1.55; CI 1.02–2.34), stronger with confounding control. Higher odds with pigs/pigsties or cats and no association with rodents. Heterogeneity present in all meta-analyses	Low^c^ for horses ⊕ ⊕ ⊝ ⊝ Very low^d^ for other animals ⊕ ⊝ ⊝ ⊝

Association measures are reported as odds ratios (ORs) with 95% confidence intervals (CI), expressed as OR; lower–upper CI

^a^Downgraded one level because of publication bias

^b^Upgraded one level on the basis of the magnitude of effect in studies with control for confounding factors

^c^No downgrades applied

^d^Downgraded two levels because of imprecision and high heterogeneity

*HVL* human visceral leishmaniasis, *CVL* canine visceral leishmaniasis

**Table 3:** Comparison of meta-analysis results from the 2013 review and the current review (studies published up to June 2024) regarding associations between *L. infantum* infection and dog care-related and other variables, with corresponding levels of evidence

Variable	Number of articles		Main results		Levels of evidence
	2013 Review (search completed up to September 2011)	Current review (cumulative)	2013 review	Current review	
Presence of ectoparasites	3	14	No meta-analyses were performed	Infested dogs had slightly higher odds of infection (1.12; 0.87–1.46). Stronger association with confounding control. Presence of heterogeneity	Low^a^ ⊕ ⊕ ⊝ ⊝
Dog access to streets	6	29	Higher odds of infection in dogs with free access (1.47; 0.90–2.38). Presence of heterogeneity	Results similar to those of the 2013 review (1.44; 1.16–1.78). Stronger association with confounding control. Presence of heterogeneity	Moderate^b^ ⊕ ⊕ ⊕ ⊝
Dog’s dwelling area: intradomicile versus peridomicile	4	25	Dogs raised in the intradomicile had lower odds of infection (0.43; 0.22–0.86). Presence of heterogeneity	Results similar to those of the 2013 review (0.51; 0.39–0.67) and consistent across combinations of studies with and without confounding control. Presence of heterogeneity	Moderate^c^ ⊕ ⊕ ⊕ ⊝
Socioeconomic variables and housing conditions of the guardian Schooling Minimum wage ≤ 1 versus > 1 ≤ 2 versus > 2 Quality of water Sewage collection Garbage collection	6 – – – – – –	9 4 5 5 4 9	No meta-analyses were performed	Higher odds among dogs cared for by illiterate guardians (1.21; 0.68–2.15) and by low-income guardians (3.16; 0.93–10.77) No association with water quality; weak associations with sewage and garbage collection Heterogeneity present in all meta-analyses	Very low^d^ ⊕ ⊝ ⊝ ⊝
Other variables submitted to meta-analysis Previous CVL history Guard dog Presence of sand flies Dog sleeping space Dog routine at night Hunting dog Dog adopted from streets Dog castration Formulated food Deworming Regular veterinary care Guardian with knowledge of CVL Previous HVL history	2 3 1 – – – – – – – – – –	8 4 – 11 3 4 4 5 6 3 8 10 5	No meta-analyses were performed	Higher odds of infection with peridomicile sleeping, guard dogs, vector presence, prior CVL/HVL in the domicile, and proximity to infected neighbors Lower odds with regular veterinary care and formulated food No or weak associations for adoption from streets, hunting, owner knowledge of CVL, tethering at night, deworming, and castration Heterogeneity present in several analyses	Very low^d^ ⊕ ⊝ ⊝ ⊝

Association measures are reported as odds ratios (ORs) with 95% confidence intervals (CI), expressed as OR; lower–upper CI

^a^No downgrades applied

^b^Upgraded one level on the basis of the magnitude of effect in studies with control for confounding factors, with no downgrade for publication bias, as the summary measure remained unchanged

^c^Upgraded one level on the basis of the magnitude of effect

^d^Downgraded two levels because of imprecision and high heterogeneity

*HVL* human visceral leishmaniasis, *CVL* canine visceral leishmaniasis

Most of the associations presented evidence classified as very low or low level, mainly because of heterogeneity between study results, methodological limitations, and imprecision of estimates. However, the levels of evidence for some variables, including dog hair length (short versus long), existence of a yard and/or nearby vegetation, access of dog to the streets, and dog living space (intradomicile versus peridomicile), were raised to moderate, indicating greater confidence in the observed measures of association. Additional File 4 presents the GRADE summary of findings table for exposures with a moderate level of evidence.

### Associations submitted to subgroup analysis and assessment of publication bias

#### Dog sex

Overall, 69 studies [9, 14, 15, 23, 25–27, 29, 30, 32, 37–44, 47–49, 53, 54, 56–58, 61, 62, 64, 65, 67–69, 71, 73–80, 83–90, 92, 94, 99–104, 107–110, 114–117, 120, 123, 124] investigated the association between dog sex and CVL. The pooled estimate indicated slightly higher odds of infection in male dogs (OR = 1.08; 95% CI = 1.01–1.16) (Additional File 5: Supplementary Fig. S1). Although the effect size was small, the direction of the association was consistent across the vast majority of studies.

Stratification of studies according to the presence or absence of control for confounders also indicated higher odds of infection among male dogs in both subgroups, but the association was slightly stronger and statistically significant only in studies that did not perform control (OR = 1.09; 95% CI = 1.01–1.18) (Additional File 6: Supplementary Fig. S1). Publication bias was not identified (Additional File 7: Supplementary Fig. S1).

#### Dog age

The association between dog age and CVL was investigated in 48 studies. Using the cut-offs adopted in the 2013 review, 32 compared dogs aged ≤ 1 year with those > 1 year [26, 27, 30, 32, 34, 40, 42, 43, 47, 48, 53, 54, 57, 61, 67, 76, 82, 89, 90, 92, 99, 101, 103–105, 108, 110, 113, 118, 120, 124, 125], while 16 compared dogs aged ≤ 2 years with those of > 2 years [9, 36, 37, 39, 41, 49, 56, 62, 63, 67, 79, 89, 102, 105, 115, 117]. In both comparisons, younger dogs tended to have lower odds of infection, although statistical significance was observed only for the ≤ 1 versus > 1 year group (OR = 0.72; 95% CI = 0.61–0.84) (Additional File 5: Supplementary Fig. S2). In contrast, studies that performed control for confounders suggested an inverse direction for the association (OR = 1.20; 95% CI = 0.73–1.96) (Additional File 6: Supplementary Fig. S2). Publication bias was not identified in any of the analyses (Additional File 7: Supplementary Fig. S3).

#### Dog breed

The association between dog breed and CVL was investigated in 38 studies [9, 13, 26, 27, 42, 43, 49, 51, 53, 62, 63, 65, 71, 74, 76, 78, 80, 83, 84, 86, 88–90, 92, 94, 100–104, 107, 108, 111, 116–119, 123]. Overall, mixed-breed dogs showed slightly lower odds of infection, but the association was weak and not statistically significant (OR = 0.94; 95% CI = 0.84–1.06) (Additional File 5: Supplementary Fig. S3). In contrast, studies that performed control for confounders showed an inverse association (OR = 1.19; 95% CI = 0.78–1.83) (Additional File 6: Supplementary Fig. S3).

Following publication-bias assessment, six studies were imputed, which slightly attenuated the pooled estimate, and the Egger test was significant, supporting the possibility of publication bias (Additional File 7: Supplementary Fig. S3).

#### Dog hair length

A total of 36 studies investigated the association between length of dog hair and CVL [26, 27, 34, 37, 38, 41–43, 48, 49, 56, 58, 63, 68, 69, 74–76, 78, 80, 82, 84, 86, 87, 92, 98, 99, 102, 103, 110, 114, 116, 123–126]. Short-haired dogs presented significantly higher odds of CVL compared with long-haired dogs (OR = 1.34; 95% CI = 1.15–1.56) (Additional File 5: Supplementary Fig. S4), and this estimate was essentially unchanged after excluding doctoral theses and dissertations (OR = 1.36; 95% CI = 1.15–1.62).

In subgroup analyses, the association was stronger and statistically significant in studies that controlled for confounders (OR = 1.44; 95% CI = 1.24–1.68) than in those that did not (OR = 1.25; 95% CI = 0.96–1.63) (Additional File 6: Supplementary Fig. S4). Publication bias was not identified (Additional File 7: Supplementary Fig. S4).

#### Presence of chickens/chicken coop in the domicile

The association between CVL and the presence of chickens or a chicken coop in the dog environment was investigated by 26 studies [9, 12, 27, 38, 40, 43, 52, 66, 78, 80–82, 84, 85, 87, 88, 98, 103, 104, 106, 112, 113, 115, 118, 123, 126]. In general, significantly higher odds of infection were identified in dwellings with chickens or a chicken coop (OR = 1.29; 95% CI = 1.04–1.60) (Additional File 5: Supplementary Fig. S5). The direction of the association was similar regardless of confounder control, but statistical significance was observed only among unadjusted studies (OR = 1.31; 95% CI = 1.00–1.73) (Additional File 6: Supplementary Fig. S5). Possible publication bias was detected, and the imputation of five studies resulted in a reduced strength of the association measure and a loss of statistical significance (Additional File 7: Supplementary Fig. S5).

#### Presence of a yard or vegetation near the domicile or in the adjacent environment

Overall, six studies [69, 80, 98, 99, 106, 114] evaluated the presence of a yard and showed a strong association with CVL (OR = 2.05; 95% CI = 1.25–3.38) (Additional File 5: Supplementary Fig. S7). Because of the limited number of studies, subgroup analyses by confounder control were not performed. Related yard characteristics were also examined. The odds of infection increased in the presence of organic material [69, 80, 87, 91, 102, 103, 113, 114] (OR = 1.56; 95% CI = 1.09–2.22) and tended to be higher when yard paving was absent [34, 91, 99, 106, 126] (OR = 1.41; 95% CI = 0.92–2.15) (Additional File 5: Supplementary Figs. S8, S9). By contrast, adjacent vacant lots [43, 46, 84, 102, 103] showed no clear association (OR = 0.98; 95% CI = 0.75–1.27) (Additional File 5: Supplementary Fig. S10).

A total of 22 studies [42, 43, 49, 62, 63, 68, 78, 80, 81, 84, 88, 91, 95, 98, 99, 102, 106, 111–114, 123] investigated the association between the existence of vegetation near the domicile and CVL, with the pooled analysis indicating higher odds of infection in the presence of vegetation (OR = 1.58; 95% CI = 1.26–1.98) (Additional File 5: Supplementary Fig. S6). This association remained similar in the sensitivity analysis excluding doctoral theses and dissertations (OR = 1.56; 95% CI = 1.22–2.00). The pooled estimate was stronger in adjusted analyses (OR = 1.70; 95% CI = 1.06–2.72) (Additional File 6: Supplementary Fig. S6). Even though the Egger test for publication bias was significant (*P* < 0.05), and one study was imputed, the measure of association remained strong (Additional File 7: Supplementary Fig. S6).

A few studies [84, 85, 96, 99, 102, 106, 113] evaluated specific forms of vegetation (ornamental plants, fruit trees, crops, normalized difference vegetation index [NDVI]), but the number of studies was insufficient to allow pooling or to identify consistent patterns.

#### Presence of ectoparasites

The association between the presence of ectoparasites (ticks and fleas) and CVL was investigated in 14 studies [12, 50, 75, 78, 86, 94, 95, 104, 108, 110, 115, 118, 123, 126]. Overall, ectoparasites were associated with slightly higher odds of CVL, but the pooled estimate was not statistically significant (OR = 1.12; 95% CI = 0.87–1.46) (Additional File 5: Supplementary Fig. S11). In contrast, among studies that controlled for confounders, the association was stronger and statistically significant (OR = 1.64; 95% CI = 1.27–2.12) (Additional File 6: Supplementary Fig. S7). None of the studies were imputed owing to publication bias, even though the Egger test was statistically significant (Additional File 7: Supplementary Fig. S7).

#### Dog access to streets

Meta-analysis of 29 studies [15, 25, 27, 31, 43, 49, 52, 61–63, 68, 75, 76, 78, 80, 81, 84, 88–90, 94, 98, 102–104, 108, 113, 115, 123] showed that free-roaming dogs had higher odds of CVL (OR = 1.44; 95% CI = 1.16–1.78) (Additional File 5: Supplementary Fig. S12), with a similar estimate after excluding theses and dissertations (OR = 1.69; 95% CI = 1.35–2.11). The direction of the association was consistent in adjusted and unadjusted studies, but the pooled estimate was stronger in studies that controlled for confounding (OR = 1.76; 95% CI = 1.14–2.70) (Additional File 6: Supplementary Fig. S8). After imputing six studies for possible publication bias, the pooled estimate attenuated and lost statistical significance, while maintaining the same direction (Additional File 7: Supplementary Fig. S8).

#### Dog’s dwelling area

In total, 25 studies investigated the association between the dwelling area of the dog (intradomicile versus peridomicile) and CVL [13–15, 29, 34, 49, 56, 57, 62, 70, 76, 82, 84, 89, 90, 94, 98, 102, 104, 115, 117, 118, 121, 123, 126]. Dogs maintained indoors had lower odds of infection, with a statistically significant association (OR = 0.51; 95% CI = 0.39–0.67) (Additional File 5: Supplementary Fig. S13). The same pattern was observed when doctoral theses and dissertations were excluded (OR = 0.43; 95% CI = 0.31–0.60) and in analyses stratified by control for confounders (Additional File 6: Supplementary Fig. S9). No studies were imputed because of publication bias (Additional File 7: Supplementary Fig. S9). Meta-analysis of a further four studies [68, 81, 84, 106] revealed that dogs maintained most of the time in the yard had higher odds of CVL (OR = 1.36; 95% CI = 0.77–2.40) (Additional File 5: Supplementary Fig. S14).

#### Presence of other dogs in the domicile

A total of 14 studies investigated the association between the presence of other dogs in the household and CVL [9, 29, 43, 80, 84, 85, 90, 91, 94, 104, 112, 115, 118, 126]. Overall, cohabitation with other dogs was associated with higher odds, but the pooled estimate was not statistically significant (OR = 1.25; 95% CI = 0.89–1.76) (Additional File 5: Supplementary Fig. S15). After stratification, the association was stronger and statistically significant in studies that controlled for confounders (OR = 1.71; 95% CI = 1.11–2.65) (Additional File 6: Supplementary Fig. S11). In subgroup analysis by study type, heterogeneity was better explained, and the direction reversed in case–control (OR = 0.08; 95% CI = 0.02–0.27) and cohort studies (OR = 0.87; 95% CI = 0.36–2.13) studies (Additional File 6: Supplementary Fig. S10).

#### Presence of other animals in the dog environment

Overall, 11 studies [34, 78, 84, 90, 94, 104, 106, 108, 113, 115, 118] examined horses in the dog environment, showing higher odds of CVL (OR = 1.55; 95% CI = 1.02–2.34) (Additional File 5: Supplementary Fig. S16). The association was markedly stronger among studies that controlled for confounders (OR = 3.08; 95% CI = 1.77–5.34) (Additional File 6: Supplementary Fig. S12).

Seven studies [84, 85, 90, 104, 106, 108, 118] on contact with small rodents found no clear association (OR = 0.98; 95% CI = 0.66–1.47) (Additional File 5: Supplementary Fig. S17). Meta-analyses suggested higher—but nonsignificant—odds of CVL in the presence of cats [9, 12, 43, 66, 84, 90, 102, 104, 108, 112, 113, 115, 118, 123] (OR = 1.28; 95% CI = 0.95–1.71) and pigs/pigsties [31, 78, 80, 84, 87, 90, 94, 104, 106, 113, 118] (OR = 1.53; 95% CI = 0.86–2.71) (Additional File 5: Supplementary Figs. S18, S19). One study [76] indicated that the association between the presence of farm animals and CVL was weak and not statistically significant.

### Associations that could not be submitted to subgroup analyses

#### Dog size

The association between dog size and CVL [14, 42, 56, 69, 71, 76, 81, 84, 87, 88, 95, 100, 102, 110, 123, 124] was weaker and not statistically significant in the comparison between small and medium dogs (OR = 0.84; 95% CI = 0.62–1.13), whereas a significant association was observed when comparing small versus large dogs (OR = 0.63; 95% CI = 0.48–0.84) (Additional File 5: Supplementary Figs. S20, S21).

#### Dog hair color

Ten studies [76, 84, 92, 100, 102, 110, 111, 114, 124, 126] examined the association between coat color (light versus dark) and CVL. The summary association measure was close to null and was not statistically significant (OR = 0.98; 95% CI = 0.82–1.18) (Additional File 5: Supplementary Fig. S22).

#### Socioeconomic variables and housing conditions of the guardian

Across nine studies [56, 81, 84, 89, 90, 94, 104, 115, 123], illiteracy among guardians was associated with higher odds of CVL, although the pooled estimate was not statistically significant (OR = 1.21; 95% CI = 0.68–2.15) (Additional File 8: Supplementary Fig. S1).

The summary measure from four studies [14, 77, 80, 108] showed that dogs cared for by guardians with an income ≤ 1 minimum wage had higher odds of infection, with a strong association (OR = 3.16; 95% CI = 0.93–10.77) (Additional File 8: Supplementary Fig. S2). In contrast, the direction of the association was reversed in five studies [89, 94, 104, 115, 118] that compared dogs cared for by individuals with an income ≤ 2 minimum wages and those with guardians receiving > 2 minimum wages (OR = 0.74; 95% CI = 0.05–10.15). In both cases, however, the associations were not statistically significant (Additional File 8: Supplementary Fig. S3).

Five studies [9, 43, 84, 102, 108] evaluated water quality, showing no meaningful difference between treated and untreated water. Similarly, sewage collection [9, 102, 106, 122] and garbage collection [14, 40, 43, 84, 102, 103, 106, 108, 113] showed nonsignificant pooled associations (Additional File 8: Supplementary Figs. S4, S5).

Other variables relating to the occupation of the guardian; the type, quality and condition of the domicile; and the extent of street paving were examined by some publications [9, 56, 59, 64, 68, 80, 81, 84, 98, 103, 106], but the number of studies per variable was too small to support pooling or consistent conclusions.

#### Other variables

The association between dog sleeping locations and CVL was examined under several different contexts [57, 69, 84, 91, 99, 102, 104, 113, 115, 118, 123]. There was no difference in the odds of CVL between dogs that slept on the streets and those that slept inside the domicile (OR = 1.01; 95% CI = 0.58–1.76) (Additional File 9: Supplementary Fig. S1). However, there were higher odds of CVL among dogs that slept in the peridomicile compared with those that slept indoors (OR = 1.62; 95% CI = 1.17–2.24) (Additional File 9: Supplementary Fig. S2). Dogs free at night [104, 115, 118] tended to have lower odds of CVL than tied dogs, but the association was not statistically significant (OR = 0.80; 95% CI = 0.45–1.40) (Additional File 9: Supplementary Fig. S3).

Studies focused on the association between CVL and dog purpose [8, 63, 76, 81] or the mode of acquisition of the animal [104, 115, 118, 124] revealed that the probability of infection was high among guard dogs (OR = 1.76; 95% CI = 1.35–2.29) and those adopted from the streets (OR = 1.37; 95% CI = 0.89–2.10) (Additional File 9: Supplementary Figs. S4, S5). Furthermore, analysis of four studies [94, 104, 115, 118] suggested that the probability of CVL was higher among hunting dogs (OR = 1.56; 95% CI = 0.87–2.78) (Additional File 9: Supplementary Fig. S6).

Castration [75, 78, 84, 103, 116] was associated with higher odds of CVL, but not significantly (OR = 1.26; 95% CI = 0.55–2.89) (Additional File 9: Supplementary Fig. S7). Lower odds of infection were found among dogs that received regular veterinary care (OR = 0.76; 95% CI: 0.60–0.96) [13, 56, 80, 99, 102, 108, 114, 126], those fed formulated food (OR = 0.62; 95% CI = 0.31–1.26) [89, 90, 104, 105, 123, 126], and dogs that were dewormed (OR = 0.74; 95% CI = 0.47–1.17) [104, 115, 123] (Additional File 9: Supplementary Figs. S8–S10). Knowledge about the disease [13, 56, 63, 69, 77, 82, 94, 109, 112, 121] was associated with higher odds of CVL (OR = 1.38; 95% CI = 0.80–2.38) (Additional File 9: Supplementary Fig. S11).

Other variables were also associated with higher odds of infection, namely, the presence of sand flies in the household (OR = 1.48; 95% CI = 1.02–2.16) [63, 68, 69, 108]; previous cases of CVL in the domicile (OR = 1.53; 95% CI = 1.12–2.08) [12, 31, 38, 68, 81, 86, 102, 103] or in the neighborhood (OR = 2.36; 95% CI = 1.63–3.43) [63, 81, 95, 102, 109]; and previous occurrence of HVL in the domicile (OR = 1.83; 95% CI: 1.34–2.49) [12, 31, 63, 81, 106] (Additional File 9: Supplementary Figs. S12–S15).

### Associations that were not submitted to meta-analyses

For a large set of other variables, it was not possible to combine the data into summary association measures or obtain consistent patterns owing to the diversity of topics analyzed and the insufficient number of studies. These variables included dog-related characteristics (e.g., weight, hygiene, travel history, ectoparasite treatment, cause of death, and type of water consumed); ownership and management factors (e.g., care provided to the dog, duration and type of stewardship, recent acquisition or relocation, maintenance by the same guardian or within the same municipality, rotation between shelter stalls, quarantining of new animals, and removal from the Visceral Leishmaniasis Control Program in the previous 12 months); household characteristics (e.g., electricity services, presence of a septic tank, cleaning and disinfection practices, frequency of cleaning, use of toxic products, and presence of elderly people or children); and environmental factors (e.g., presence of animal feces, access to empty lots, villages or water reservoirs, distance from forested areas, presence of trees within a 10-m radius, water accumulation in the yard, proximity to railroad lines or ceramics factories, physical barriers, shade, environmental hygiene conditions, and interaction with wildlife) (Additional File 1).

## Discussion

This systematic review provides a comprehensive overview of factors associated with CVL occurrence and enhances understanding of infection determinants, awareness of research quality in the field, and identification of priorities for future studies.

The inclusion of additional studies in this review reinforces the findings of the 2013 review [11], showing slightly higher odds of infection among male dogs based on a dataset approximately three times larger. However, the lower strength of association in the combination of studies with control for confounders indicates that the observed result might not be attributable solely to aspects related to biological sex, such as immunological, hormonal, or even behavioral differences [127, 128].

Regarding the association between dog age and CVL, reduced positivity was observed among the younger, which may be explained by their lower cumulative lifetime exposure [111, 129]. However, pooled estimates from studies that controlled for confounding factors indicated an inverse association, suggesting that younger dogs may be as susceptible as, or even more susceptible than, older dogs when exposed to similar risk conditions. Taken together, these findings, combined with heterogeneity and the predominance of cross-sectional designs, limit stronger conclusions regarding age-related susceptibility.

A similar pattern was observed for dog breed. After stratification according to control for confounders, the direction of the association between breed and CVL changed, indicating slightly higher odds of infection among mixed-breed dogs. This finding may more adequately reflect the influence of breed on the disease, given that mixed-breed dogs are more likely to receive limited care and to be exposed to the vector [9]. However, genetic and immunological factors, as well as differences among specific breeds, may also influence susceptibility to infection, although these aspects remain poorly investigated [130].

In contrast, the association between short hair length and CVL was more consistent and supported by a moderate level of evidence. This classification reflects the stronger association observed in studies that controlled for confounders and the lower heterogeneity among measures of association. Coat length influences body temperature, moisture, and odor in dogs, all of which may affect sand fly attraction. Moreover, in short-haired dogs, the reduced physical barrier provided by the fur facilitates vector access to the skin [13, 27]. The findings suggest that short-haired dogs may represent a priority group for prevention and control interventions. For example, the use of deltamethrin-impregnated collars [131, 132] may be more effective when targeted toward these animals. However, specific studies should be designed to test the hypothesis that hair length acts as a possible modifier of the effects of intervention. Furthermore, there is no available evidence suggesting that topical insecticides are less efficient when applied to long-haired dogs.

Regarding dog size, a dose–response pattern was observed, with lower odds of infection in small dogs compared with medium-sized and, most notably, large dogs. However, the level of evidence was classified as low because the number of studies addressing this association was limited, and none controlled for confounding factors. If a lower probability of CVL among small dogs truly exists, it may be related to the reduced body surface area available for sand fly contact [95], as well as to confounding factors such as differences in breed, quality of care, exposure to the external environment, and adoption of protective measures [133].

Other variables identified with moderate levels of evidence are related to dog housing conditions and the degree of restraint. Studies that controlled for confounders, as well as those that examined analogous variables, indicated lower odds of infection among dogs kept indoors and those without free access to the streets. Although the strength of the pooled association was reduced and statistical significance was lost in publication bias assessments, the direction of the association between CVL and street access remained unchanged, reinforcing its consistency. These findings confirm that CVL transmission is not restricted to the household itself, but may operate at broader spatial scales, as dogs with free access to the streets are exposed to vectors circulating in surrounding areas. In addition, these animals may experience poorer living conditions [11, 12, 134, 135]. Accordingly, the implementation of educational actions and measures that hold guardians accountable for keeping dogs without adequate restriction to the domicile is warranted [123, 136, 137]. Such measures may contribute not only to reducing the risk of CVL but also to improving animal welfare.

The presence of vegetation in the vicinity of the domicile was also associated with higher odds of CVL, with a moderate level of evidence. Studies using different designs have reported a higher occurrence of HVL and CVL in domiciles located near forests and urban green areas [7, 82, 96, 138–140], reinforcing that the spatial configuration of urban environments can influence CVL risk beyond individual household characteristics [1]. The proximity of vegetated areas may facilitate interactions between sylvatic and peridomestic transmission cycles, thereby providing environmental conditions favorable for vector maintenance [138]. However, the strength of these associations may also reflect differences in the socioeconomic conditions of households located in peripheral areas. Taken together, these findings highlight the importance of considering both the immediate surroundings of residences and broader landscape characteristics, such as the presence of continuous green areas, when planning integrated strategies for VL prevention and control.

In contrast to the associations observed for environmental characteristics, the level of evidence for the association between the presence of chickens or chicken coops and CVL was classified as very low. This classification was mainly driven by publication bias, which attenuated the pooled measure of association, and by the fact that statistical significance was observed only in studies that did not control for confounding factors. Evidence consistently shows that chicken coops favor sand fly proliferation [138, 141]. However, considering the results obtained and the fact that chickens are refractory to infection and may exert a zooprophylactic effect by diverting blood meals from dogs and humans [142], the overall evidence for this association should be interpreted with caution [7]. Moreover, experimental evidence suggests that repeated blood meals from readily available hosts, even if refractory, may enhance sand fly infectiousness [143], highlighting the need for well-designed epidemiological and experimental studies to better elucidate this complex relationship.

Another association classified as having very low levels of evidence in this review refers to higher odds of infection among dogs living with other dogs. It has been argued that the presence of several dogs in the domicile augments the availability of food sources for sand flies and increases the risk of disease transmission [141]. Conversely, it can be hypothesized that a greater number of dogs may reduce the risk for an individual animal, as vectors would have more host options, potentially decreasing the frequency of bites per dog. In addition, spatial analyses [144] suggest that CVL risk may extend beyond the household itself, with neighborhood-level dog density measured in surrounding areas contributing to transmission dynamics, particularly in dense urban settings. In this context, it is noteworthy that in subgroup analyses, the cohort studies and case–control studies reported lower odds of CVL among dogs living with other dogs. Therefore, additional investigations, particularly using these study designs, are needed to clarify this relationship and to allow meta-analyses with more detailed categorizations of the number of animals.

With respect to the presence of other animals, the level of evidence for positive associations with CVL was classified as low for horses and very low for pigs or pigsties, cats, and rodents. In the case of horses, their presence may reflect broader spatial and environmental contexts, often associated with greener or peri-urban areas, while also representing an alternative food source that favors the maintenance of sand flies in the surrounding areas [105]. Evidence regarding pigs or pigsties remains scarce, and further research is needed, as sand fly proliferation and disease transmission may be related to organic waste rather than the animals themselves [141]. Additional studies are also warranted for the potential association between cats and CVL, given that cats may act as reservoirs of *Leishmania infantum* and are common in residential settings. Moreover, their roaming behavior may facilitate connections between wild and urban transmission cycles [145].

Another factor more robustly assessed in this review concerns the presence of ectoparasites, such as fleas and ticks, which were associated with CVL in studies that controlled for confounding factors. However, given the small number of studies and their heterogeneity, the level of evidence for this association was classified as low. Exposure of dogs to fleas and ticks can lead to various health problems, including suppression of the immune system and increased susceptibility to *L. infantum* infection. Moreover, the presence of ectoparasites may indicate the absence of repellent use, including products aimed at preventing sand fly bites, as well as unfavorable peridomiciliary conditions and inadequate levels of care provided by the guardian [105, 123]. Accordingly, effective and continuous ectoparasite control should be encouraged, as it may contribute both to animal health and to CVL prevention [146]. Nevertheless, confirmation of this association requires additional studies with greater methodological rigor and a broader range of species.

This review brought to light an extensive set of other variables (socioeconomic conditions, dog hair color, dog sleeping arrangements, dog purpose, mode of acquisition, hunting activity, castration status, access to veterinary care, type of food, deworming practices, guardians’ knowledge about the disease, presence of sand flies, and previous cases of CVL or HVL), although the limited number of studies for each precludes obtaining high levels of evidence or achieving more solid conclusions. Among these, socioeconomic factors stood out, with higher odds of infection observed in dogs cared for by guardians with low income or illiteracy. The presence of vectors and a history of previous HVL or CVL cases in the domicile or surrounding area were also associated with increased odds of infection. These associations suggest that spatial aggregation of infected hosts and vectors may contribute to clustered patterns of CVL transmission [7]. Finally, most indicators of substandard guardianship, such as inadequate feeding practices and lack of regular veterinary care, were associated with higher odds of CVL.

It should be acknowledged that the incorporation of new data in this updated review has enabled important advances compared with the earlier review, among which is the ability to compare association measures from studies with and without control for confounders. Nevertheless, it is important to highlight that there was a predominance of studies without control for confounders, while those that performed control addressed only a few specific variables. Furthermore, most studies that controlled for confounders reported only the results of the final multivariable regression models and did not present the intermediate steps of the modeling process, which is inconsistent with the recommendations of the Strengthening the Reporting of Observational Studies in Epidemiology (STROBE) guidelines [147]. The uneven control for confounders not only prevented the development of robust conclusions regarding the explanatory factors identified for some variables but also the establishment of higher levels of evidence. Thus, it is worth reiterating the need to refine methodological approaches that allow adequate control of confounding factors, as well as the exploration of interactions between variables and the potential influence of control strategies on the associations examined.

Despite improvements in the quality of recent publications with respect to bias susceptibility and reporting practices, important limitations in the available evidence persist and should be addressed in the research agenda. In particular, CVL diagnosis continues to rely predominantly on serological methods, with limited use of molecular techniques either alone or in combination with serology. Moreover, cohort and case–control studies should be prioritized to enable more robust causal inference and a better understanding of disease progression over time [11]. Given the costs and long follow-up periods required for longitudinal studies, support from governmental agencies, public health institutions, and policymaking organizations through adequate funding will be essential. Case–control studies could also benefit from greater use of underexploited digital resources, such as databases maintained by municipal governments, state health departments, veterinary hospitals, and epidemiological surveillance services, enabling more agile and cost-effective investigation of specific contexts [148]. Finally, as most evidence originates from studies conducted in Brazil, further research in other countries across the Americas is needed to expand the generalizability of these findings to different epidemiological settings.

This review has potential limitations relating to the diversity of the studies included in the meta-analyses, including the scope of the associations investigated, intrinsic issues with study designs, and performance of diagnostic tests [149]. Although subgroup analyses were performed, nearly all stratifications were unable to explain the observed heterogeneity. Therefore, association measures obtained in different contexts and with dissimilar levels of bias susceptibility were often combined. This strategy was adopted to identify consistent patterns for the associations studied, which would not be possible through narrative synthesis alone.

## Conclusions

This review expands current knowledge on factors associated with CVL in the Americas, providing stronger evidence for some variables, including dog sex, hair length, degree of restraint, and the presence of vegetation in or near the domicile. In addition, it synthesizes emerging evidence on the role of environmental, social, and animal care factors in *Leishmania infantum* infection. Together, these findings enhance understanding of CVL epidemiology and may inform the development of more effective public health policies for disease prevention and control.

## Supplementary Information

**Additional file 1**. Summary of included articles and main characteristics.

**Additional file 2**. Analysis of the methodological quality of the studies included in the 2013 review (search completed up to September 2011) and the current review (studies published from October 2011 up to June 2024), using the JBI tool.

**Additional file 3**. Results of meta-analysis: assessment of heterogeneities with description of Q test, *P*-values and I2 statistics.

**Additional file 4**. GRADE Summary of Findings table for the main exposures associated with canine visceral leishmaniasis.

**Additional file 5**. Forest plots of analyzed variables (Figs. S1–S22).

**Additional file 6**. Forest plots of variables stratified by subgroups (Figs. S1–S12).

**Additional file 7**. Funnel plots for assessment of publication bias (Figs. S1–S9).

**Additional file 8**. Forest plots of socioeconomic variables (Figs. S1–S5).

**Additional file 9**. Forest plots of other variables (Figs. S1–S15).

## Data Availability

All data supporting the findings of this study are available within the paper and the Supplementary files.

## Abbreviations

CI: Confidence interval
CVL: Canine visceral leishmaniasis
DPP: Dual-path platform
ELISA: Enzyme-linked immunosorbent assay
GRADE: Grading of Recommendations Assessment, Development and Evaluation
HVL: Human visceral leishmaniasis
IFAT: Immunofluorescence antibody test
MOOSE: Meta-analysis of Observational Studies in Epidemiology
NDVI: Normalized difference vegetation index
OR: Odds ratio
PRISMA: Preferred Reporting Items for Systematic Reviews and Meta-Analyses
STROBE: Strengthening the Reporting of Observational Studies in Epidemiology
