# Supplementary material for: Risk and protective factors for canine visceral leishmaniasis in the Americas: a systematic review update with meta-analysis
Source: Parasit Vectors. 2026 Mar 18;19:185. doi: 10.1186/s13071-026-07325-0 (PMC13122873; doi:10.1186/s13071-026-07325-0)
Supplement: Supplementary file 2 — Additional file 2. Analysis of the methodological quality of the studies included in the 2013 review (search completed up to September 2011) and the current review (studies published from October 2011 up to June 2024), using the JBI tool. [file 13071_2026_7325_MOESM2_ESM.docx]

**Additional file 2 - Analysis of the methodological quality of the studies included in the 2013 review (search completed up to September 2011) and the current review (studies published from October 2011 up to June 2024), using the JBI tool.**

**Cohort Studies**

| **First Author/ Year** | **Title** | **Review** | **Q1** | **Q2** | **Q3** | **Q4** | **Q5** | **Q6** | **Q7** | **Q8** | **Q9** | **Q10** | **Q11** | **Total** | **Quality** |
| --- | --- | --- | --- | --- | --- | --- | --- | --- | --- | --- | --- | --- | --- | --- | --- |
| Soares et al., 2013 | Aspectos epidemiológicos da leishmaniose visceral em uma coorte de cães em Juatuba-MG, de 2010 a 2011 | Current | **Y** | **Y** | **Y** | **Y** | **Y** | **Y** | **Y** | **Y** | **N** | **N** | **Y** | **9** | **Moderate** |
| Silva, 2023 | Socioeconomic Status of Guardians as a Risk Factor for Canine Visceral Leishmaniasis:  A Cohort Study in an Endemic Area of the Federal District, Brazil | Current | **Y** | **Y** | **Y** | **Y** | **Y** | **Y** | **Y** | **Y** | **N** | **N** | **Y** | **9** | **Moderate** |
| Lopes et al., 2016 | Transmission of visceral leishmaniasis in dogs in a risk area of the metropolitan  region of Belo Horizonte, Minas Gerais, Brazil | Current | **Y** | **Y** | **Y** | **Y** | **Y** | **Y** | **Y** | **Y** | **Y** | **N** | **Y** | **10** | **High** |
| Coura-Vital et al., 2013 | Canine visceral leishmaniasis: Incidence and risk factors for infection in a cohort study in Brazil | Current | **Y** | **Y** | **Y** | **Y** | **Y** | **Y** | **Y** | **Y** | **Y** | **Y** | **Y** | **11** | **High** |
| Coura-Vital et al., 2013 | Risk Factors for Seroconversion by Leishmania infantum in a Cohort of Dogs from an Endemic Area of Brazil | Current | **Y** | **Y** | **Y** | **Y** | **Y** | **Y** | **Y** | **Y** | **Y** | **Y** | **Y** | **11** | **High** |
| Barboza, et al. 2006 | Estudo de coorte em áreas de risco para leishmaniose visceral canina, em municípios da Região Metropolitana de Salvador, Bahia, Brasil | 2013 | **U** | **U** | **Y** | **Y** | **Y** | **Y** | **Y** | **U** | **N** | **N** | **Y** | **6** | **Moderate** |
| Wilke, 2005 | Avaliação das atividades de controle da Leishmaniose  Visceral Canina no Município de Sabará, Minas Gerais (TESE) | 2013 | **U** | **U** | **Y** | **N** | **N** | **Y** | **Y** | **Y** | **N** | **N** | **N** | **4** | **Low** |
| Paranhos-Silva et al. 1998 | Cohort study on canine emigration and leishmania  infection in an endemic area for american visceral  leishmaniasis. Implications for the disease control | 2013 | **U** | **U** | **Y** | **N** | **N** | **Y** | **Y** | **Y** | **N** | **N** | **N** | **4** | **Low** |
| Moreira-Jr et al., 2003 | Peridomestic risk factors for canine leishmaniasis in urban dwellings: new findings from a prospective study  in Brazil | 2013 | **Y** | **Y** | **Y** | **Y** | **Y** | **Y** | **Y** | **Y** | **Y** | **Y** | **Y** | **11** | **High** |

**Legend:** **Y= Yes; N= No; U=unclear.** **Q1.** Were the two groups similar and recruited from the same population? **Q2.** Were the exposures measured similarly to assign people to both exposed and unexposed groups? **Q3.** Was the exposure measured in a valid and reliable way? **Q4.** Were confounding factors identified? **Q5.** Were strategies to deal with confounding factors stated? **Q6.** Were the groups/participants free of the outcome at the start of the study (or at the moment of exposure)? **Q7.** Were the outcomes measured in a valid and reliable way? **Q8.** Was the follow up time reported and sufficient to be long enough for outcomes to occur? **Q9.** Was follow up complete, and if not, were the reasons to loss to follow up described and explored? **Q10.** Were strategies to address incomplete follow up utilized? **Q11.** Was appropriate statistical analysis used?

**Quality**:

“Low”: up to five “Yes” responses

“Moderate”: six to nine “Yes” responses

“High”: ten or eleven “Yes” responses

**Case control studies**

| **First Author/ Year** | **Title** | **Review** | **Q1** | **Q2** | **Q3** | **Q4** | **Q5** | **Q6** | **Q7** | **Q8** | **Q9** | **Q10** | **Total** | **Quality** |
| --- | --- | --- | --- | --- | --- | --- | --- | --- | --- | --- | --- | --- | --- | --- |
| Paz et al., 2013 | Ectoparasites and anti-Leishmania antibodies: Association inan observational case–control study of dogs from a Brazilianendemic area | Current | **U** | **Y** | **Y** | **Y** | **Y** | **N** | **N** | **Y** | **Y** | **N** | **6** | **Moderate** |
| Silva et al., 2012 | Factors associated with Leishmania chagasi infection in domestic dogs from Teresina, State of Piauí, Brazil | Current | **U** | **Y** | **Y** | **Y** | **Y** | **Y** | **Y** | **Y** | **U** | **Y** | **8** | **Moderate** |
| Seixas et al., 2012 | Positividade para leishmaniose visceral canina: existem fatores caninos que contribuem? | Current | **Y** | **Y** | **Y** | **Y** | **Y** | **N** | **N** | **Y** | **Y** | **N** | **7** | **Moderate** |
| Villegas, 2015 | Fatores de risco de Leishmaniose Visceral em cães no município de Panorama, Estado de São Paulo, SP, Brasil (TESE) | Current | **U** | **Y** | **Y** | **Y** | **Y** | **Y** | **Y** | **Y** | **U** | **Y** | **8** | **Moderate** |
| Paz et al., 2010 | Association between the prevalence of infestation by Rhipicephalus sanguineus and Ctenocephalides felis felis and the presence of anti-Leishmania antibodies: a case-control study in dogs from a Brazilian endemic area | 2013 | **Y** | **Y** | **Y** | **Y** | **Y** | **N** | **N** | **Y** | **U** | **Y** | **7** | **Moderate** |

**Legend: Y= Yes; N= No; U= Unclear; Q1.** Were the groups comparable other than the presence of disease in cases or the absence of disease in controls? **Q2.** Were cases and controls matched appropriately? **Q3.** Were the same criteria used for identification of cases and controls? **Q4.** Was exposure measured in a standard, valid and reliable way? **Q5.** Was exposure measured in the same way for cases and controls? **Q6.** Were confounding factors identified? **Q7.** Were strategies to deal with confounding factors stated? **Q8.** Were outcomes assessed in a standard, valid and reliable way for cases and controls? **Q9.** Was the exposure period of interest long enough to be meaningful? **Q10.** Was appropriate statistical analysis used?

**Quality:**

“Low”: up to four “Yes” responses

“Moderate”: five to eight “Yes”

“High”: nine or ten “Yes” responses

**Cross-Sectional Studies**

| **First Author/ Year** | **Title** | **Review** | **Q1** | **Q2** | **Q3** | **Q4** | **Q5** | **Q6** | **Q7** | **Q8** | **Total** | **Quality** |
| --- | --- | --- | --- | --- | --- | --- | --- | --- | --- | --- | --- | --- |
| Law, 2022 | Análise ecoepideiológica da leishmaniose visceral caninca no município de Foz do Iguaçu entre 2018 e 2019 (TESE) | Current | **Y** | **U** | **Y** | **Y** | **Y** | **Y** | **Y** | **Y** | **7** | **High** |
| Benitez et al., 2018 | Spatial and simultaneous seroepidemiology of anti-Leishmania spp. antibodies in dog owners and their dogs from randomly selected households in a major city of southern Brazil | Current | **Y** | **Y** | **Y** | **Y** | **Y** | **Y** | **Y** | **Y** | **7** | **High** |
| Oliveira et al., 2015 | Canine visceral leishmaniases case investigation in the Jacare regionr of Niteroi, Rio de Janerio, Brazil | Current | **U** | **Y** | **Y** | **Y** | **Y** | **Y** | **Y** | **Y** | **6** | **Moderate** |
| Carvalho et al., 2019 | Factors associated with Leishmania spp. infection in domestic dogs from an emerging area of high endemicity for visceral leishmaniasis in Central-Western Brazil | Current | **Y** | **Y** | **Y** | **Y** | **Y** | **Y** | **Y** | **Y** | **7** | **High** |
| Gomes, 2013 | Fatores de risco e análise espacial para a leishmaniose visceral no município de Juazeiro - Bahia - Brasil (TESE) | Current | **Y** | **Y** | **Y** | **Y** | **N** | **N** | **Y** | **N** | **5** | **Moderate** |
| Teixeira et al., 2020 | A cross-sectional approach including dog owner characteristics as predictors of visceral leishmaniasis infection in dogs | Current | **Y** | **U** | **Y** | **Y** | **Y** | **Y** | **Y** | **Y** | **7** | **High** |
| Araújo et al., 2016 | Epidemiological aspects and risk factors for infection by Leishmania infantum chagasi in dogs from municipality of Petrolina,  Northeastern Brazil | Current | **Y** | **Y** | **Y** | **Y** | **Y** | **Y** | **Y** | **Y** | **8** | **High** |
| Santana, 2011 | Fatores epidemiológicos associados e novas abordagens diagnósticas para Leishmaniose e Babesiose Canina no município de São Luís - MA, Brasil - (TESE) Capítulo I: Soroprevalência e variáveis epidemiológicas associadas à Leishmaniose Visceral Canina em área endêmica no município de São Luiz, Maranhão, Brasil | Current | **Y** | **Y** | **Y** | **Y** | **Y** | **Y** | **Y** | **Y** | **8** | **High** |
| Toepp et al., 2019 | Comorbid infections induce progression of visceral leishmaniasis | Current | **Y** | **U** | **Y** | **Y** | **Y** | **Y** | **Y** | **Y** | **7** | **High** |
| Evaristo et al., 2020 | Canine leishmaniasis in the semi-arid region of Pernambuco, northeastern Brazil: epidemiology, factors associated with seropositivity and spatial analysis | Current | **Y** | **Y** | **Y** | **Y** | **Y** | **Y** | **Y** | **Y** | **8** | **High** |
| Fernandes et al., 2016 | Risk factors associated with seropositivity for Leishmania spp. and Trypanosoma cruzi in dogs in the state of Paraiba, Brazil | Current | **Y** | **U** | **Y** | **Y** | **Y** | **Y** | **Y** | **Y** | **7** | **High** |
| Michelin et al., 2018 | Factors associated with positivity for canine visceral leishmaniosis in an endemic area in Brazil | Current | **Y** | **Y** | **Y** | **Y** | **N** | **N** | **Y** | **N** | **5** | **Moderate** |
| Almeida et al., 2012 | Canine visceral leishmaniasis: seroprevalence and risk factors in Cuiabá, Mato Grosso, Brazil | Current | **Y** | **Y** | **Y** | **Y** | **Y** | **Y** | **Y** | **Y** | **8** | **High** |
| Borges, 2011 | Fatores de risco associados ao perfil sorológico da Leishmaniose visceral em cães, Montes Claros/MG (TESE) | Current | **Y** | **Y** | **Y** | **Y** | **Y** | **Y** | **Y** | **Y** | **8** | **High** |
| Braz et al., 2021 | Factors associated with Leishmania infection in dogs and geospatial analysis in the Sertão of Paraíba, Northeast Brazil | Current | **Y** | **Y** | **Y** | **Y** | **Y** | **Y** | **Y** | **Y** | **8** | **High** |
| Varjão et al., 2021 | Spatial distribution of canine Leishmania infantum infection in a municipality with endemic human leishmaniasis in Eastern Bahia, Brazil | Current | **Y** | **Y** | **Y** | **Y** | **N** | **N** | **Y** | **N** | **5** | **Moderate** |
| Spindola et al., 2024 | Canine visceral leishmaniasis: Seroprevalence and georeferencing in the  state of Santa Catarina, Brazil | Current | **Y** | **U** | **Y** | **Y** | **N** | **N** | **Y** | **N** | **4** | **Moderate** |
| Costa et al., 2018 | Canine visceral leishmaniasis in Araçatuba, state of São Paulo, Brazil, and its relationship with characteristics of dogs and their owners: a cross-sectional and spatial analysis using a geostatistical approach | Current | **Y** | **Y** | **Y** | **Y** | **Y** | **Y** | **Y** | **Y** | **8** | **High** |
| Barbosa et al., 2022 | Factors associated with Leishmania infantum infection in dogs from urban  areas endemic for visceral leishmaniasis in Brazil | Current | **Y** | **Y** | **Y** | **Y** | **Y** | **Y** | **Y** | **Y** | **8** | **High** |
| Leote et al., 2021 | The first case of canine visceral leishmaniasis in the southern region of Santa  Catarina, an emerging focus of visceral leishmaniasis in Brazil: regional report or  reflection of the reality of a country? | Current | **Y** | **Y** | **Y** | **Y** | **N** | **N** | **Y** | **N** | **5** | **Moderate** |
| Antônio et al., 2011 | Canine visceral leishmaniasis in the Krenak indigenous community, Resplendor, Minas Gerais State, Brazil, 2007 | Current | **Y** | **Y** | **Y** | **Y** | **N** | **N** | **Y** | **N** | **5** | **Moderate** |
| Veloso et al., 2021 | Socio-economic and environmental factors associated with the occurrence of canine infection by Leishmania infantum in Teresina, Brazil | Current | **Y** | **Y** | **Y** | **Y** | **Y** | **Y** | **Y** | **Y** | **8** | **High** |
| Brito et al., 2016 | Canine visceral leishmaniasis in the Northeast Region of Brazil | Current | **Y** | **Y** | **Y** | **Y** | **N** | **N** | **Y** | **N** | **5** | **Moderate** |
| Nascimento, 2011 | Aspectos epidemiológicos da leishmaniose visceral canina nas regiões administrativas Lago Norte e Sobradinho II do Distrito Federal-DF (TESE) | Current | **Y** | **Y** | **Y** | **Y** | **Y** | **Y** | **Y** | **Y** | **8** | **High** |
| Gama, 2014 | Prevalência e fatores associados à Leishmaniose Canina na região nordeste do município de Divinópolis, MG (TESE) | Current | **Y** | **Y** | **Y** | **Y** | **Y** | **Y** | **Y** | **Y** | **8** | **High** |
| Leal et al., 2018 | Risk profile for Leishmania infection in dogs coming from an area of visceral leishmaniasis reemergence | Current | **Y** | **Y** | **Y** | **Y** | **Y** | **Y** | **Y** | **Y** | **8** | **High** |
| Lima de Sá, 2019 | Epidemiologia da Leishmaniose Visceral Canina em Paraupebas, Pará, Brasil (TESE) | Current | **Y** | **Y** | **Y** | **Y** | **N** | **N** | **Y** | **N** | **5** | **Moderate** |
| Souza, 2021 | Detecção de anticorpos anti-Leishmania infantum em cães procedentes de diferentes mesorregiões do estado de Pernambuco (TESE) | Current | **Y** | **Y** | **Y** | **Y** | **N** | **N** | **Y** | **N** | **5** | **Moderate** |
| Barbosa, 2015 | Seroepidemiological Survey of Canine Leishmania Infections from  Peripheral Areas in Natal, Northeast Brazil | Current | **Y** | **Y** | **Y** | **Y** | **N** | **N** | **Y** | **N** | **5** | **Moderate** |
| Reis, 2018 | Fatores associados à ocorrência de Leishmaniose Visceral Canina no município de Araguaína, Tocantins (TESE) - Capítulo 2 | Current | **Y** | **Y** | **Y** | **Y** | **N** | **N** | **Y** | **N** | **5** | **Moderate** |
| Costa, 2020 | Levantamento soroepidemiológico da Leishamaniose Visceral Canina em área não endêmica (TESE) | Current | **Y** | **Y** | **Y** | **Y** | **N** | **N** | **Y** | **N** | **5** | **Moderate** |
| Jaimes-Dueñez et al., 2023 | Epidemiological features of Leishmania infantum in dogs (Canis lupus  familiaris) suggest a latent risk of visceral leishmaniasis in the metropolitan  area of Bucaramanga, Santander, Eastern Colombia | Current | **Y** | **Y** | **Y** | **Y** | **N** | **N** | **Y** | **N** | **5** | **Moderate** |
| Pastor-Santiago et al., 2012 | American Visceral Leishmaniasis in Chiapas, Mexico | Current | **Y** | **Y** | **Y** | **Y** | **N** | **N** | **Y** | **N** | **5** | **Moderate** |
| Júnior, 2013 | Estudo clínico e soroepidemiológico da Leishmaniose Visceral Canina em Juiz de Fora, MG | Current | **Y** | **Y** | **Y** | **Y** | **Y** | **Y** | **Y** | **Y** | **8** | **High** |
| Spada et al., 2020 | Risk factors associated with Leishmania exposure among dogs in a rural area of Ilha Solteira, SP, Brazil | Current | **Y** | **Y** | **Y** | **Y** | **Y** | **Y** | **Y** | **Y** | **8** | **High** |
| Costa et al., 2014 | Awareness of visceral leishmaniasis and its relationship to canine infection in riverside endemic areas in Northeastern Brazil | Current | **Y** | **Y** | **Y** | **Y** | **Y** | **Y** | **Y** | **Y** | **8** | **High** |
| Martins, 2018 | Fatores associados à ocorrência de Leishmaniose Visceral em cães de área urbana após aplicação de medidas de proteção individual (TESE) - Capítulo 2 | Current | **Y** | **Y** | **Y** | **Y** | **Y** | **Y** | **Y** | **Y** | **8** | **High** |
| Esch et al., 2012 | Preventing Zoonotic Canine Leishmaniasis in Northeastern Brazil: Pet Attachment and Adoption of Community Leishmania Prevention | Current | **Y** | **Y** | **U** | **Y** | **Y** | **Y** | **Y** | **Y** | **7** | **High** |
| Penaforte et al., 2013 | Leishmania infection in a population of dogs: an epidemiological investigation relating to visceral leishmaniasis control | Current | **Y** | **Y** | **Y** | **Y** | **Y** | **Y** | **Y** | **Y** | **8** | **High** |
| Franco e Silva, 2018 | Avaliação epidemiológica da leishmaniose visceral canina na Paraíba - (TESE) Capítulo I: Avaliação epidemiológica da Leishmaniose Visceral Canina em Microrregião do Agreste Paraibano, Brasil | Current | **Y** | **Y** | **U** | **Y** | **Y** | **Y** | **Y** | **Y** | **7** | **High** |
| Franco e Silva, 2018 | Avaliação epidemiológica da leishmaniose visceral canina na Paraíba - (TESE) Capítulo II: Prevalência, fatores de risco e distribuição espacial da Leishmaniose Visceral Canina no município de São José de Espnharas, Sertão Paraibano, Brasil | Current | **Y** | **Y** | **Y** | **Y** | **Y** | **Y** | **Y** | **Y** | **8** | **High** |
| Freitas, 2017 | Epidemiologia da Leishmaniose Visceral Canina no município de Unaí, Minas Gerais, nos anos de 2012 a 2016 (TESE) | Current | **Y** | **Y** | **Y** | **Y** | **Y** | **Y** | **Y** | **Y** | **8** | **High** |
| Chiyo et al., 2023 | Cross-sectional spatial and epidemiological analysis of canine visceral  leishmaniasis cases in the triple border region, Brazil, Argentina and  Paraguay, between 2015 and 2020 | Current | **Y** | **Y** | **Y** | **Y** | **Y** | **Y** | **Y** | **Y** | **8** | **High** |
| Borges, 2011 | Prevalência e fatores de risco para Leishmaniose visceral em cães de Juatuba, Minas Gerais, 2010 (TESE) | Current | **Y** | **Y** | **Y** | **Y** | **N** | **N** | **Y** | **N** | **5** | **Moderate** |
| Gonçalves, 2014 | Prevalência, distribuição e identificação de prováveis fatores de risco para Leishmaniose Visceral Canina em Camaçari -BA (TESE) | Current | **Y** | **Y** | **Y** | **Y** | **N** | **N** | **Y** | **N** | **5** | **Moderate** |
| Figueiredo et al., 2014 | Fatores de Risco e Classificação Clínica Associados À Soropositividade para Leishmaniose Visceral Canina | Current | **Y** | **Y** | **Y** | **Y** | **N** | **N** | **Y** | **N** | **5** | **Moderate** |
| Paternina- Gómez et al., 2013 | High prevalence of infection with Leishmania (Kinetoplastea: Trypanosomatidae) in dogs in northern Colombia | Current | **Y** | **U** | **Y** | **Y** | **N** | **N** | **Y** | **N** | **4** | **Moderate** |
| Martins, 2023 | Análise epidemiológica da leishmaniose visceral canina no Distrito Federal (2005-2022): Investigação dos fatores associados à soroprevalência da LVC (TESE) | Current | **Y** | **Y** | **Y** | **Y** | **Y** | **Y** | **Y** | **Y** | **8** | **High** |
| Bernardino et al., 2020 | High seroprevalence and associated factors for visceral leishmaniasis in dogs in a transmission area of Paraíba state, Northeastern Brazil. | Current | **Y** | **Y** | **Y** | **Y** | **Y** | **Y** | **Y** | **Y** | **8** | **High** |
| Meirelles, 2022 | Fatores epidemiológicos associados à leishmaniose visceral canina e urbanização em Camaçari - BA de 2011 a 2015 (TESE) | Current | **Y** | **Y** | **Y** | **Y** | **Y** | **Y** | **Y** | **Y** | **8** | **High** |
| Alves, 2016 | Ocorrência da Leishmaniose Visceral em cães e gatos em abrigos de animais de Ilha Soteira, SP (TESE) | Current | **Y** | **Y** | **Y** | **Y** | **N** | **N** | **Y** | **N** | **5** | **Moderate** |
| Albarracin et al., 2011 | Seroprevalencia de Leishmaniasis canina en las localidades TOPO I, TOPO II y las Cumbres de Zamora de la parroquia capital de San Mateo municipio Bolívar, estado Aragua | Current | **Y** | **Y** | **Y** | **Y** | **N** | **N** | **Y** | **N** | **5** | **Moderate** |
| Carvalho et al., 2020 | Canine visceral leishmaniasis: perception, prevalence, and spatial distribution in municipality of Nossa Senhora do Livramento, Mato Grosso, Brazil | Current | **Y** | **Y** | **Y** | **Y** | **Y** | **Y** | **Y** | **Y** | **8** | **High** |
| Santos et al., 2023 | Spatial analysis of the risk for canine visceral leishmaniasis in an urban area in the State of Sergipe | Current | **Y** | **Y** | **Y** | **Y** | **N** | **N** | **Y** | **N** | **5** | **Moderate** |
| Curi et al., 2014 | Factors Associated with the Seroprevalence of Leishmaniasis in Dogs Living around Atlantic Forest Fragments | Current | **Y** | **Y** | **Y** | **Y** | **N** | **N** | **Y** | **N** | **5** | **Moderate** |
| Júnior et al., 2015 | Epidemiology of canine leishmaniasis in southern Bahia, Brazil | Current | **Y** | **Y** | **Y** | **Y** | **Y** | **Y** | **Y** | **Y** | **8** | **High** |
| Soares et al., 2022 | Responsible companion animal  guardianship is associated with canine visceral leishmaniasis: an analytical cross-sectional survey in an urban area of southeastern Brazil | Current | **Y** | **Y** | **Y** | **Y** | **Y** | **Y** | **Y** | **Y** | **8** | **High** |
| Silva et al., 2021 | Differentials in the epidemiological profile of canine visceral leishmaniasis in the semi-arid region of Paraíba, Brazil | Current | **Y** | **Y** | **Y** | **Y** | **Y** | **Y** | **Y** | **Y** | **8** | **High** |
| Silva et al., 2018 | Seroprevalence and risk factors associated with canine visceral leishmaniasis in the State of Paraíba, Brazil | Current | **Y** | **Y** | **Y** | **Y** | **Y** | **Y** | **Y** | **Y** | **8** | **High** |
| Chagas, 2017 | Leishmaniose visceral canina: perfil epidemiológico do Distrito Federal, 2013 a 2017 (TESE) | Current | **Y** | **U** | **Y** | **Y** | **N** | **N** | **Y** | **N** | **4** | **Moderate** |
| Silva et al., 2017 | Leishmaniose visceral em cães de assentamentos rurais | Current | **Y** | **Y** | **Y** | **Y** | **Y** | **Y** | **Y** | **Y** | **8** | **High** |
| Silva et al., 2023 | Canine visceral leishmaniasis: risk factors and spatial  analysis in an endemic area of Northeastern Brazil | Current | **Y** | **Y** | **Y** | **Y** | **Y** | **Y** | **Y** | **Y** | **8** | **High** |
| Lopes et al., 2014 | Seroprevalence and risk factors associated with visceral leishmaniasis in dogs in Jaciara, State of Mato Grosso | Current | **U** | **Y** | **Y** | **Y** | **Y** | **Y** | **Y** | **Y** | **7** | **High** |
| Rodrigues et al., 2020 | Spatial and seroepidemiology of canine visceral leishmaniasis in an endemic Southeast Brazilian area | Current | **Y** | **Y** | **Y** | **Y** | **N** | **N** | **Y** | **N** | **5** | **Moderate** |
| Andrade, 2014 | Soroprevalência, fatores e aspectos clínicos associados à Leishmaniose Visceral Canina em Goiania, Estado de Pernambuco, Brasil (TESE) | Current | **Y** | **Y** | **Y** | **Y** | **N** | **N** | **Y** | **N** | **5** | **Moderate** |
| Abrantes et al., 2018 | Fatores ambientais associados à ocorrência de leishmaniose visceral canina em uma área de recente introdução da doença no  Estado do Rio de Janeiro, Brasil | Current | **Y** | **Y** | **Y** | **Y** | **Y** | **Y** | **Y** | **Y** | **8** | **High** |
| Pohren, 2021 | Detecção de anticorpos anti-Leishmania infantum em cães procedentes de diferentes mesorregiões do estado de Pernambuco (TESE) | Current | **Y** | **Y** | **Y** | **Y** | **N** | **N** | **Y** | **N** | **5** | **Moderate** |
| Soccol et al., 2017 | More than the eyes can see: The worrying scenario of canine leishmaniasis in the Brazilian side of the triple border | Current | **Y** | **Y** | **Y** | **Y** | **Y** | **Y** | **Y** | **Y** | **8** | **High** |
| Almeida et al., 2009 | Inquérito soroepidemiológico de leishmaniose canina em  áreas endêmicas de Cuiabá, Estado de Mato Grosso | 2013 | **Y** | **Y** | **Y** | **Y** | **N** | **N** | **Y** | **N** | **5** | **Moderate** |
| Almeida, 2010 | Prevalência e epidemiologia da leishmaniose visceral em cães e humanos, na cidade de  Cuiabá, Mato Grosso, Brasil | 2013 | **Y** | **U** | **Y** | **Y** | **N** | **N** | **Y** | **N** | **4** | **Moderate** |
| Boraschi, 2007 | Inquérito sobre o conhecimento da população da Cidade de Três Lagoas – MS sobre Leishmaniose Visceral (TESE) | 2013 | **Y** | **Y** | **N** | **N** | **N** | **N** | **Y** | **N** | **3** | **Low** |
| Barbosa et al., 2010 | Soroprevalência e variáveis epidemiológicas associadas à leishmaniose visceral canina em área endêmica no município de São Luís, Maranhão, Brasil | 2013 | **Y** | **Y** | **Y** | **Y** | **Y** | **Y** | **Y** | **Y** | **8** | **High** |
| Papa, 2010 | Perfil Epidemiológico da Leishmaniose Visceral em Cães  Diagnosticados no Laboratório da Escola de Veterinária da Universidade Federal de Minas Gerais, Belo Horizonte, 2004 a 2008 (TESE) | 2013 | **Y** | **Y** | **Y** | **Y** | **N** | **N** | **Y** | **N** | **5** | **Moderate** |
| Dantas-Torres et al., 2006 | Seroepidemiological survey on canine leishmaniasis among dogs from an urban area of Brazil | 2013 | **Y** | **Y** | **Y** | **Y** | **N** | **N** | **Y** | **N** | **5** | **Moderate** |
| Dantas-Torres et al., 2010 | Cutaneous and visceral leishmaniasis in dogs from a rural community in northeastern  Brazil. | 2013 | **Y** | **Y** | **Y** | **Y** | **N** | **N** | **Y** | **N** | **5** | **Moderate** |
| Julião et al. 2007 | Investigação de áreas de risco como metodologia complementar ao controle da leishmaniose visceral canina. | 2013 | **Y** | **Y** | **Y** | **Y** | **Y** | **Y** | **Y** | **Y** | **8** | **High** |
| Braga, 2007 | Aspectose Epidemiológicos, clínicos e imunológicos de  cães (Canis familiaris) (Linnaeus, 1758) com Infecção por Leishmania (leishmania) chagasi (Cunha & Chagas, 1937) provenientes do Município de Imperatriz, região sudoeste do Estado do Maranhão, Brasil (TESE) | 2013 | **Y** | **Y** | **Y** | **Y** | **N** | **N** | **Y** | **N** | **5** | **Moderate** |
| Santos, 2008 | Fatores Associados à Soropositividade para Leishmaniose Visceral Canina no Município de Piraquê, Estado do Tocantins, Brasil (TESE) | 2013 | **Y** | **Y** | **Y** | **Y** | **Y** | **Y** | **Y** | **Y** | **8** | **High** |
| Vidal, 2008 | Aspectos Epidemiológicos da Leishmaniose Visceral Canina em Campina Grande, Brasil (TESE) | 2013 | **Y** | **Y** | **Y** | **Y** | **N** | **N** | **Y** | **N** | **5** | **Moderate** |
| Santos et al., 2010 | Prevalence of anti-Leishmania spp antibodies in dogs from Garanhuns, in the middle scrub zone (Agreste) of Pernambuco | 2013 | **Y** | **Y** | **Y** | **Y** | **N** | **N** | **Y** | **N** | **5** | **Moderate** |
| França-Silva et al., 2003 | Epidemiology of canine visceral leishmaniosis in the endemic  area of Montes Claros municipality, Minas Gerais State, Brazil. | 2013 | **Y** | **Y** | **Y** | **Y** | **N** | **N** | **Y** | **N** | **5** | **Moderate** |
| Naveda et al., 2006 | Aspectos epidemiológicos da leishmaniose visceral canina no município de Pedro Leopoldo, Minas Gerais, 2003 | 2013 | **Y** | **Y** | **Y** | **U** | **N** | **N** | **Y** | **N** | **4** | **Moderate** |
| Oliveira et al., 2010 | Soroprevalência e fatores de risco para leishmaniose visceral canina na área endêmica de Dias D’Ávila, Estado da Bahia, Brasil | 2013 | **Y** | **Y** | **Y** | **Y** | **N** | **N** | **Y** | **N** | **5** | **Moderate** |
| Gomes, 2007 | Prevalência da Leishmaniose Visceral Canina na Zona  Rural do Município de Varzelândia, Minas Gerais, Brasil, 2005 (TESE) | 2013 | **Y** | **Y** | **Y** | **Y** | **N** | **N** | **Y** | **N** | **5** | **Moderate** |
| Azevedo et al., 2008 | Canine visceral leishmaniasis evaluation in Poxoreo, Mato Grosso State, Brazil | 2013 | **Y** | **Y** | **Y** | **Y** | **N** | **N** | **Y** | **N** | **5** | **Moderate** |
| Cabrera et al., 2003 | Canine visceral leishmaniasis in Barra de Guaratiba, Rio de Janeiro, Brazil: assessment of risk factors | 2013 | **U** | **Y** | **Y** | **Y** | **Y** | **Y** | **Y** | **Y** | **7** | **High** |
| Carvalho-Filho, 2008 | Aspectos epidemiológicos e soroprevalência da Leishmaniose e Babesiose em cães de raça no Município de São Luís (TESE) | 2013 | **Y** | **N** | **Y** | **Y** | **N** | **N** | **Y** | **N** | **4** | **Moderate** |
| Andreotti et al., 2006 | Occurrence of Neospora caninum in dogs and its correlation with visceral leishmaniasis in the urban area of Campo Grande, Mato Grosso do Sul, Brazil | 2013 | **Y** | **U** | **Y** | **Y** | **N** | **N** | **Y** | **N** | **4** | **Moderate** |
| Amóra et al., 2006 | Fatores relacionados com a positividade de cães para leishmaniose visceral em área endêmica do Estado do Rio  Grande do Norte, Brasil | 2013 | **Y** | **Y** | **Y** | **Y** | **N** | **N** | **Y** | **N** | **5** | **Moderate** |
| Souza et al., 2006 | Estudo epidemiológico de um surto de leishmaniose visceral numa área de manguezal | 2013 | **N** | **U** | **Y** | **Y** | **Y** | **Y** | **Y** | **Y** | **6** | **Moderate** |
| Santos et al., 2008 | Associations among immunological, parasitological and clinical  parameters in canine visceral leishmaniasis: Emaciation, spleen  parasitism, specific antibodies and leishmanin skin test reaction | 2013 | **Y** | **U** | **Y** | **Y** | **N** | **N** | **Y** | **N** | **4** | **Moderate** |
| Coura-Vital et al., 2011 | Prevalence and Factors Associated with  Leishmania  infantum Infection of Dogs from an Urban Area of Brazil  as Identified by Molecular Methods | 2013 | **Y** | **Y** | **Y** | **Y** | **Y** | **Y** | **Y** | **Y** | **8** | **High** |
| Rondon et al., 2008 | Cross-sectional serological study of canine Leishmania infection in Fortaleza, Ceará State, Brazil | 2013 | **Y** | **Y** | **Y** | **Y** | **N** | **N** | **Y** | **N** | **5** | **Moderate** |
| Antônio, 2009 | Prevalência da Leishmaniose Visceral Canina, Terra Indígena Krenak, Resplendor, Minas Gerais, Brasil, 2007 | 2013 | **Y** | **Y** | **Y** | **Y** | **N** | **N** | **Y** | **N** | **5** | **Moderate** |
| Lima, 2010 | Aspectos ambientais e sociais envolvidos na transmissão da L. Chagasi no Município de Parnamirim/RN (TESE) | 2013 | **Y** | **Y** | **Y** | **Y** | **N** | **N** | **N** | **N** | **4** | **Moderate** |
| Paranhos-Silva et al., 1996 | A cross-sectional serodiagnostic survey of canine leishmaniasis due to Leishmania chagasi | 2013 | **Y** | **Y** | **Y** | **Y** | **N** | **N** | **Y** | **N** | **5** | **Moderate** |
| Silva, 2007 | Inquérito Sorológico de Leishmaniose Canina na Cidade  de Rio Verde – GO (TESE) | 2013 | **Y** | **Y** | **Y** | **Y** | **N** | **N** | **Y** | **N** | **5** | **Moderate** |

**Legend: Y= Yes; N= No; U= Unclear; NA= Not applicable; Q1.** Were the criteria for inclusion in the sample clearly defined? **Q2.** Were the study subjects and the setting described in detail? **Q3.** Was the exposure measured in a valid and reliable way? **Q4.** Were objective, standard criteria used for measurement of the condition? **Q5.** Were confounding factors identified? **Q6.** Were strategies to deal with confounding factors stated? **Q7.** Were the outcomes measured in a valid and reliable way? **Q8.** Was appropriate statistical analysis used?

**Quality:**

“Low”: up to three “Yes” responses

“Moderate”: four to six “Yes”

“High”: seven or eight “Yes” responses
