# Supplementary material for: Risk and protective factors for canine visceral leishmaniasis in the Americas: a systematic review update with meta-analysis
Source: Parasit Vectors. 2026 Mar 18;19:185. doi: 10.1186/s13071-026-07325-0 (PMC13122873; doi:10.1186/s13071-026-07325-0)
Supplement: Supplementary file 3 — Additional file 3. Results of meta-analysis: assessment of heterogeneities with description of Q test, P-values and I2 statistics. [file 13071_2026_7325_MOESM3_ESM.docx]

**Additional file 3 – Results of meta-analysis: assessment of heterogeneities with description of Q test, *P*-values ​​and I² measures**

| **Variable/moderators** | **Q-value** | ***P*-value** | **I-squared (I²)** |
| --- | --- | --- | --- |
| **Sex** | 162,638 | <0,001 | 55,115 |
| Confounding control/No | 117,328 | <0,001 | 50,566 |
| Confounding control/Yes | 40,089 | <0,001 | 70,066 |
| Confounding control/Total within | 157,417 | <0,001 | **-** |
| Confounding control/Total between | 0,976 | 0,323 | **-** |
| **Age (≤1 year vs >1 year)** | 283,266 | <0,001 | 88,350 |
| **Age (≤2 years vs >2 years)** | 85,419 | <0,001 | 82,440 |
| Total within | 368,686 | <0,001 | - |
| Total between | 5,183 | 0,023 | - |
| Confounding control/No | 335,058 | <0,001 | 87,763 |
| Confounding control/Yes | 32,825 | <0,001 | 87,814 |
| Confounding control/Total within | 367,883 | <0,001 | **-** |
| Confounding control/Total between | 2,605 | 0,107 | **-** |
| **Breed** | 89,112 | <0,001 | 56,235 |
| Confounding control/No | 67,669 | <0,001 | 52,711 |
| Confounding control/Yes | 12,193 | 0,058 | 50,791 |
| Confounding control/Total within | 79,862 | <0,001 | **-** |
| Confounding control/Total between | 9,250 | 0,002 | **-** |
| **Length of hair** | 158,275 | <0,001 | 77,255 |
| **Presence of chickens/chicken coop** | 108,811 | <0,001 | 75,186 |
| **Existence of a yard adjacent to the home** | 23,267 | 0,001 | 74,213 |
| **Dog stays predominantly in the yard** | 11,260 | 0,024 | 64,476 |
| **Presence of organic matter in the yard** | 22,457 | 0,004 | 64,377 |
| **Backyard floor** | 10,035 | 0,040 | 60,139 |
| **Presence of vacant land** | 1,074 | 0,898 | 0,000 |
| **Presence of vegetation** | 177,835 | <0,001 | 88,191 |
| **Presence of ectoparasites** | 34,687 | 0,003 | 56,757 |
| **Access to the streets** | 86,956 | <0,001 | 67,800 |
| **Dog breeding place** | 54,927 | 0,001 | 54,485 |
| **Presence of other dogs** | 57,352 | <0,001 | 73,846 |
| Type of study/Case control | 0,000 | 1,000 | 0,000 |
| Type of study/Cohort | 4,546 | 0,033 | 78,000 |
| Type of study/Sectional | 26,036 | 0,011 | 53,910 |
| Total within | 30,582 | 0,004 | **-** |
| Total between | 26,770 | <0,001 | **-** |
| **Presence of horses** | 22,586 | 0,031 | 46,870 |
| Confounding control/No | 8,281 | 0,308 | 15,468 |
| Confounding control/Yes | 3,896 | 0,420 | 0,000 |
| Confounding control/Total within | 12,177 | 0,350 | **-** |
| Confounding control/Total between | 10,409 | 0,001 | **-** |
| **Contact with small rodents** | 19,904 | 0,019 | 54,783 |
| **Presence of cats** | 50,138 | <0,001 | 70,083 |
| **Contact with pigs/pigpens** | 32,321 | 0,001 | 65,966 |
| **Size (small vs medium)** | 54,487 | <0,001 | 74,306 |
| **Size (small vs large)** | 65,229 | <0,001 | 73,938 |
| **Hair color** | 23,208 | 0,006 | 61,221 |
| **Level of education** | 40,810 | <0,001 | 75,496 |
| **Income (1 minimum wages vs > 1 minimum wages)** | 46,692 | <0,001 | 91,433 |
| **Income (≤2 minimum wages vs > 2 minimum wages)** | 141,712 | <0,001 | 96,472 |
| **Type of water for consumption** | 0,970 | 0,914 | 0,000 |
| **Garbage colletion** | 19,070 | 0,014 | 58,048 |
| **Sewage colletion** | 11,808 | 0,008 | 74,594 |
| **Sleeping place (peri-domicile vs inside the house)** | 18,805 | 0,027 | 52,141 |
| **Sleeping place (outdoors vs indoors)** | 2,463 | 0,782 | 0,000 |
| **How the dog spends the night (loose vs tied)** | 8,655 | 0,070 | 53,785 |
| **Watchdog** | 2,710 | 0,438 | 0,000 |
| **Dog acquisition (from the streets vs others)** | 7,909 | 0,245 | 24,139 |
| **Hunting dog** | 8,911 | 0,113 | 43,890 |
| **Neutered dog** | 7,402 | 0,116 | 45,964 |
| **Dog food (commercial vs home prepared or commercial)** | 21,585 | 0,0003 | 67,569 |
| **Dewormed dog** | 4,900 | 0,298 | 18,368 |
| **Regular veterinary evaluation** | 20,338 | 0,005 | 65,582 |
| **Understanding of the disease** | 32,682 | <0,001 | 72,462 |
| **Vector seen in the house** | 1,554 | 0,670 | 0,000 |
| **Previous case** | 20,294 | 0,005 | 65,507 |
| **Presence of neighbors with CVL** | 10,471 | 0,033 | 61,798 |
| **Previous occurrence of HVL** | 3,735 | 0,443 | 0,000 |
