# Supplementary material for: Risk and protective factors for canine visceral leishmaniasis in the Americas: a systematic review update with meta-analysis
Source: Parasit Vectors. 2026 Mar 18;19:185. doi: 10.1186/s13071-026-07325-0 (PMC13122873; doi:10.1186/s13071-026-07325-0)
Supplement: Supplementary file 4 — Additional file 4. GRADE Summary of Findings table for the main exposures associated with canine visceral leishmaniasis. [file 13071_2026_7325_MOESM4_ESM.docx]

# Additional file 4: GRADE Summary of Findings table for the main exposures associated with canine visceral leishmaniasis

| Exposure | Outcome | Odds Ratio (95% Confidence interval)* | Number of studies** | Number of dogs | Certainty of evidence (GRADE) |
| --- | --- | --- | --- | --- | --- |
| Length of dog hair  (short *vs.* long) | Canine visceral leishmaniasis (CVL) | 1.44 (1.24 – 1.68) | 12 | 77,107 | ⊕⊕⊕⊝ Moderate |
| Access to the streets  (yes vs no) | Canine visceral leishmaniasis (CVL) | 1.76 (1.14 – 2.70) | 8 | 4,470 | ⊕⊕⊕⊝ Moderate |
| Presence of vegetation near the domicile or in the adjacent environment (yes vs no) | Canine visceral leishmaniasis (CVL) | 1.70 (1.06 –2.72) | 10 | 6,610 | ⊕⊕⊕⊝ Moderate |
| Dog’s dwelling area: (intradomicile vs peridomicile) | Canine visceral leishmaniasis (CVL) | 0.51 (0.56 – 0.72) | 7 | 5,417 | ⊕⊕⊕⊝ Moderate |

* Effect estimates refer to pooled results from studies that controlled for confounding factors.

** The number of studies refers to those that controlled for confounding factors and contributed to the pooled estimates.
