# Supplementary material for: Risk and protective factors for canine visceral leishmaniasis in the Americas: a systematic review update with meta-analysis
Source: Parasit Vectors. 2026 Mar 18;19:185. doi: 10.1186/s13071-026-07325-0 (PMC13122873; doi:10.1186/s13071-026-07325-0)
Supplement: Supplementary file 5 — Additional file 5. Forest plots of analyzed variables (Figs. S1–S22). [file 13071_2026_7325_MOESM5_ESM.docx]

**Additional file 5: Forest plots of analyzed variables**

*Notes:*

*-Numerical values in the figures are presented with decimal commas due to software formatting and could not be modified*

*-The forest plots include studies from both the 2013 review (search completed up to September 2011) and the current (present) review (studies published from October 2011 up to June 2024).*


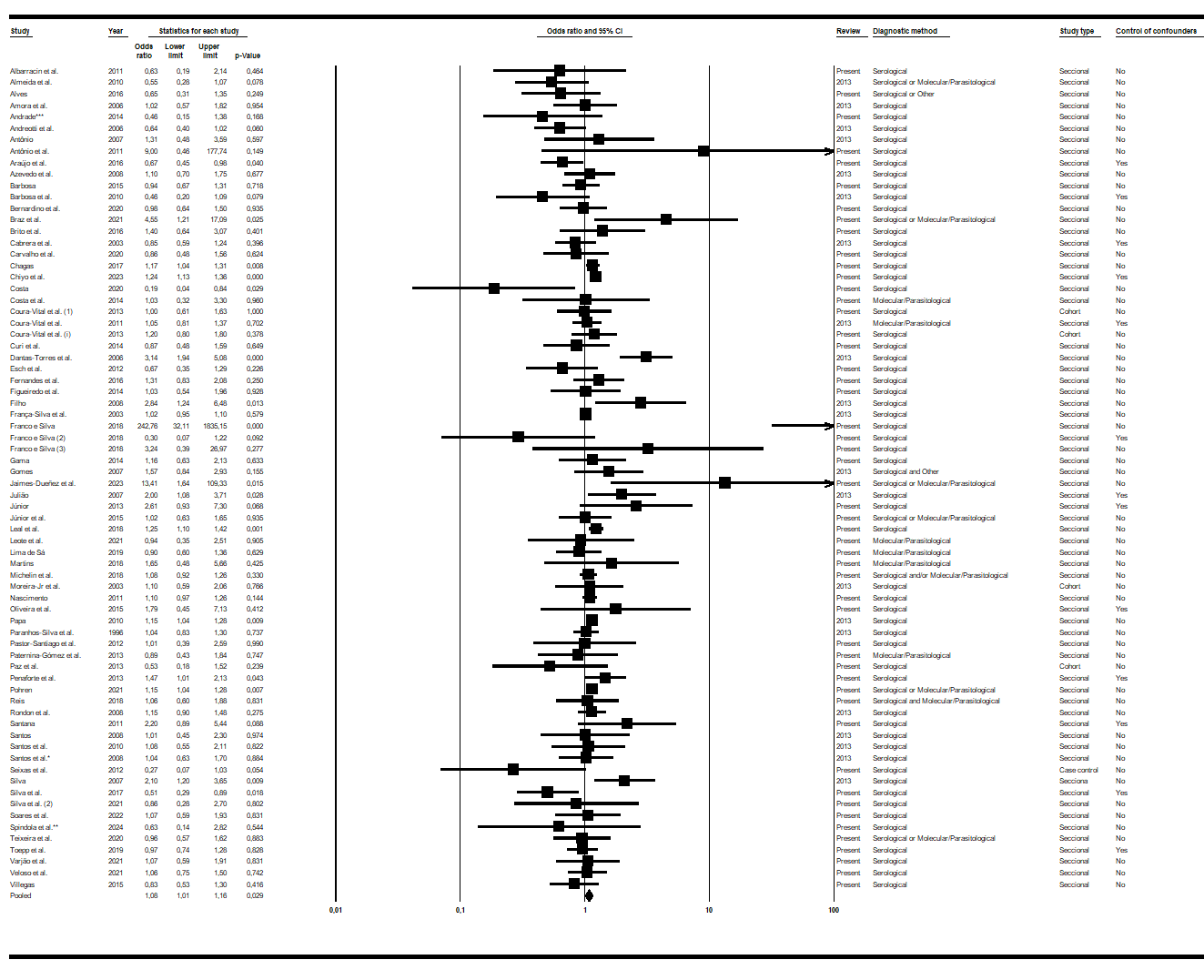


**Fig. S1.** Forest plot of the association between dog sex and canine visceral leishmaniasis. Superscripts indicate: * result of a serological test in a study involving two diagnostic tests; ** result of a second serological test; *** result of a third serological test; (1) different studies by the same author in the same year; (2) second result reported in a single publication; (3) third result reported in a single publication; (i) second result from the same study; (ii) third result from the same study. Squares represent the weight of each study, whereas diamonds represent the pooled estimate for each subgroup. **Reference category:** female (odds ratio = 1).


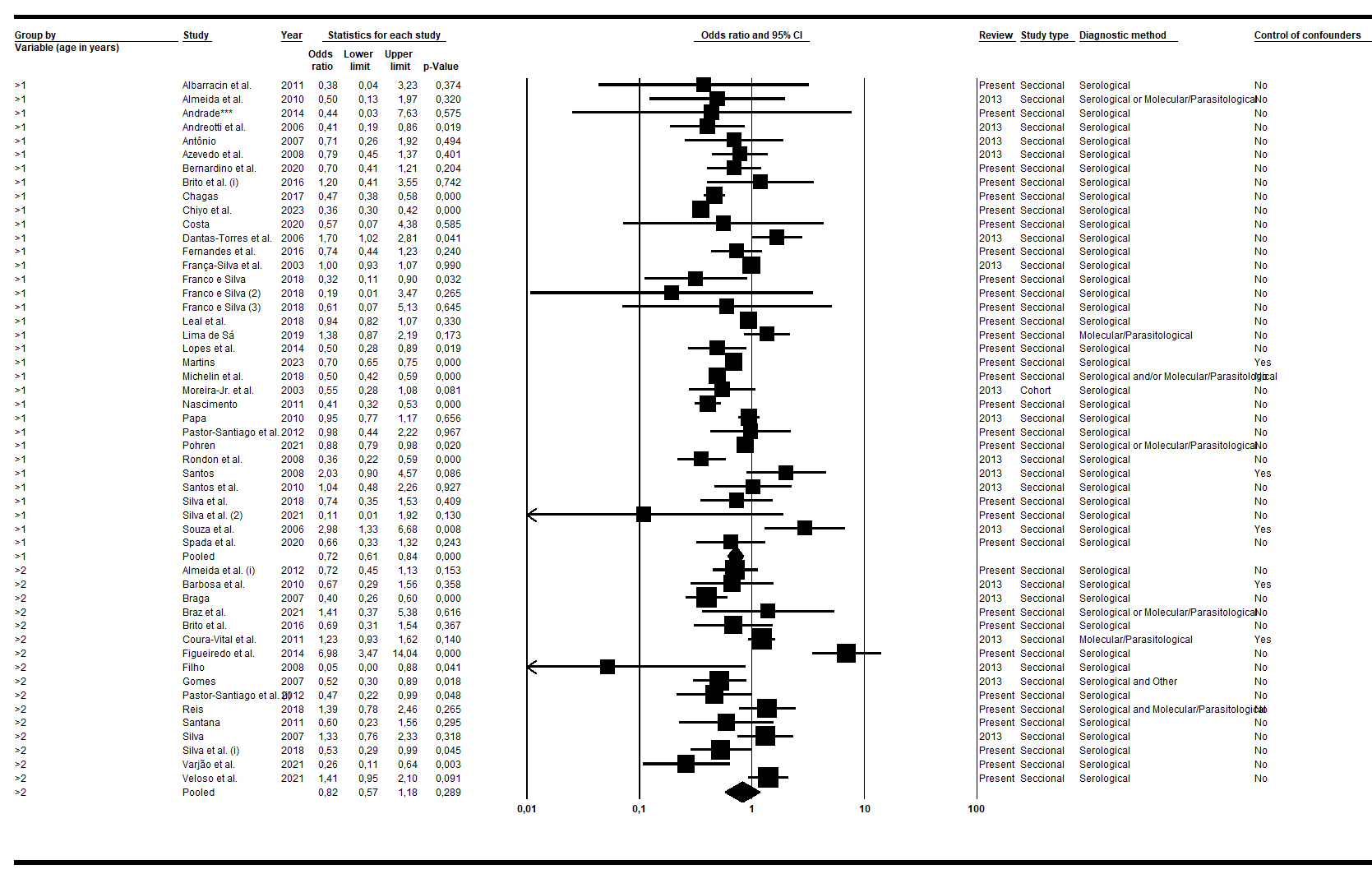


**Fig. S2.** Forest plot of the association between dog age and canine visceral leishmaniasis. Superscripts indicate: * result of a serological test in a study involving two diagnostic tests; ** result of a second serological test; *** result of a third serological test; (1) different studies by the same author in the same year; (2) second result reported in a single publication; (3) third result reported in a single publication; (i) second result from the same study; (ii) third result from the same study. Squares represent the weight of each study, whereas diamonds represent the pooled estimate for each subgroup. **Reference category:** old age (odds ratio = 1).


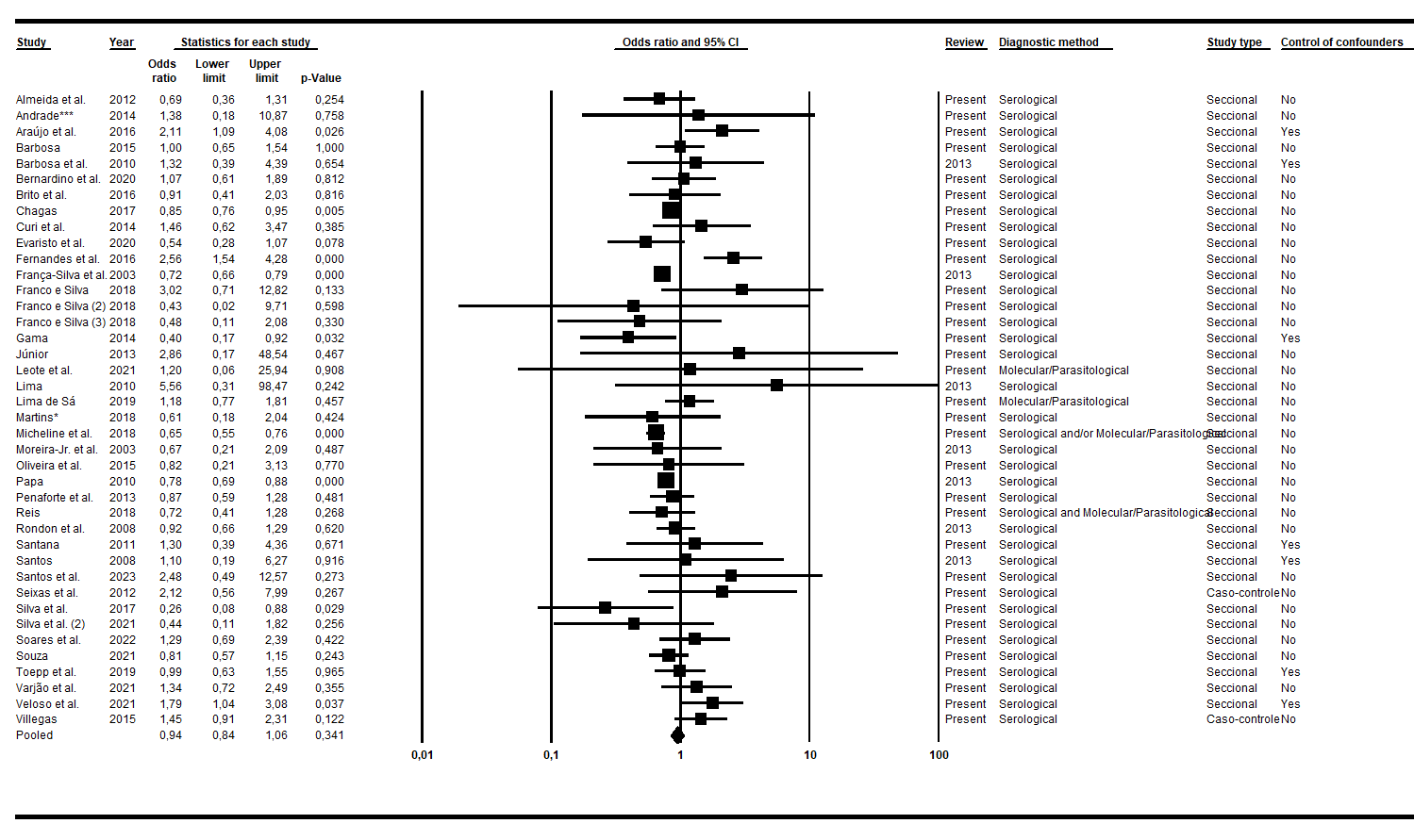


**Fig. S3.** Forest plot of the association between dog breed and canine visceral leishmaniasis. Superscripts indicate: * result of a serological test in a study involving two diagnostic tests; ** result of a second serological test; *** result of a third serological test; (1) different studies by the same author in the same year; (2) second result reported in a single publication; (3) third result reported in a single publication; (i) second result from the same study; (ii) third result from the same study. Squares represent the weight of each study, whereas diamonds represent the pooled estimate for each subgroup. **Reference category:** purebred (odds ratio = 1).


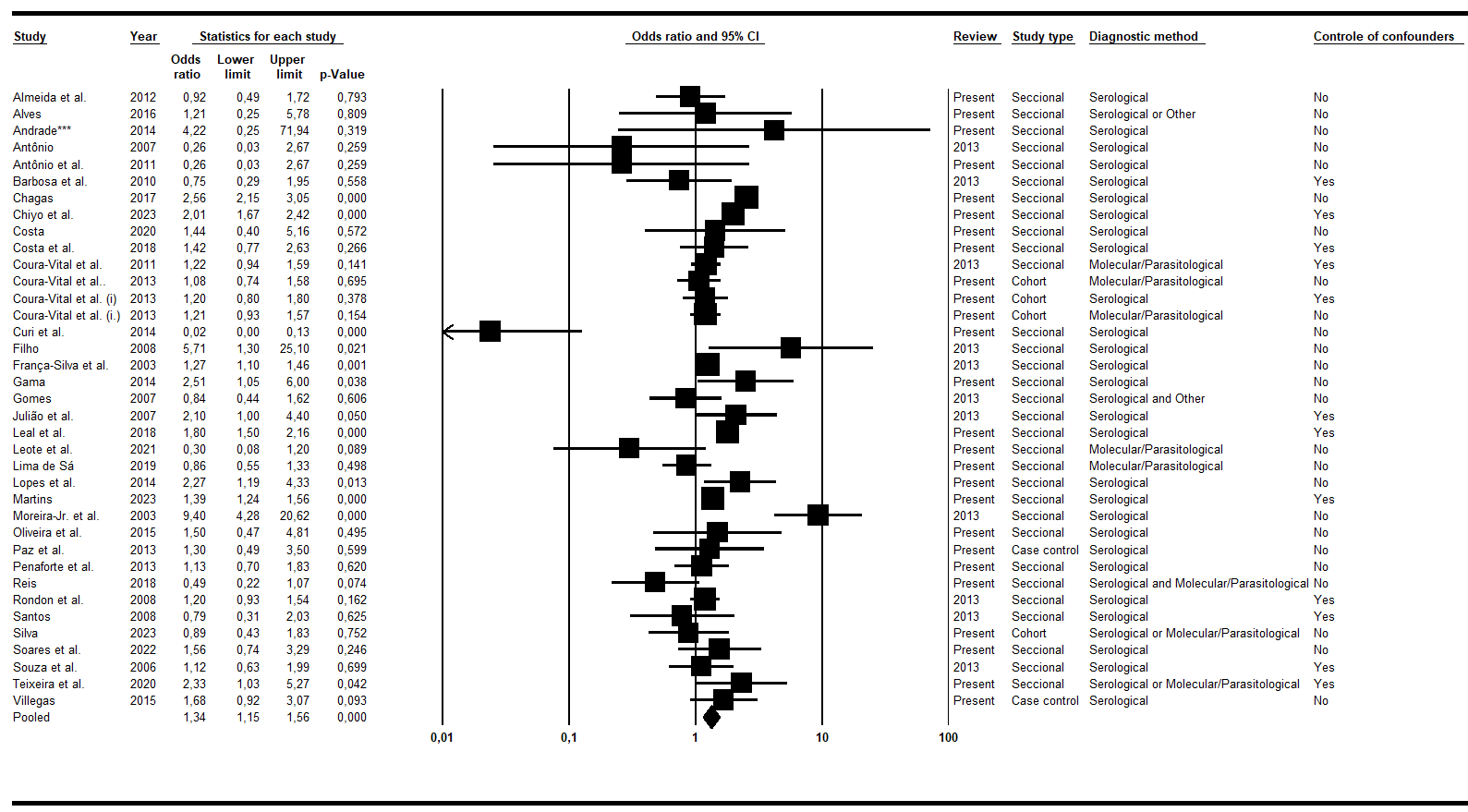


**Fig. S4.** Forest plot of the association between hair length and canine visceral leishmaniasis. Superscripts indicate: * result of a serological test in a study involving two diagnostic tests; ** result of a second serological test; *** result of a third serological test; (1) different studies by the same author in the same year; (2) second result reported in a single publication; (3) third result reported in a single publication; (i) second result from the same study; (ii) third result from the same study. Squares represent the weight of each study, whereas diamonds represent the pooled estimate for each subgroup. **Reference category:** long hair (odds ratio = 1)..


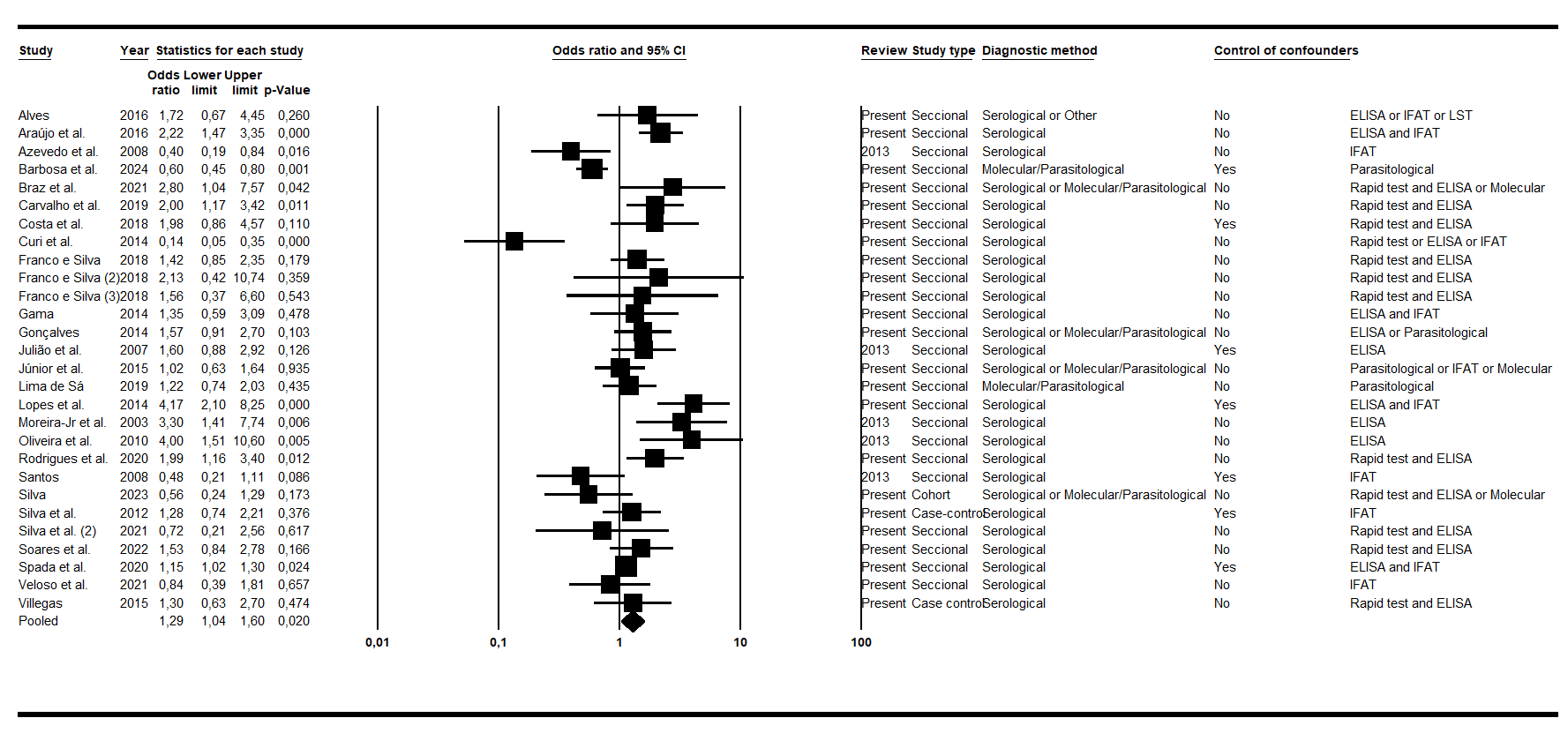


**Fig. S5.** Forest plot of the association between the presence of chickens and/or a chicken coop at the household and canine visceral leishmaniasis. Superscripts indicate: * result of a serological test in a study involving two diagnostic tests; ** result of a second serological test; *** result of a third serological test; (1) different studies by the same author in the same year; (2) second result reported in a single publication; (3) third result reported in a single publication; (i) second result from the same study; (ii) third result from the same study. Squares represent the weight of each study, whereas diamonds represent the pooled estimate for each subgroup. **Reference category:** no (odds ratio = 1).


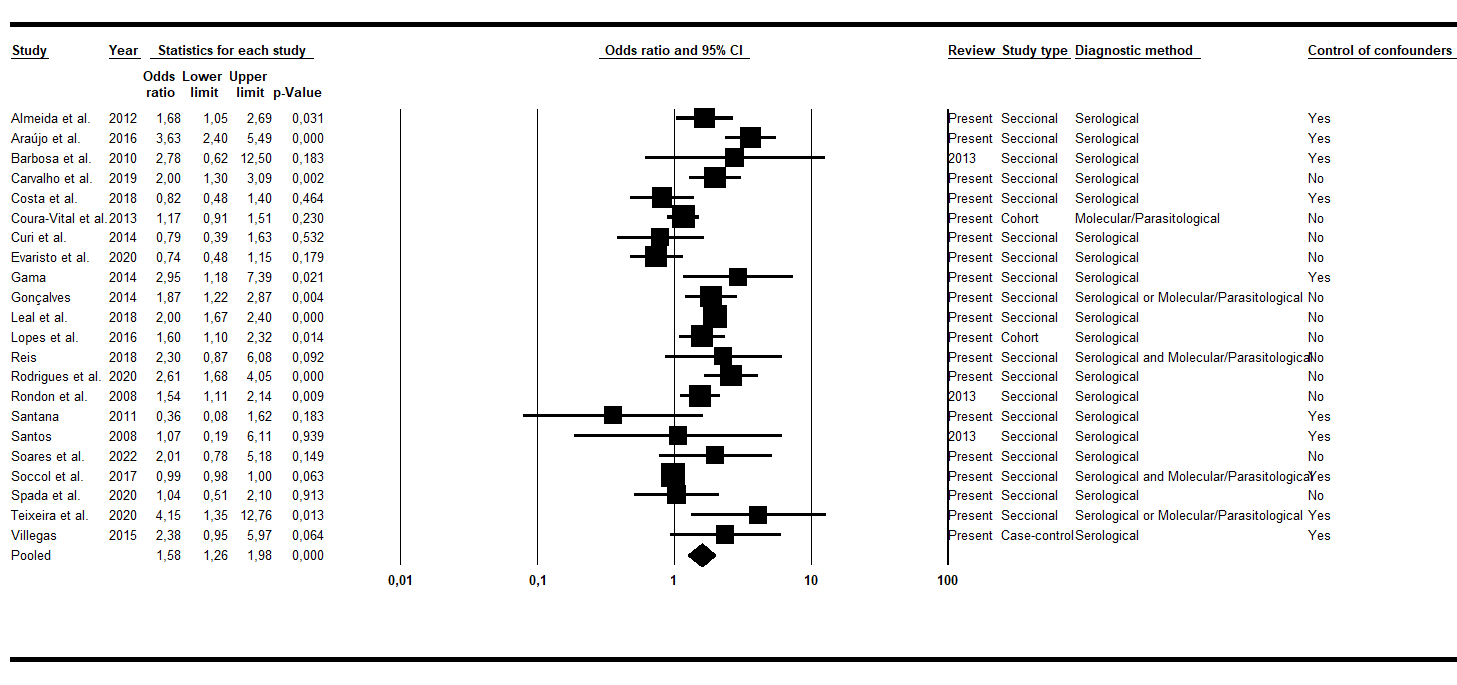


**Fig. S6.** Forest plot of the association between the presence of vegetation and canine visceral leishmaniasis. Superscripts indicate: * result of a serological test in a study involving two diagnostic tests; ** result of a second serological test; *** result of a third serological test; (1) different studies by the same author in the same year; (2) second result reported in a single publication; (3) third result reported in a single publication; (i) second result from the same study; (ii) third result from the same study. Squares represent the weight of each study, whereas diamonds represent the pooled estimate for each subgroup. **Reference category:** no (odds ratio = 1).


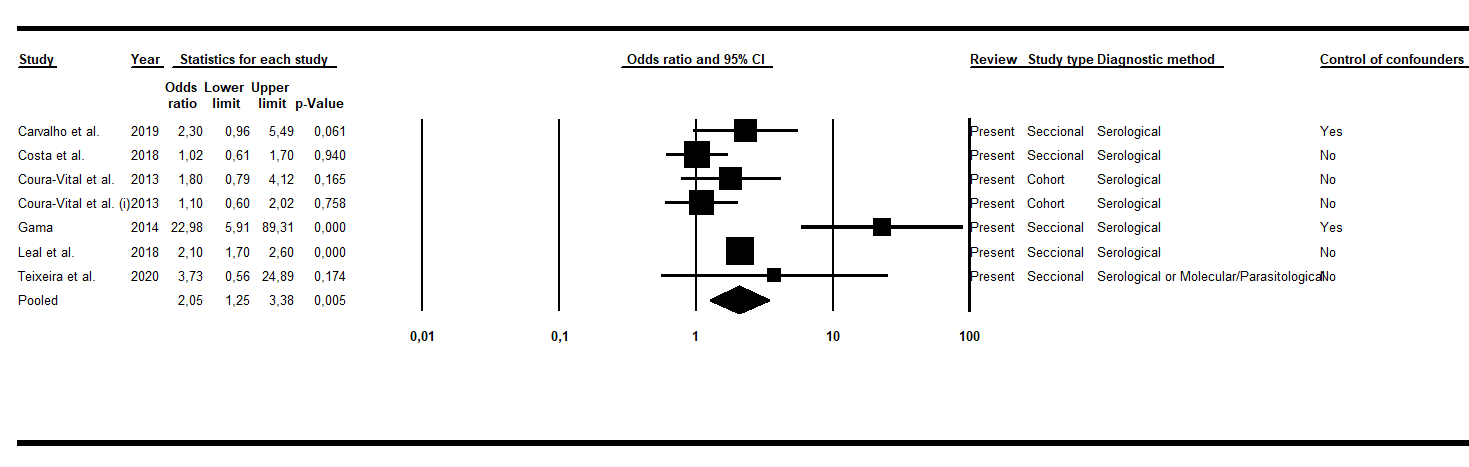


**Fig. S7.** Forest plot of the association between the existence of a yard adjacent to the home and canine visceral leishmaniasis. Superscripts indicate: * result of a serological test in a study involving two diagnostic tests; ** result of a second serological test; *** result of a third serological test; (1) different studies by the same author in the same year; (2) second result reported in a single publication; (3) third result reported in a single publication; (i) second result from the same study; (ii) third result from the same study. Squares represent the weight of each study, whereas diamonds represent the pooled estimate for each subgroup. **Reference category:** no (odds ratio = 1).


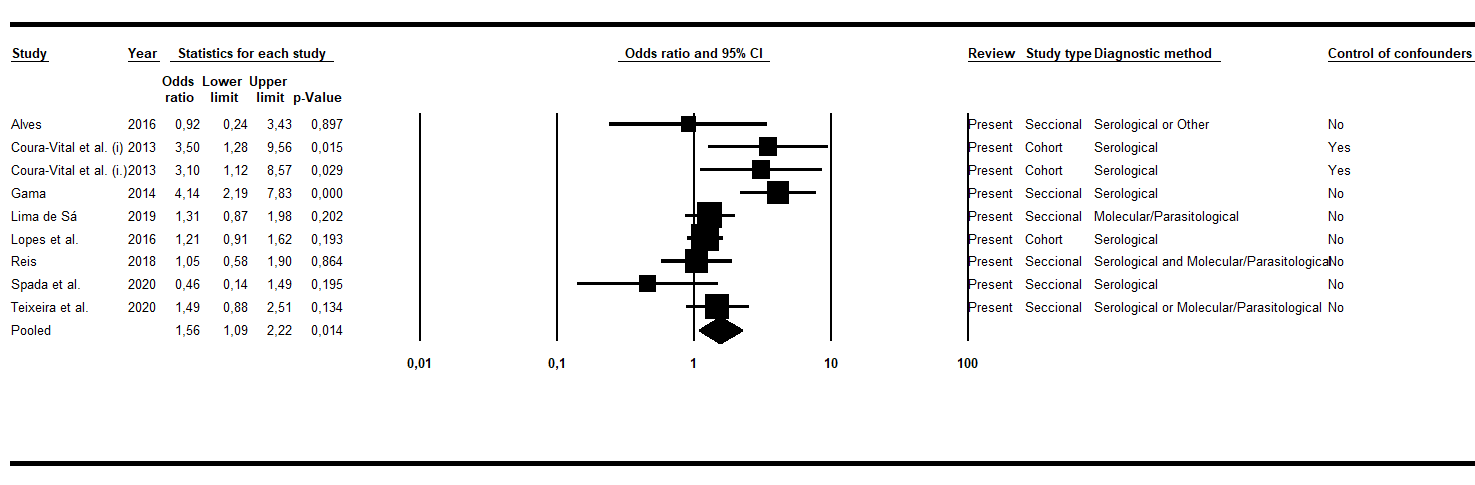


**Fig. S8.** Forest plot of the association between the presence of organic matter in the yard and canine visceral leishmaniasis. Superscripts indicate: * result of a serological test in a study involving two diagnostic tests; ** result of a second serological test; *** result of a third serological test; (1) different studies by the same author in the same year; (2) second result reported in a single publication; (3) third result reported in a single publication; (i) second result from the same study; (ii) third result from the same study. Squares represent the weight of each study, whereas diamonds represent the pooled estimate for each subgroup. **Reference category:** no (odds ratio = 1).


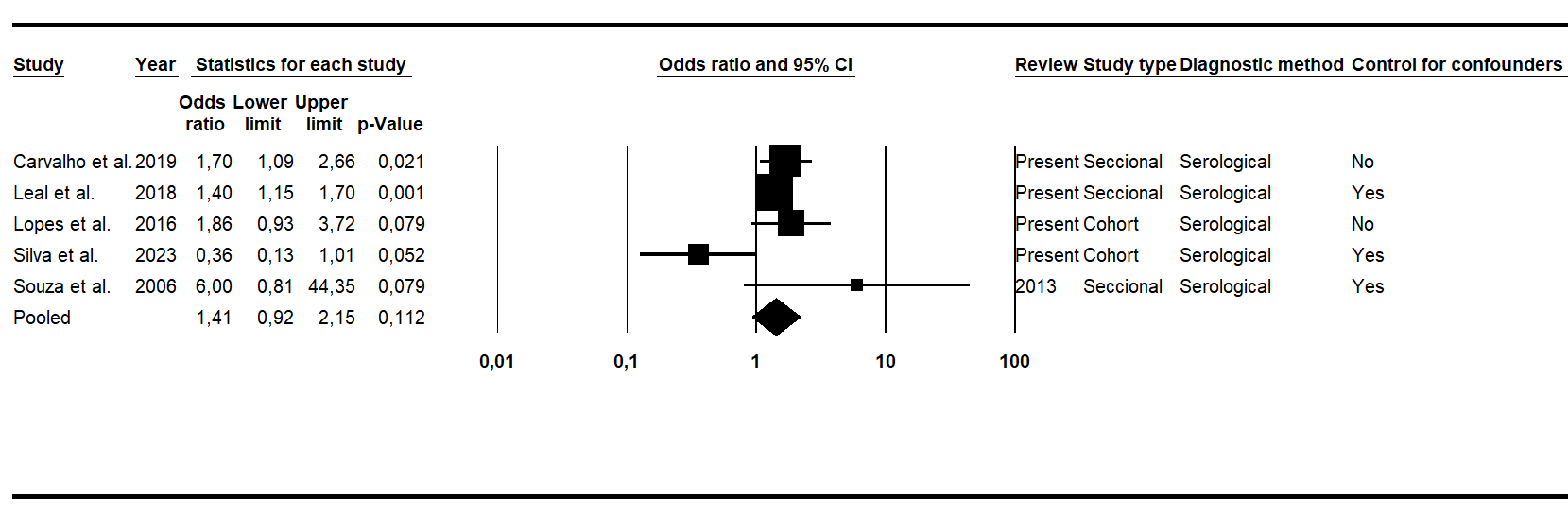


**Fig. S9.** Forest plot of the association between backyard floor type and canine visceral leishmaniasis. Superscripts indicate: * result of a serological test in a study involving two diagnostic tests; ** result of a second serological test; *** result of a third serological test; (1) different studies by the same author in the same year; (2) second result reported in a single publication; (3) third result reported in a single publication; (i) second result from the same study; (ii) third result from the same study. Squares represent the weight of each study, whereas diamonds represent the pooled estimate for each subgroup. **Reference category:** paved floor (odds ratio = 1).

**
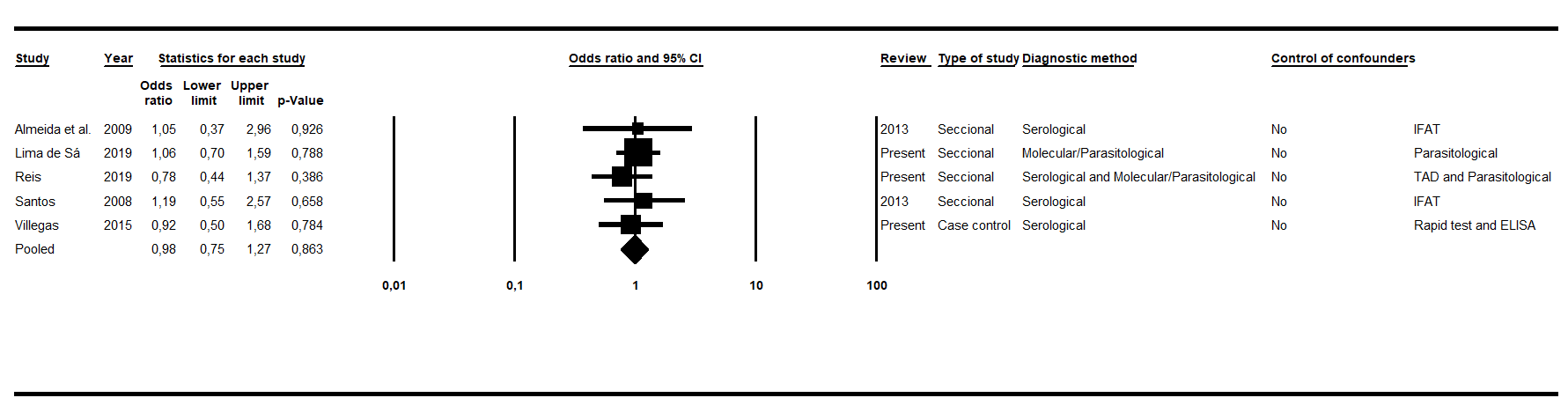
**

**Fig. S10.** Forest plot of the association between the presence of vacant land and canine visceral leishmaniasis. Superscripts indicate: * result of a serological test in a study involving two diagnostic tests; ** result of a second serological test; *** result of a third serological test; (1) different studies by the same author in the same year; (2) second result reported in a single publication; (3) third result reported in a single publication; (i) second result from the same study; (ii) third result from the same study. Squares represent the weight of each study, whereas diamonds represent the pooled estimate for each subgroup. **Reference category:** no (odds ratio = 1).


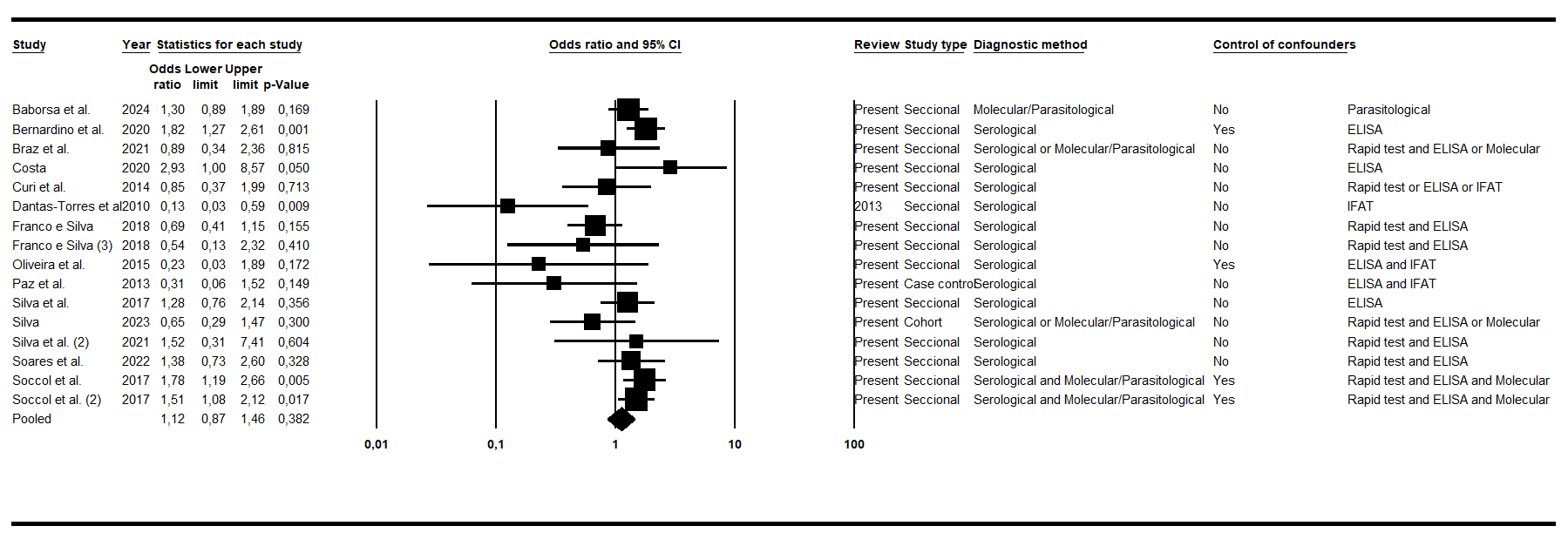


**Fig. S11.** Forest plot of the association between the presence of ectoparasites and canine visceral leishmaniasis. Superscripts indicate: * result of a serological test in a study involving two diagnostic tests; ** result of a second serological test; *** result of a third serological test; (1) different studies by the same author in the same year; (2) second result reported in a single publication; (3) third result reported in a single publication; (i) second result from the same study; (ii) third result from the same study. Squares represent the weight of each study, whereas diamonds represent the pooled estimate for each subgroup. **Reference category:** no (odds ratio = 1).


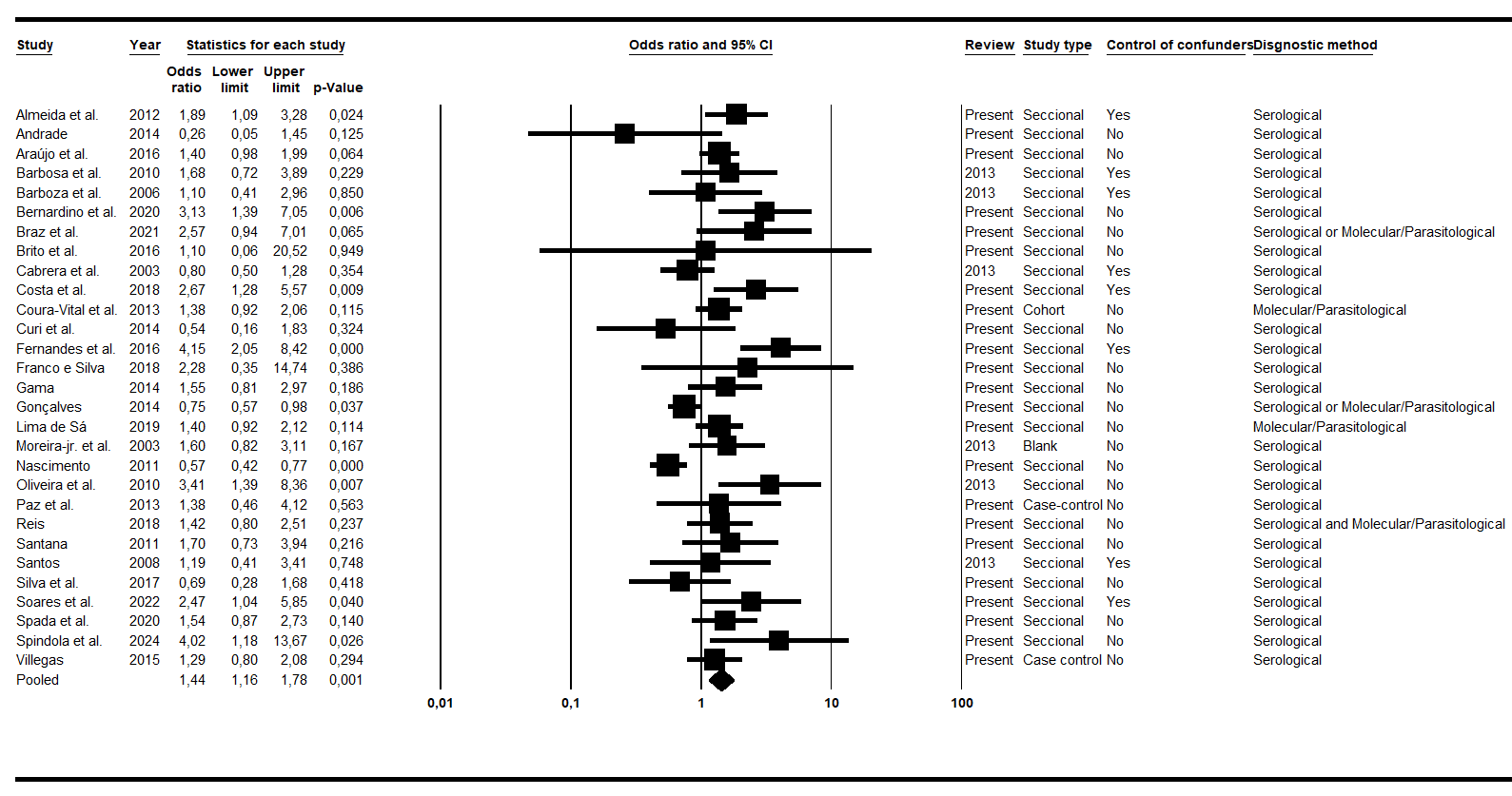


**Fig. S12.** Forest plot of the association between street access and canine visceral leishmaniasis. Superscripts indicate: * result of a serological test in a study involving two diagnostic tests; ** result of a second serological test; *** result of a third serological test; (1) different studies by the same author in the same year; (2) second result reported in a single publication; (3) third result reported in a single publication; (i) second result from the same study; (ii) third result from the same study. Squares represent the weight of each study, whereas diamonds represent the pooled estimate for each subgroup. **Reference category:** no (odds ratio = 1).


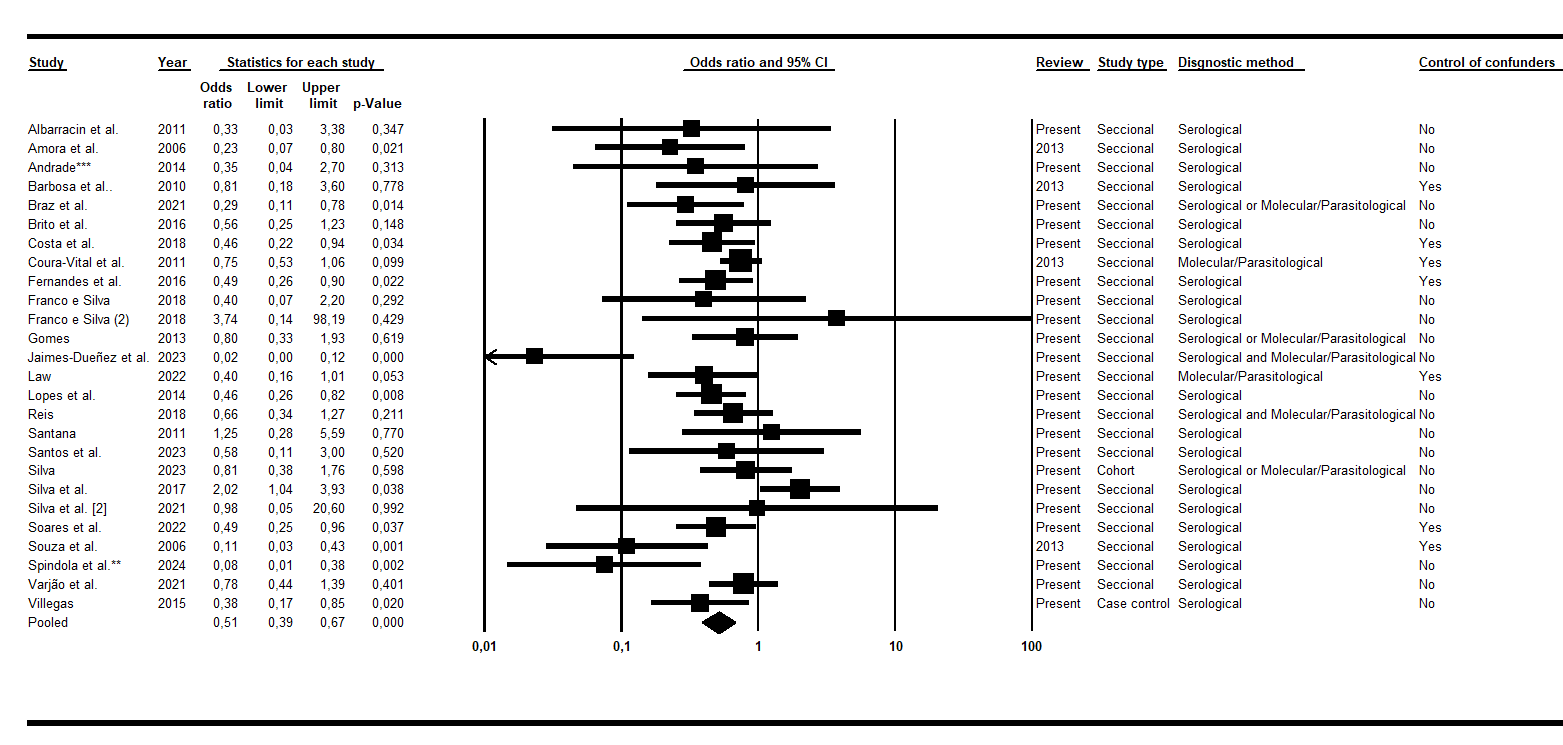


**Fig. S13.** Forest plot of the association between dog dwelling area and canine visceral leishmaniasis. Superscripts indicate: * result of a serological test in a study involving two diagnostic tests; ** result of a second serological test; *** result of a third serological test; (1) different studies by the same author in the same year; (2) second result reported in a single publication; (3) third result reported in a single publication; (i) second result from the same study; (ii) third result from the same study. Squares represent the weight of each study, whereas diamonds represent the pooled estimate for each subgroup. **Reference category:** peridomiciliary (odds ratio = 1).


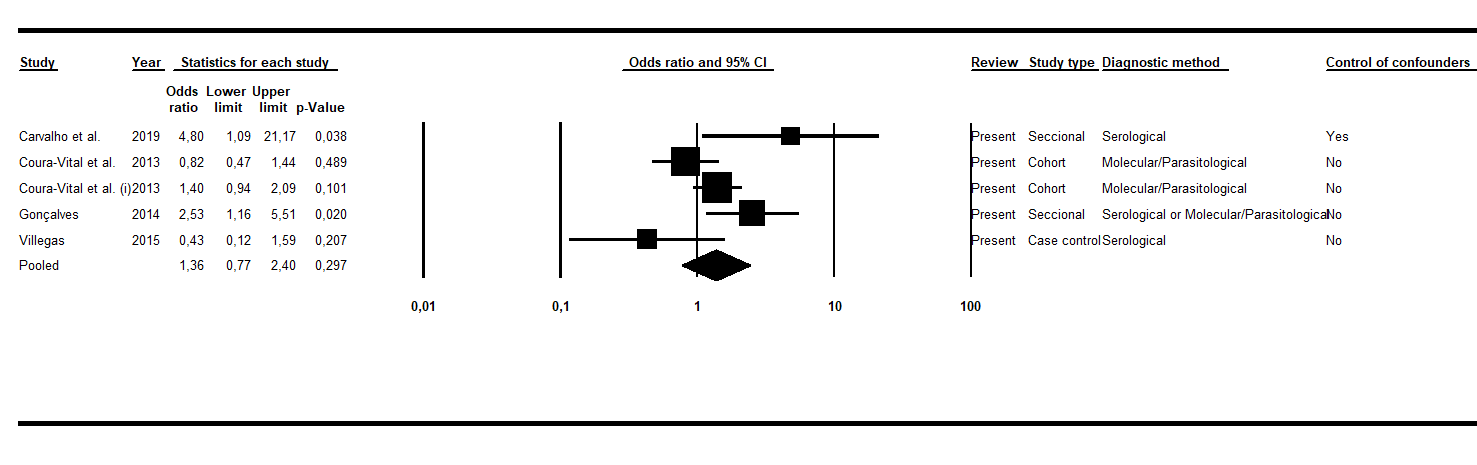


**Fig. S14.** Forest plot of the association between dogs predominantly staying in the yard and canine visceral leishmaniasis. Superscripts indicate: * result of a serological test in a study involving two diagnostic tests; ** result of a second serological test; *** result of a third serological test; (1) different studies by the same author in the same year; (2) second result reported in a single publication; (3) third result reported in a single publication; (i) second result from the same study; (ii) third result from the same study. Squares represent the weight of each study, whereas diamonds represent the pooled estimate for each subgroup. **Reference category:** no (odds ratio = 1).


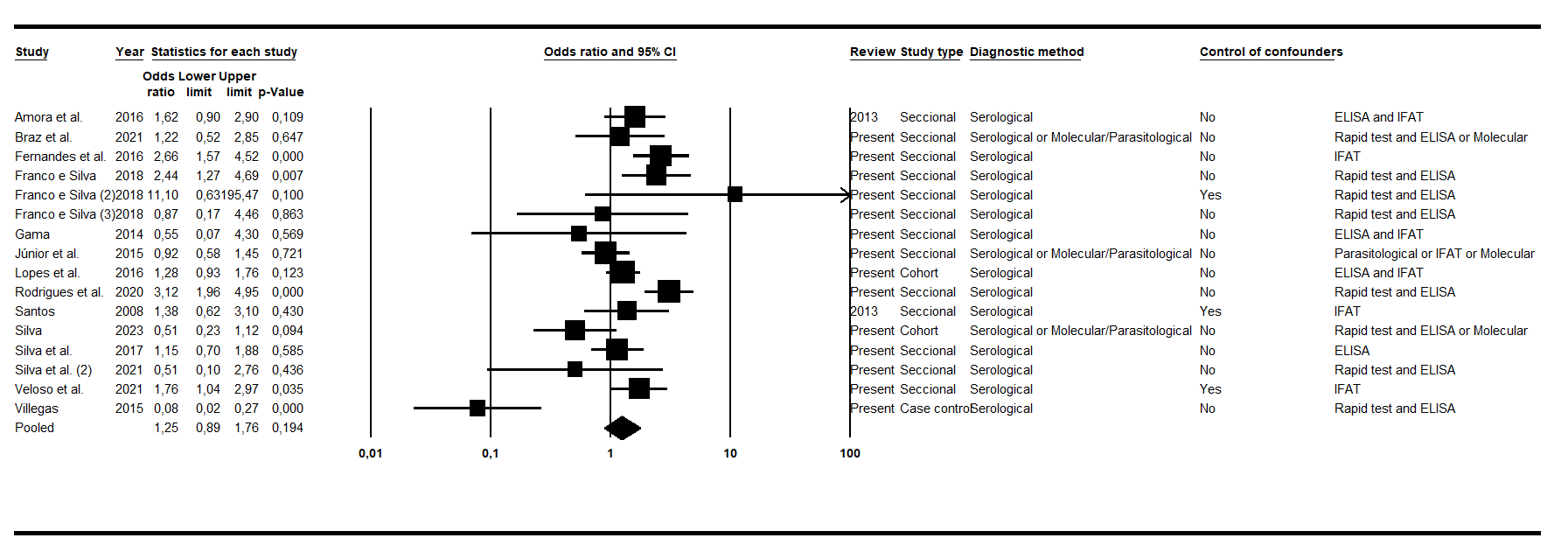


**Fig. S15.** Forest plot of the association between the presence of other dogs in the household and canine visceral leishmaniasis. Superscripts indicate: * result of a serological test in a study involving two diagnostic tests; ** result of a second serological test; *** result of a third serological test; (1) different studies by the same author in the same year; (2) second result reported in a single publication; (3) third result reported in a single publication; (i) second result from the same study; (ii) third result from the same study. Squares represent the weight of each study, whereas diamonds represent the pooled estimate for each subgroup. **Reference category:** no (odds ratio = 1).


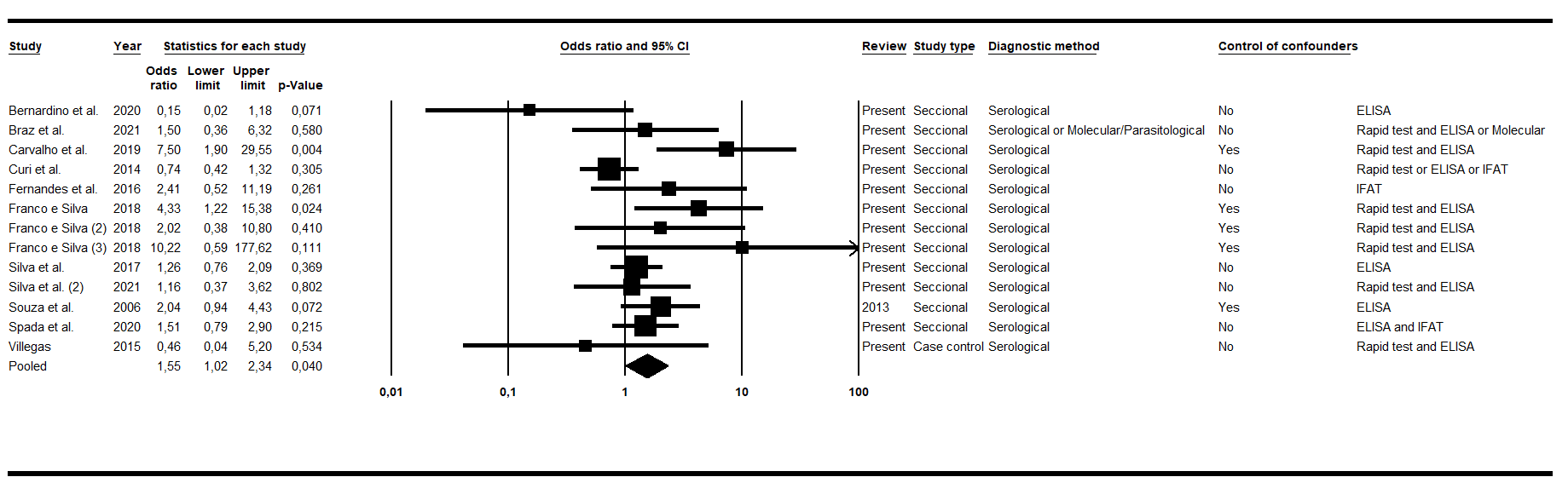


**Fig. S16.** Forest plot of the association between the presence of horses and canine visceral leishmaniasis. Superscripts indicate: * result of a serological test in a study involving two diagnostic tests; ** result of a second serological test; *** result of a third serological test; (1) different studies by the same author in the same year; (2) second result reported in a single publication; (3) third result reported in a single publication; (i) second result from the same study; (ii) third result from the same study. Squares represent the weight of each study, whereas diamonds represent the pooled estimate for each subgroup. **Reference category:** no (odds ratio = 1).

**
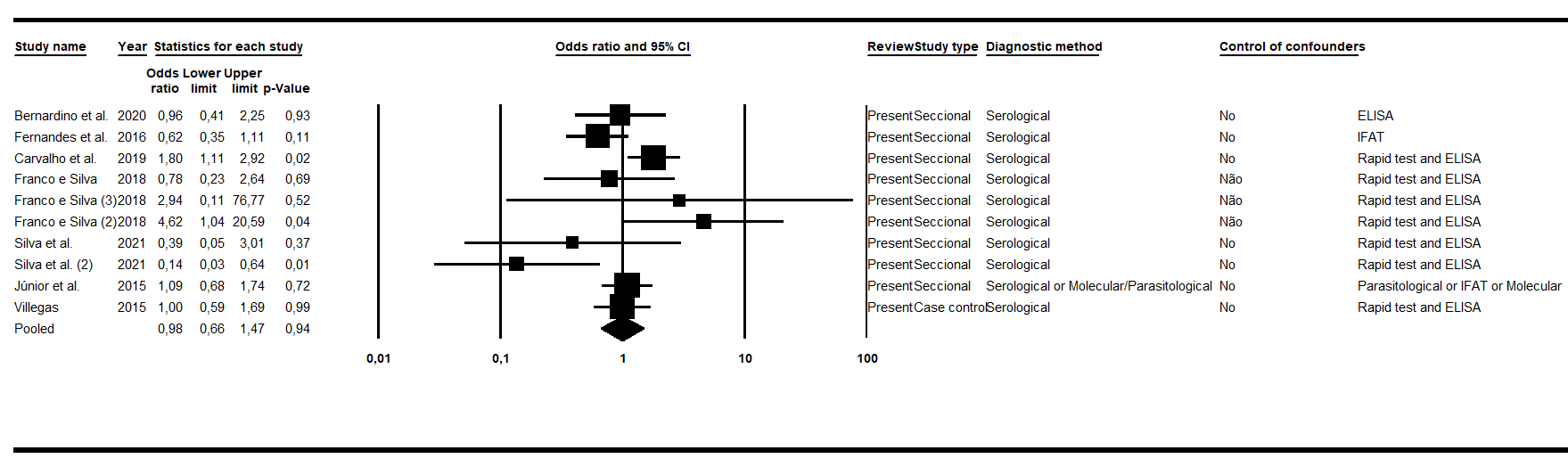
**

**Fig. S17.** Forest plot of the association between contact with small rodents and canine visceral leishmaniasis. Superscripts indicate: * result of a serological test in a study involving two diagnostic tests; ** result of a second serological test; *** result of a third serological test; (1) different studies by the same author in the same year; (2) second result reported in a single publication; (3) third result reported in a single publication; (i) second result from the same study; (ii) third result from the same study. Squares represent the weight of each study, whereas diamonds represent the pooled estimate for each subgroup. **Reference category:** no (odds ratio = 1).

**
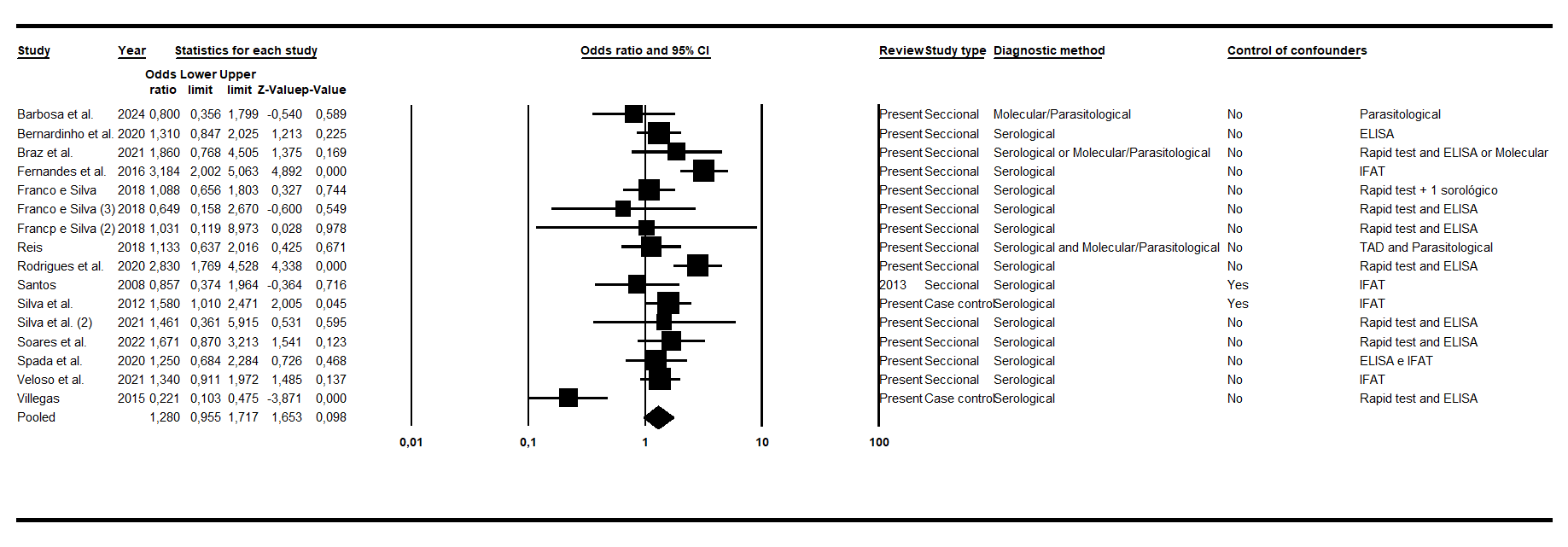
**

**Fig. S18.** Forest plot of the association between the presence of cats and canine visceral leishmaniasis. Superscripts indicate: * result of a serological test in a study involving two diagnostic tests; ** result of a second serological test; *** result of a third serological test; (1) different studies by the same author in the same year; (2) second result reported in a single publication; (3) third result reported in a single publication; (i) second result from the same study; (ii) third result from the same study. Squares represent the weight of each study, whereas diamonds represent the pooled estimate for each subgroup. **Reference category:** no (odds ratio = 1).

**
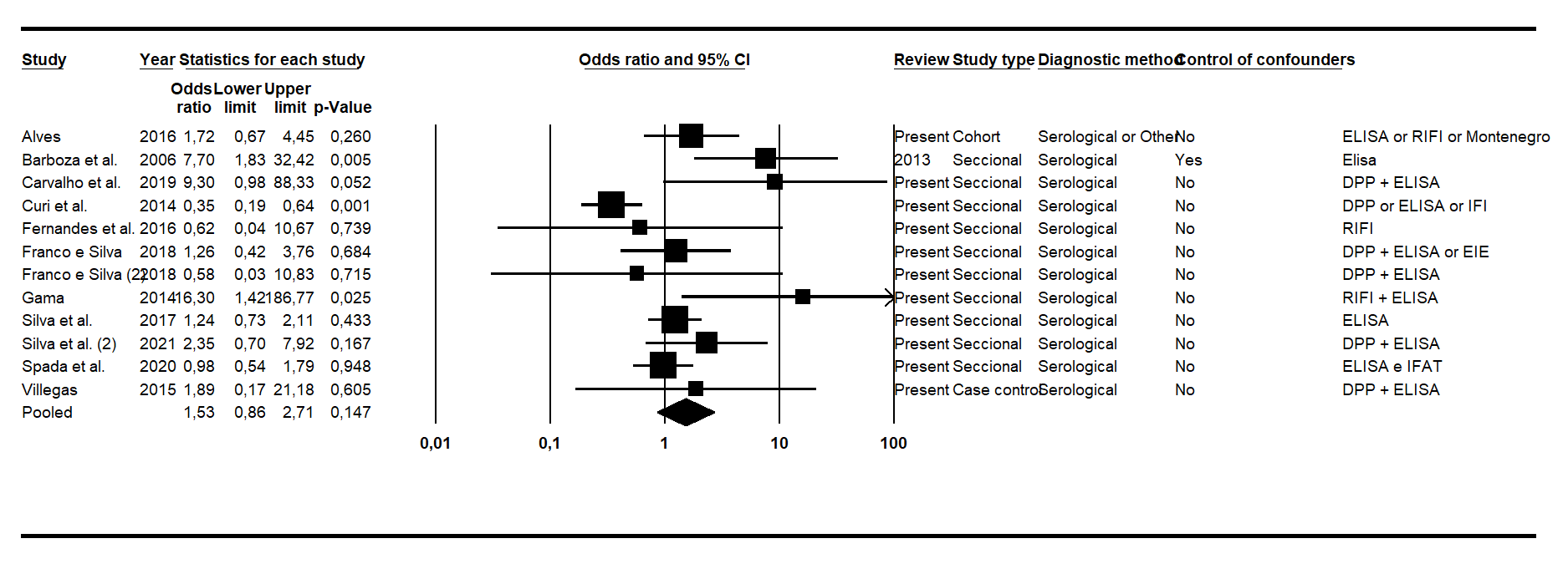
**

**Fig. S19.** Forest plot of the association between contact with pigs and/or pigpens and canine visceral leishmaniasis. Superscripts indicate: * result of a serological test in a study involving two diagnostic tests; ** result of a second serological test; *** result of a third serological test; (1) different studies by the same author in the same year; (2) second result reported in a single publication; (3) third result reported in a single publication; (i) second result from the same study; (ii) third result from the same study. Squares represent the weight of each study, whereas diamonds represent the pooled estimate for each subgroup. **Reference category:** no (odds ratio = 1).

**
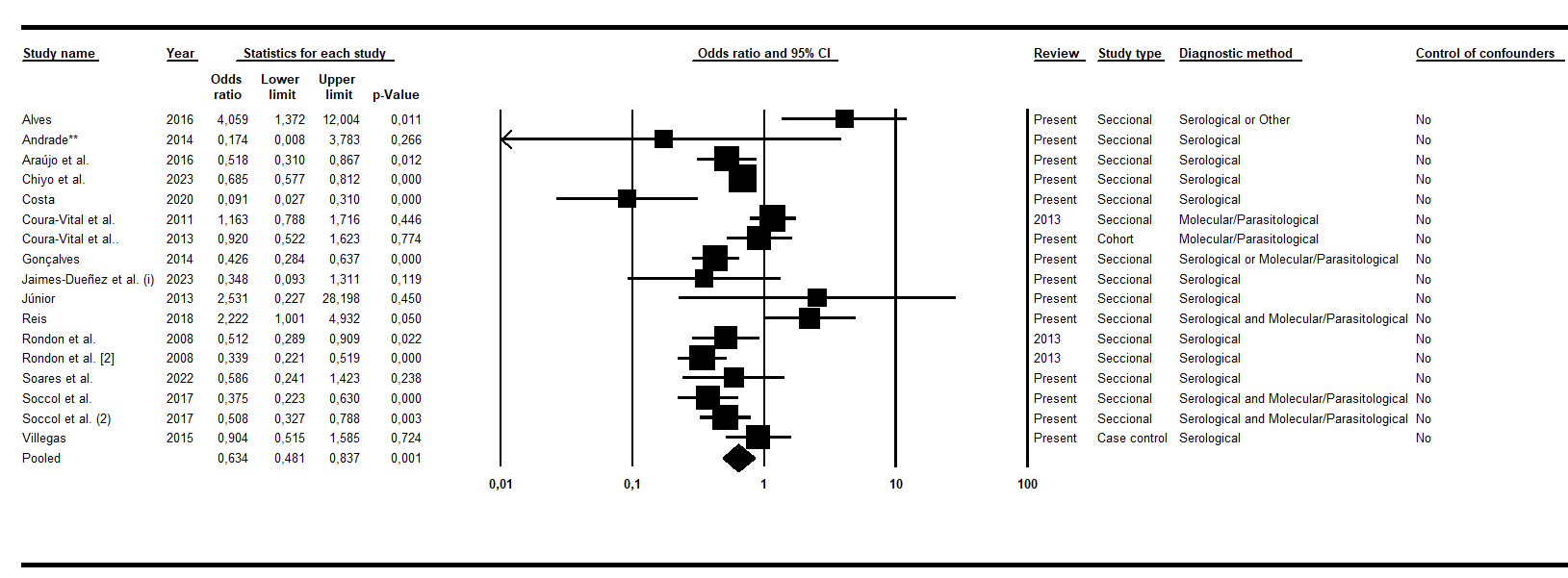
**

**Fig. S20.** Forest plot of the association between dog size and canine visceral leishmaniasis (small vs. large). Superscripts indicate: * result of a serological test in a study involving two diagnostic tests; ** result of a second serological test; *** result of a third serological test; (1) different studies by the same author in the same year; (2) second result reported in a single publication; (3) third result reported in a single publication; (i) second result from the same study; (ii) third result from the same study. Squares represent the weight of each study, whereas diamonds represent the pooled estimate for each subgroup. **Reference category:** large size (odds ratio = 1).


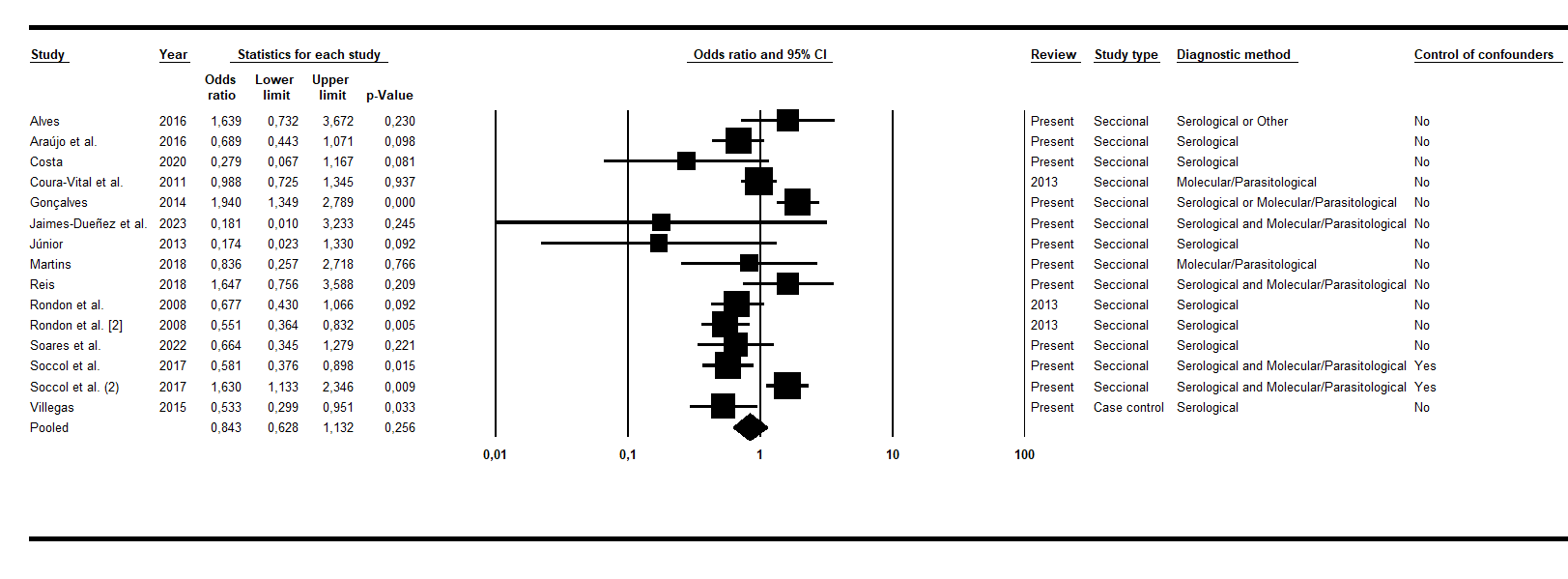


**Fig. S21.** Forest plot of the association between dog size and canine visceral leishmaniasis (small vs. medium). Superscripts indicate: * result of a serological test in a study involving two diagnostic tests; ** result of a second serological test; *** result of a third serological test; (1) different studies by the same author in the same year; (2) second result reported in a single publication; (3) third result reported in a single publication; (i) second result from the same study; (ii) third result from the same study. Squares represent the weight of each study, whereas diamonds represent the pooled estimate for each subgroup. **Reference category:** medium size (odds ratio = 1).


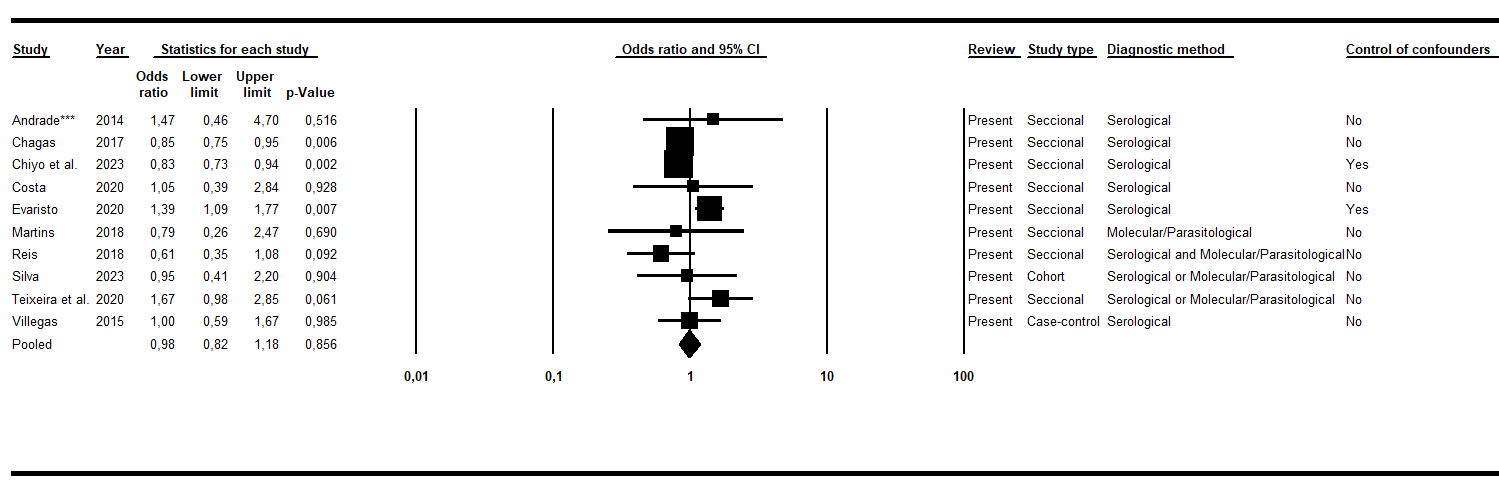


**Fig. S22.** Forest plot of the association between hair color and canine visceral leishmaniasis. Superscripts indicate: * result of a serological test in a study involving two diagnostic tests; ** result of a second serological test; *** result of a third serological test; (1) different studies by the same author in the same year; (2) second result reported in a single publication; (3) third result reported in a single publication; (i) second result from the same study; (ii) third result from the same study. Squares represent the weight of each study, whereas diamonds represent the pooled estimate for each subgroup. **Reference category**: dark hair (odds ratio = 1).
