# Supplementary material for: Risk and protective factors for canine visceral leishmaniasis in the Americas: a systematic review update with meta-analysis
Source: Parasit Vectors. 2026 Mar 18;19:185. doi: 10.1186/s13071-026-07325-0 (PMC13122873; doi:10.1186/s13071-026-07325-0)
Supplement: Supplementary file 6 — Additional file 6. Forest plots of variables stratified by subgroups (Figs. S1–S12). [file 13071_2026_7325_MOESM6_ESM.docx]

**Additional file 6: Forest plots of variables stratified by subgroups**

*Notes:*

*-Numerical values in the figures are presented with decimal commas due to software formatting and could not be modified*

*-The forest plots include studies from both the 2013 review (search completed up to September 2011) and the current (present) review (studies published from October 2011 up to June 2024).*


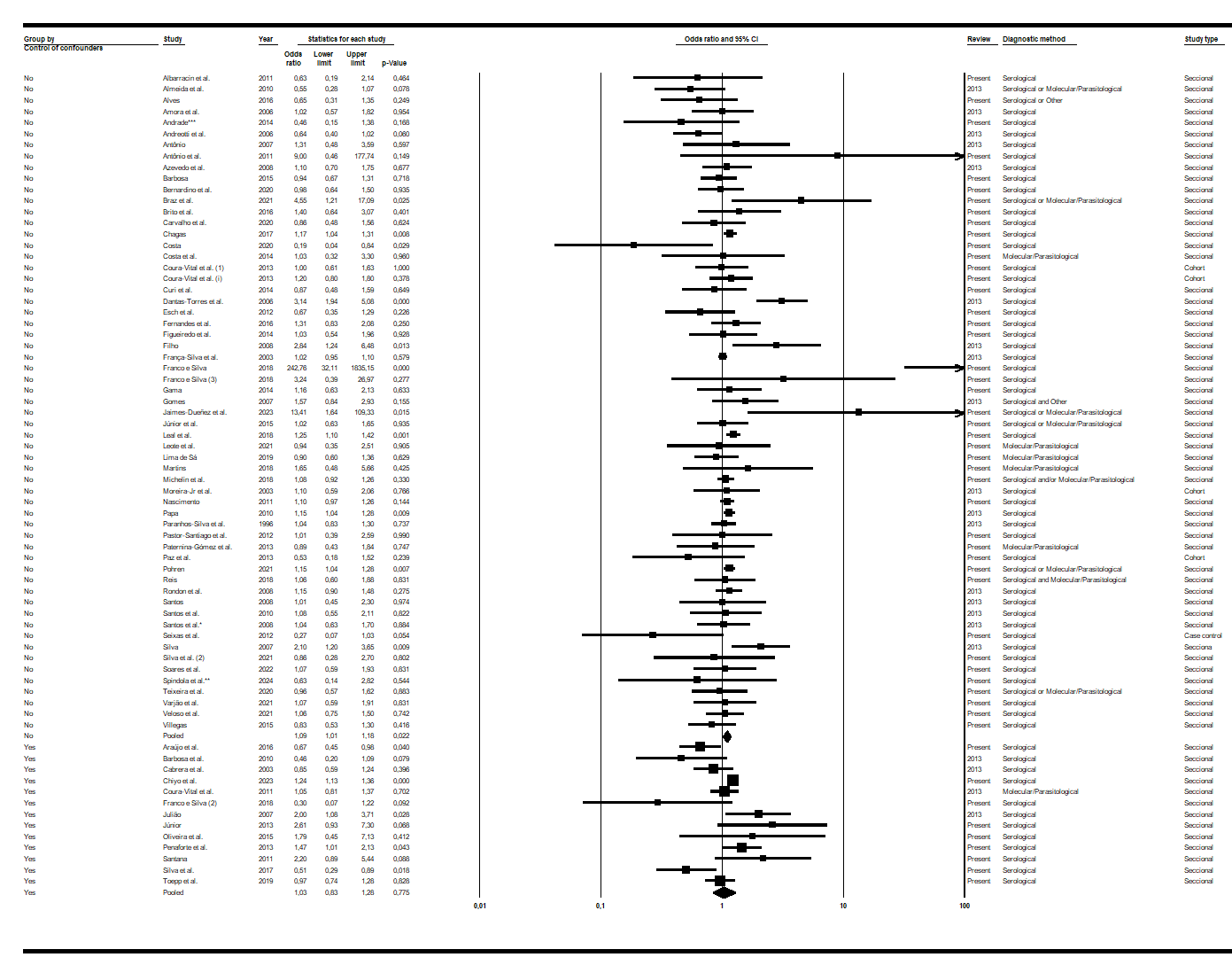


**Fig. S1.** Forest plot for the sex variable: studies divided into subgroups according to confounding control. Superscripts: * result of a serological test in a study involving two diagnostic tests; ** second different serological test result; *** third different serological test result; 1 different studies by the same author and year; 2 second result in a single publication; 3 third result in a single publication; i second result of the same study; ii third result of the same study. Squares represent the weight of each study, whereas diamonds represent the summary estimate of each subgroup. Reference: Female, odds ratio = 1.


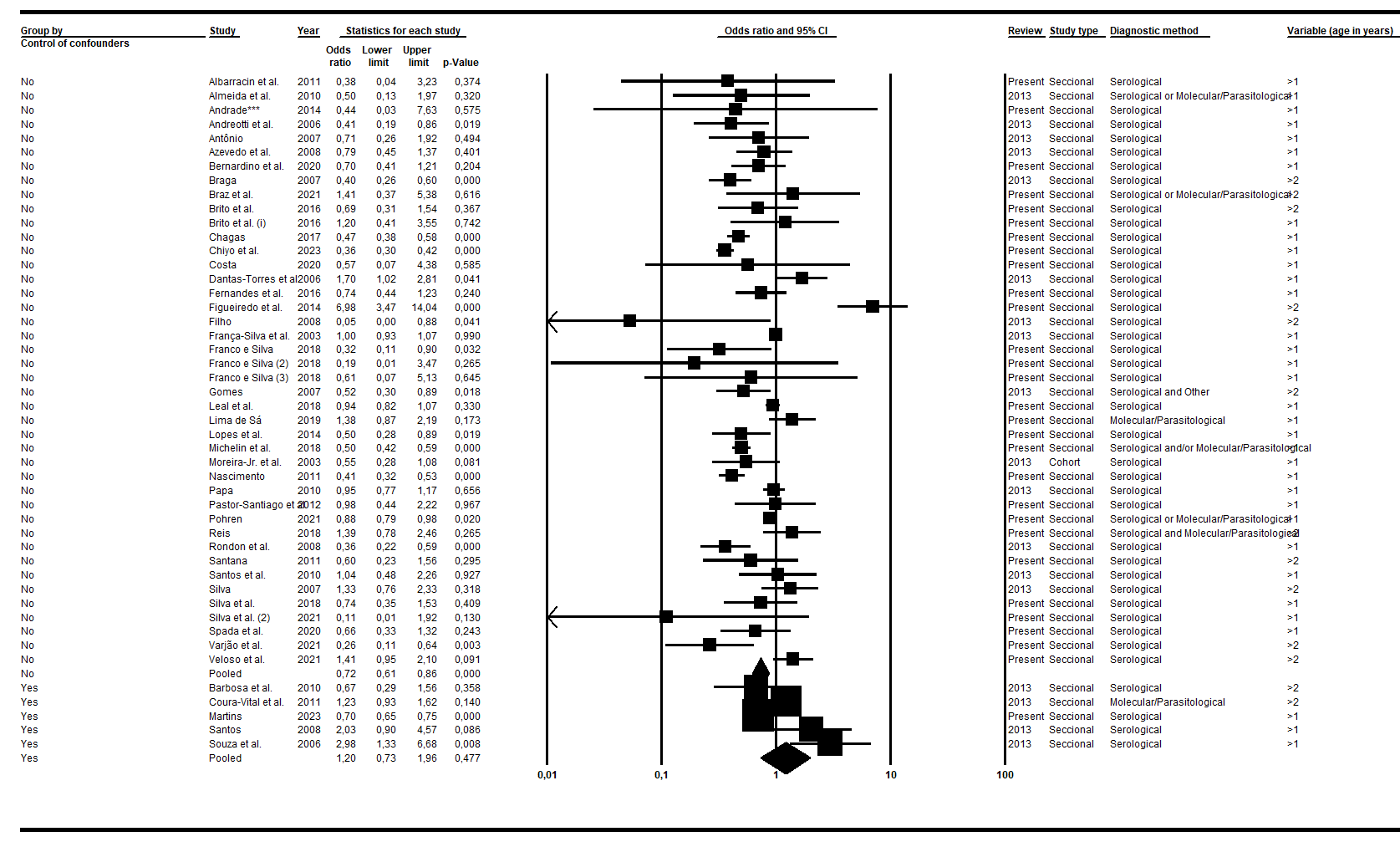


**Fig. S2.**  Forest plot for the age variable: studies divided into subgroups according to confounding control. Superscripts: * result of a serological test in a study involving two diagnostic tests; ** second different serological test result; *** third different serological test result; 1 different studies by the same author and year; 2 second result in a single publication; 3 third result in a single publication; i second result of the same study; ii third result of the same study. Squares represent the weight of each study, whereas diamonds represent the summary estimate of each subgroup. Reference: Older age, odds ratio = 1.

**
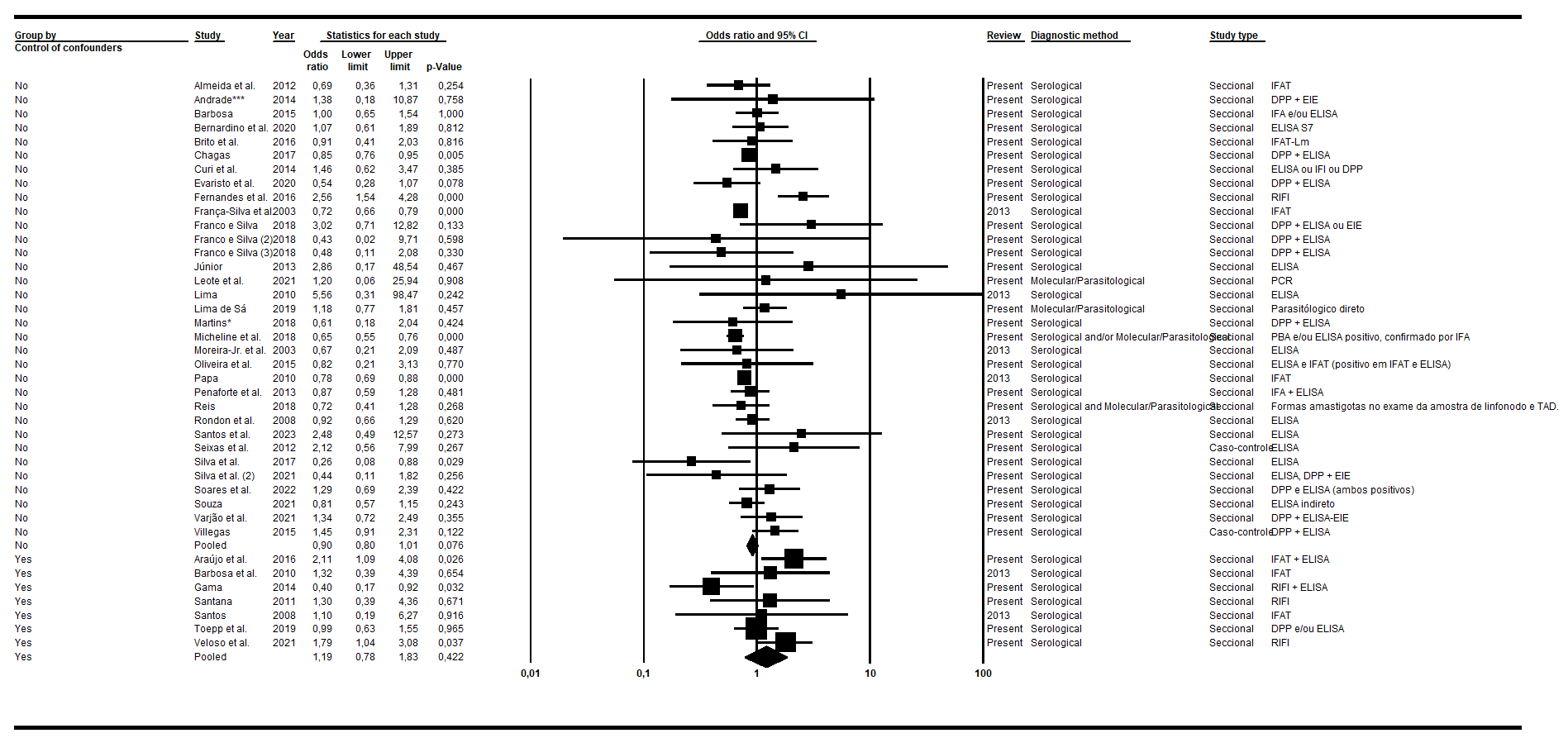
**

**Fig. S3.** Forest plot for the breed variable: studies divided into subgroups according to confounding control. Superscripts: * result of a serological test in a study involving two diagnostic tests; ** second different serological test result; *** third different serological test result; 1 different studies by the same author and year; 2 second result in a single publication; 3 third result in a single publication; i second result of the same study; ii third result of the same study. Squares represent the weight of each study, whereas diamonds represent the summary estimate of each subgroup. Reference: Defined breed, odds ratio = 1.= 1.


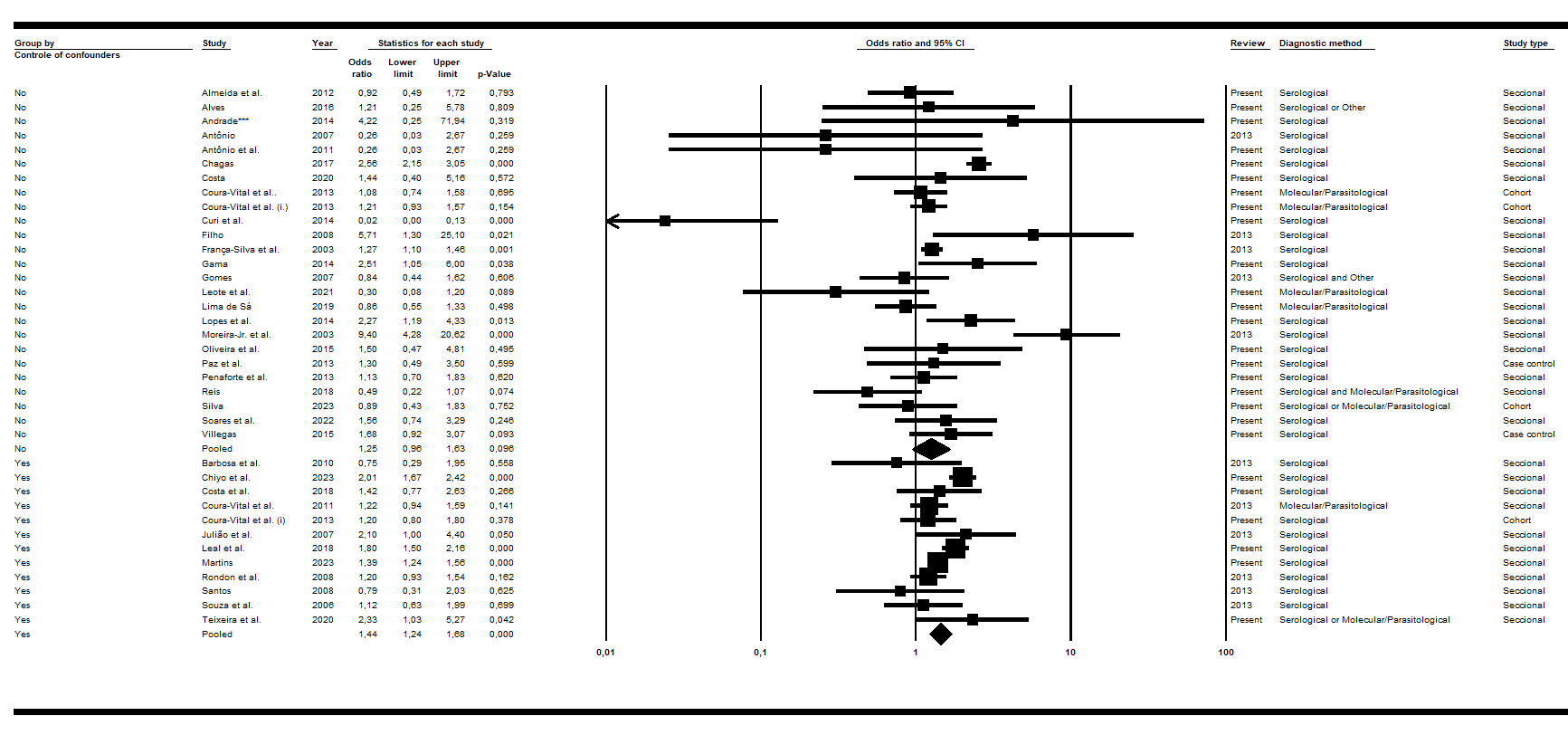


**Fig. S4.** Forest plot for the hair length variable: studies divided into subgroups according to confounding control. Superscripts: * result of a serological test in a study involving two diagnostic tests; ** second different serological test result; *** third different serological test result; 1 different studies by the same author and year; 2 second result in a single publication; 3 third result in a single publication; i second result of the same study; ii third result of the same study. Squares represent the weight of each study, whereas diamonds represent the summary estimate of each subgroup. Reference: Long hair, odds ratio = 1.


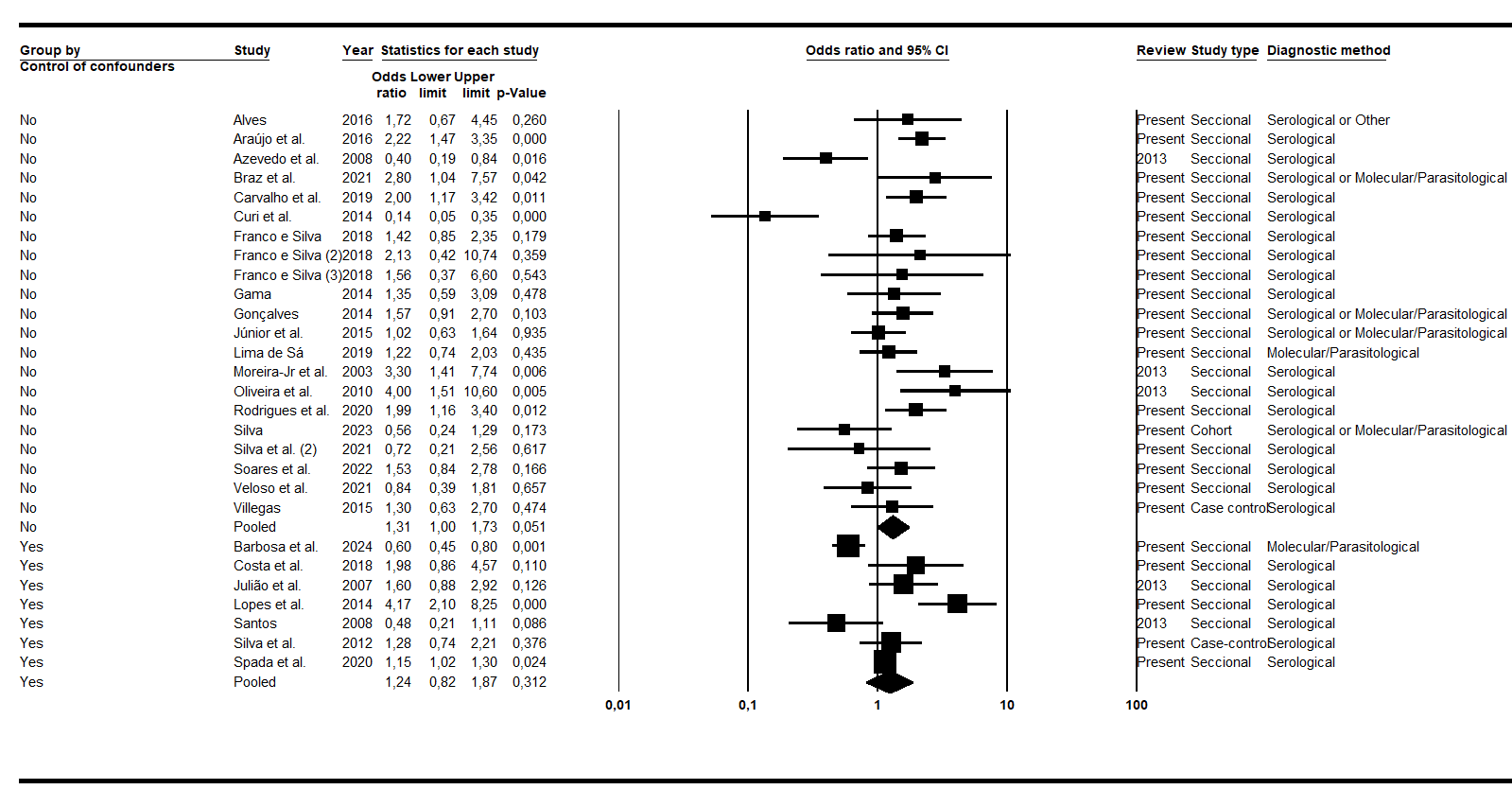


**Fig. S5.** Forest plot for the presence of chickens and/or chicken coops at the household variable: studies divided into subgroups according to confounding control. Superscripts: * result of a serological test in a study involving two diagnostic tests; ** second different serological test result; *** third different serological test result; 1 different studies by the same author and year; 2 second result in a single publication; 3 third result in a single publication; i second result of the same study; ii third result of the same study. Squares represent the weight of each study, whereas diamonds represent the summary estimate of each subgroup. Reference: No, odds ratio = 1.


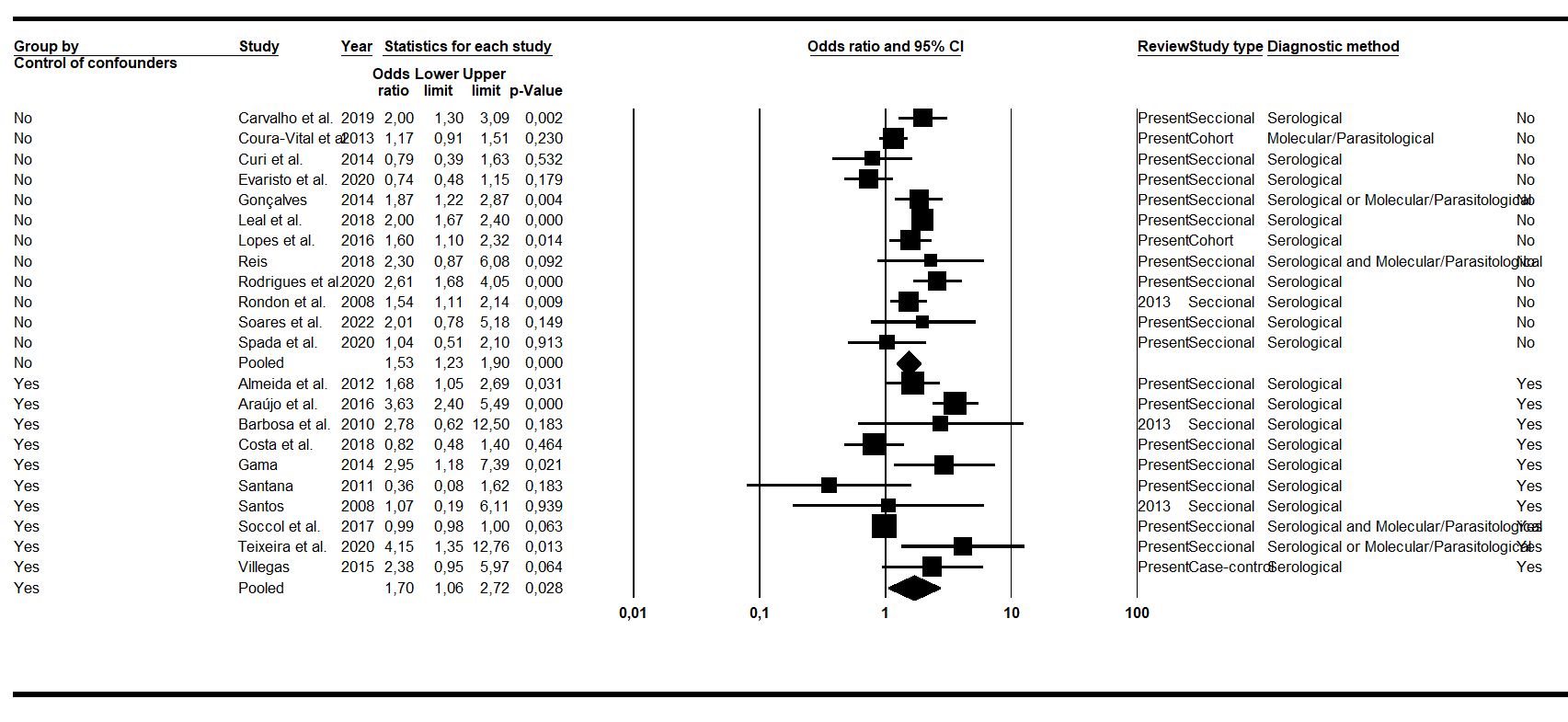


**Fig. S6.** Forest plot for the presence of vegetation variable: studies divided into subgroups according to confounding control. Superscripts: * result of a serological test in a study involving two diagnostic tests; ** second different serological test result; *** third different serological test result; 1 different studies by the same author and year; 2 second result in a single publication; 3 third result in a single publication; i second result of the same study; ii third result of the same study. Squares represent the weight of each study, whereas diamonds represent the summary estimate of each subgroup. Reference: No, odds ratio = 1.


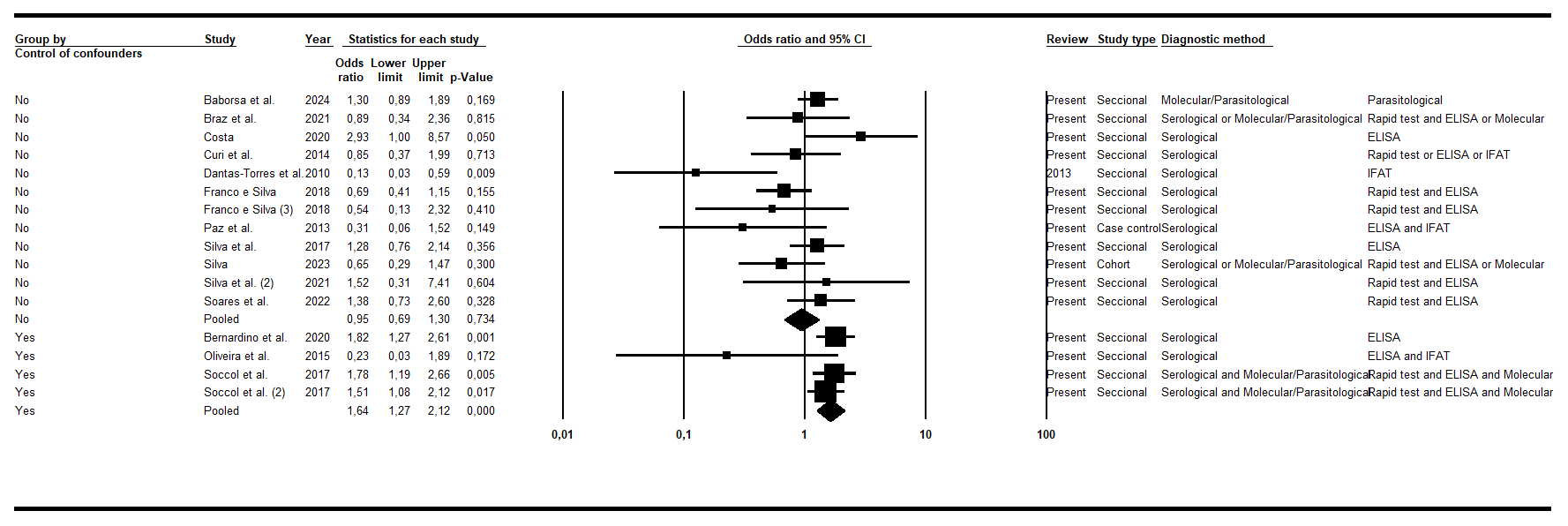


**Fig. S7.** Forest plot for the presence of ectoparasites variable: studies divided into subgroups according to confounding control. Superscripts: * result of a serological test in a study involving two diagnostic tests; ** second different serological test result; *** third different serological test result; 1 different studies by the same author and year; 2 second result in a single publication; 3 third result in a single publication; i second result of the same study; ii third result of the same study. Squares represent the weight of each study, whereas diamonds represent the summary estimate of each subgroup. Reference: No, odds ratio = 1.


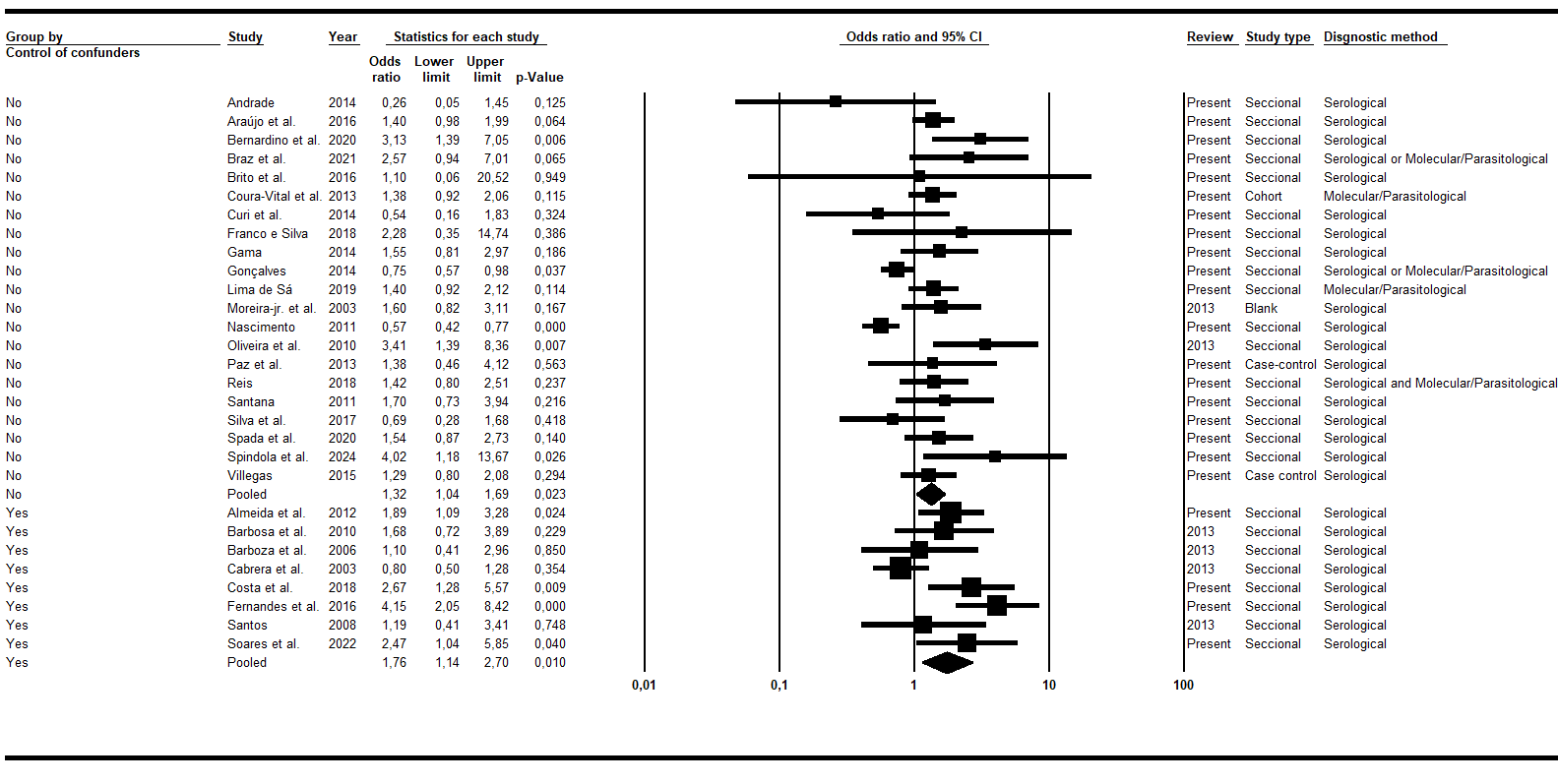


**Fig. S8.** Forest plot for the street access variable: studies divided into subgroups according to confounding control. Superscripts: * result of a serological test in a study involving two diagnostic tests; ** second different serological test result; *** third different serological test result; 1 different studies by the same author and year; 2 second result in a single publication; 3 third result in a single publication; i second result of the same study; ii third result of the same study. Squares represent the weight of each study, whereas diamonds represent the summary estimate of each subgroup. Reference: No, odds ratio = 1.


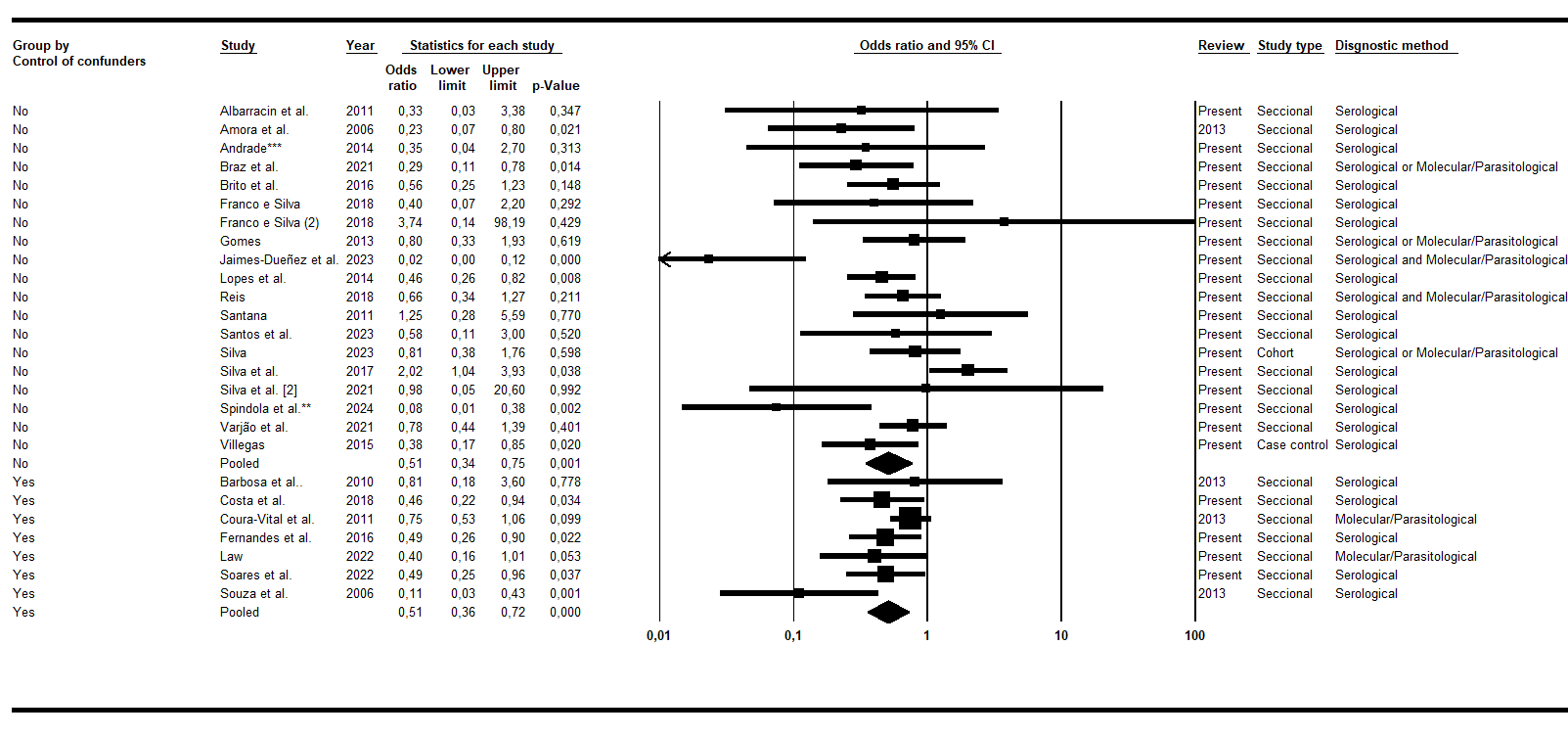


**Fig. S9** Forest plot for the dog’s dwelling area variable: studies divided into subgroups according to confounding control. Superscripts: * result of a serological test in a study involving two diagnostic tests; ** second different serological test result; *** third different serological test result; 1 different studies by the same author and year; 2 second result in a single publication; 3 third result in a single publication; i second result of the same study; ii third result of the same study. Squares represent the weight of each study, whereas diamonds represent the summary estimate of each subgroup. Reference: Peridomiciliary area, odds ratio = 1.


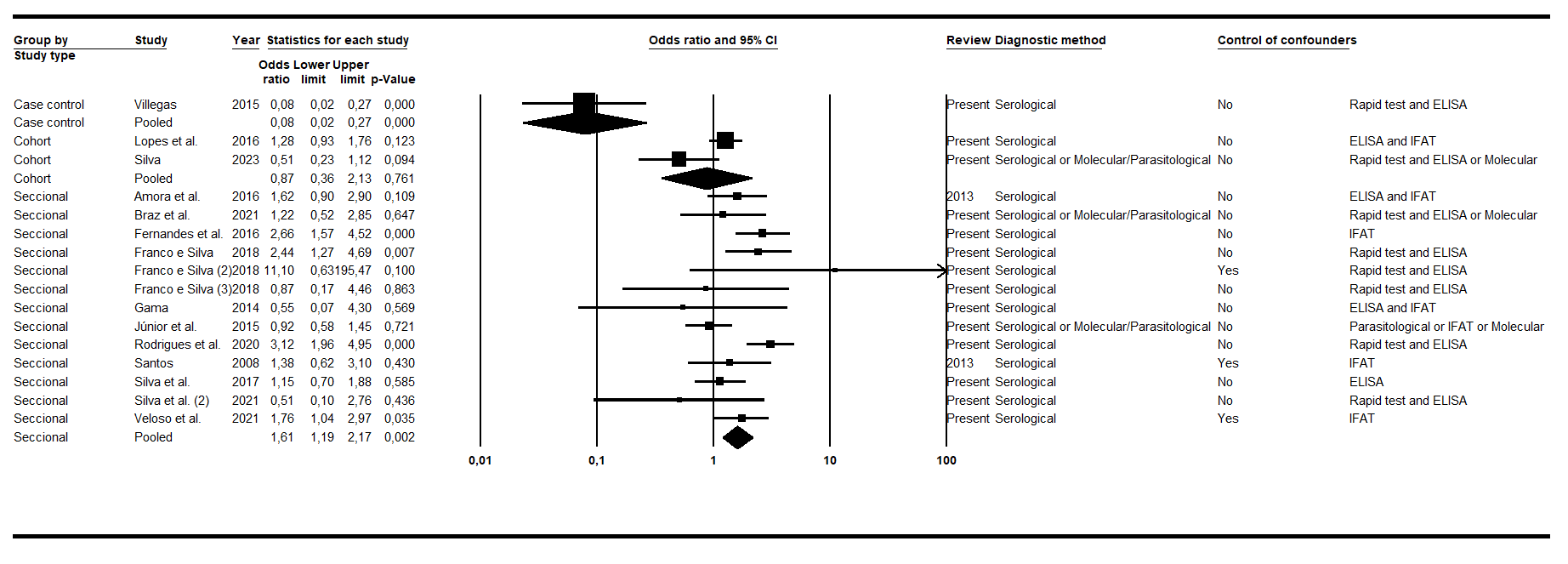


**Fig. S10.** Forest plot for the presence of other dogs in the household variable, with studies stratified by study design. Superscripts: * result of a serological test in a study involving two diagnostic tests; ** second different serological test result; *** third different serological test result; 1 different studies by the same author and year; 2 second result in a single publication; 3 third result in a single publication; i second result of the same study; ii third result of the same study. Squares represent the weight of each study, whereas diamonds represent the summary estimate of each subgroup. Reference: No, odds ratio = 1.


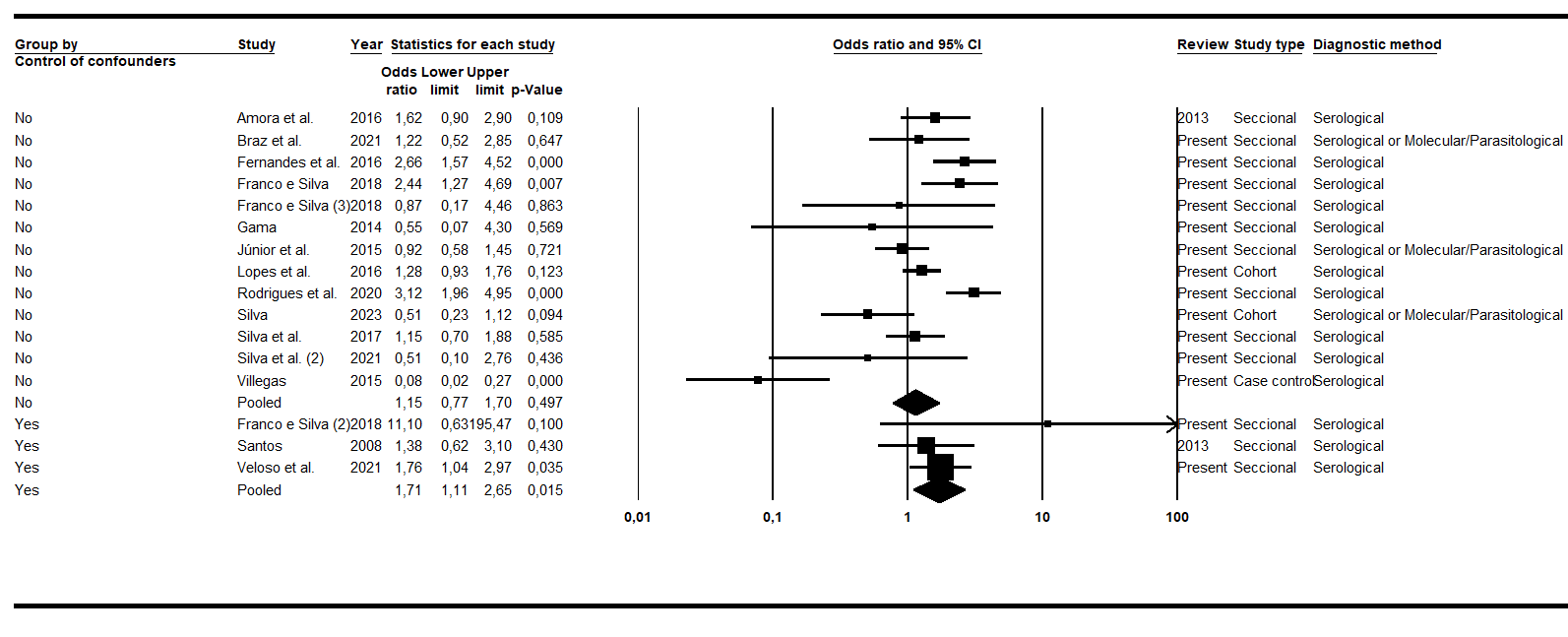


**Fig. S11.** Forest plot for the presence of other dogs in the household variable: studies divided into subgroups according to confounding control. Superscripts: * result of a serological test in a study involving two diagnostic tests; ** second different serological test result; *** third different serological test result; 1 different studies by the same author and year; 2 second result in a single publication; 3 third result in a single publication; i second result of the same study; ii third result of the same study. Squares represent the weight of each study, whereas diamonds represent the summary estimate of each subgroup. Reference: No, odds ratio = 1.


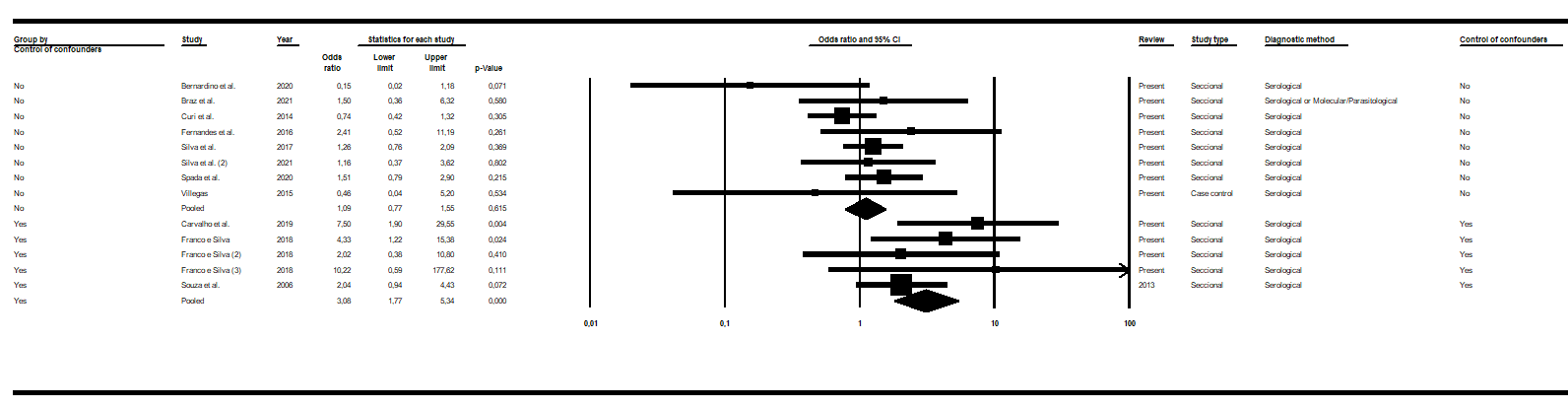


**Fig. S12.** Forest plot for the presence of horses variable: studies divided into subgroups according to confounding control. Superscripts: * result of a serological test in a study involving two diagnostic tests; ** second different serological test result; *** third different serological test result; 1 different studies by the same author and year; 2 second result in a single publication; 3 third result in a single publication; i second result of the same study; ii third result of the same study. Squares represent the weight of each study, whereas diamonds represent the summary estimate of each subgroup. Reference: No, odds ratio = 1.
