# Supplementary material for: Risk and protective factors for canine visceral leishmaniasis in the Americas: a systematic review update with meta-analysis
Source: Parasit Vectors. 2026 Mar 18;19:185. doi: 10.1186/s13071-026-07325-0 (PMC13122873; doi:10.1186/s13071-026-07325-0)
Supplement: Supplementary file 7 — Additional file 7. Funnel plots for assessment of publication bias (Figs. S1–S9). [file 13071_2026_7325_MOESM7_ESM.docx]

**Additional file 7: Funnel plots for assessment of publication bias**


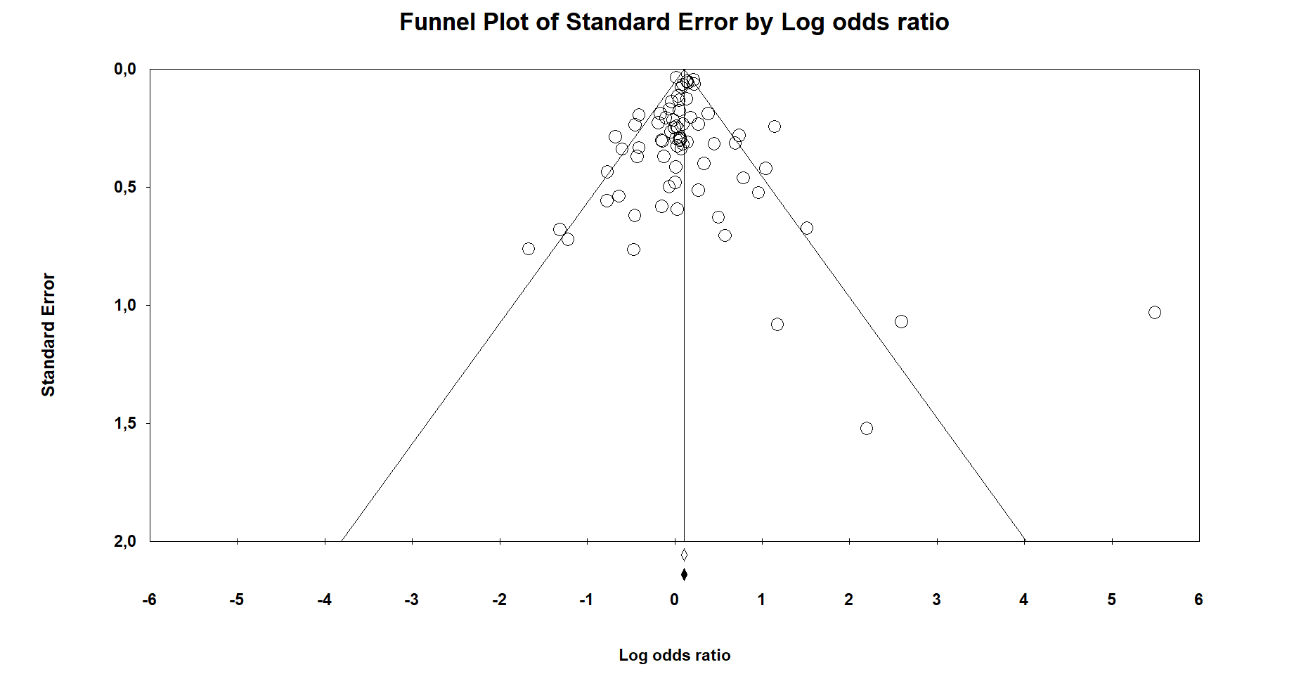


**Fig. S1.** Funnel plot for the sex variable. Closed dots correspond to imputed studies using the “trim and fill” method of Duval and Tweedie.


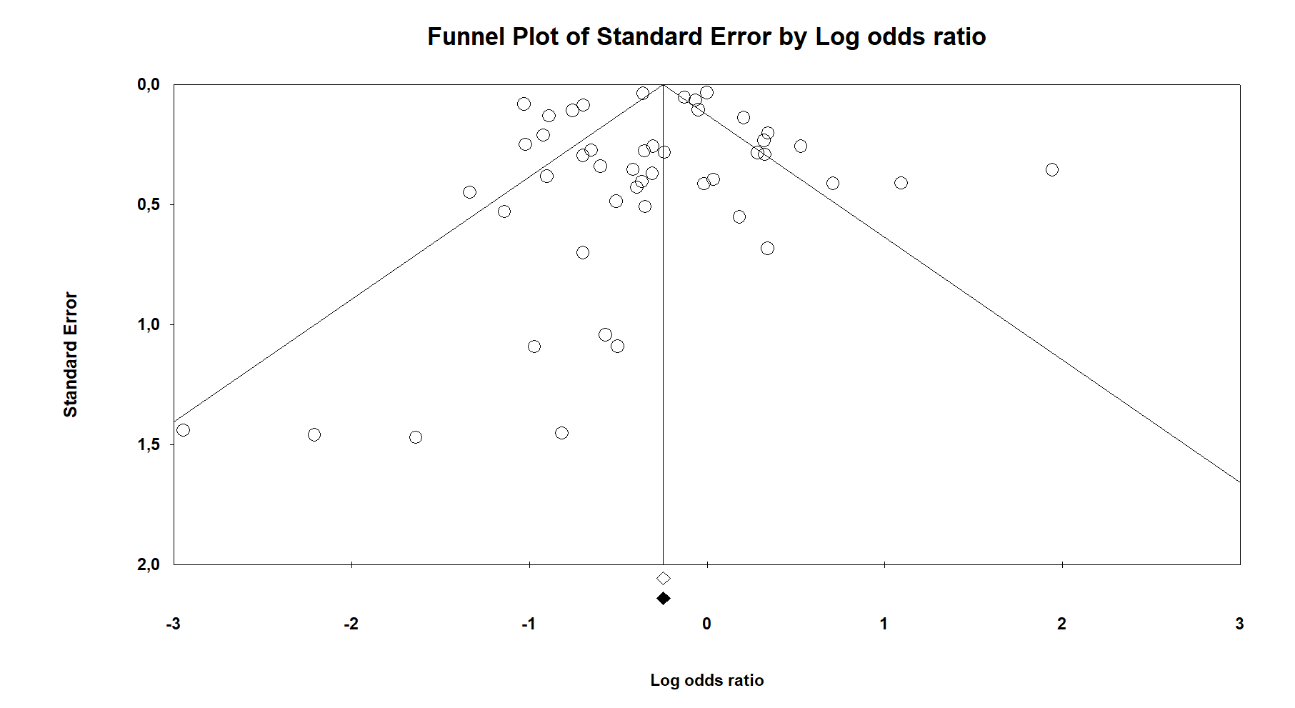


**Fig. S2.** Funnel plot for the age variable. Closed dots correspond to imputed studies using the “trim and fill” method of Duval and Tweedie.


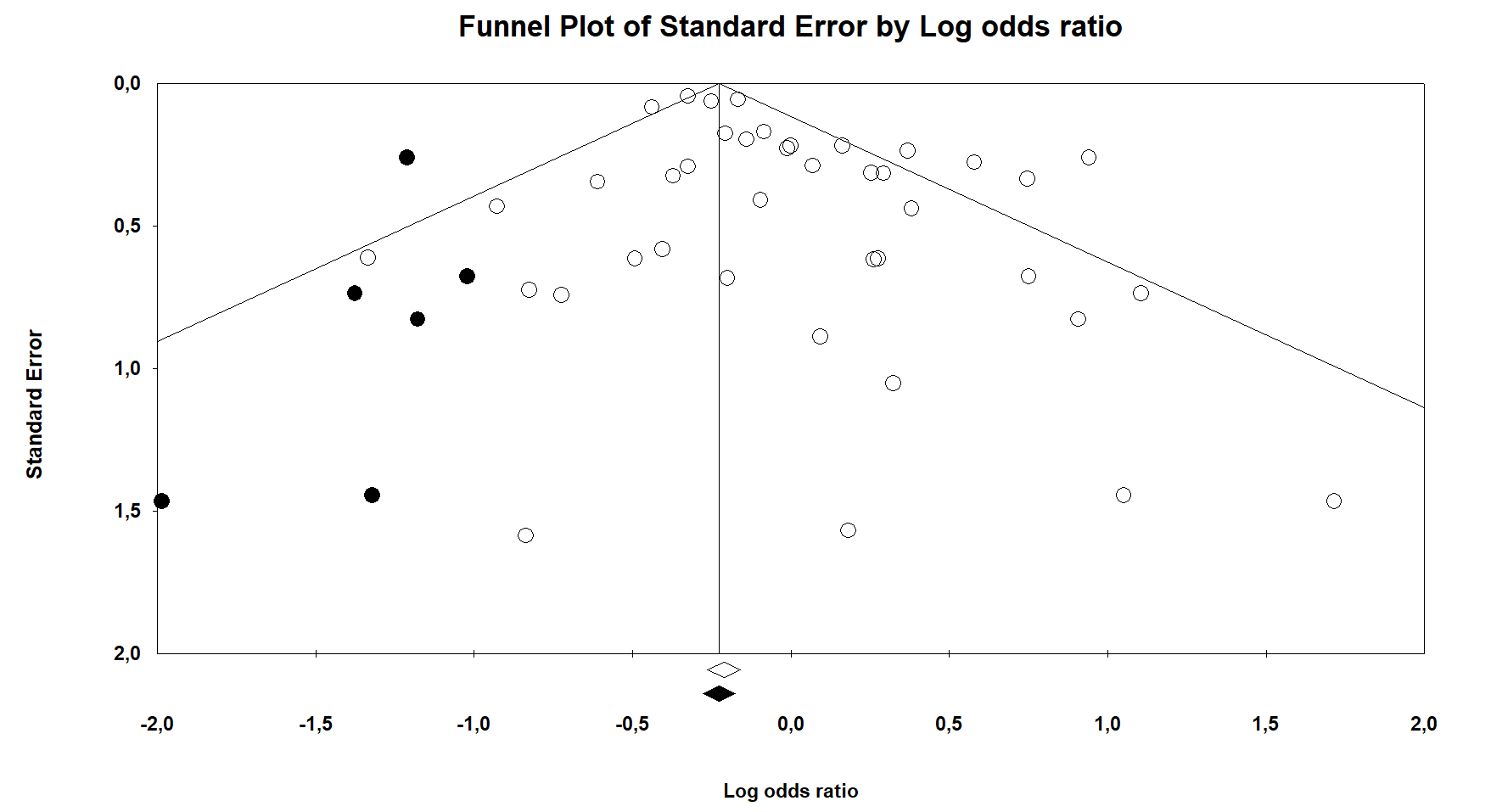


**Fig. S3.** Funnel plot for the breed variable. Closed dots correspond to imputed studies using the “trim and fill” method of Duval and Tweedie.


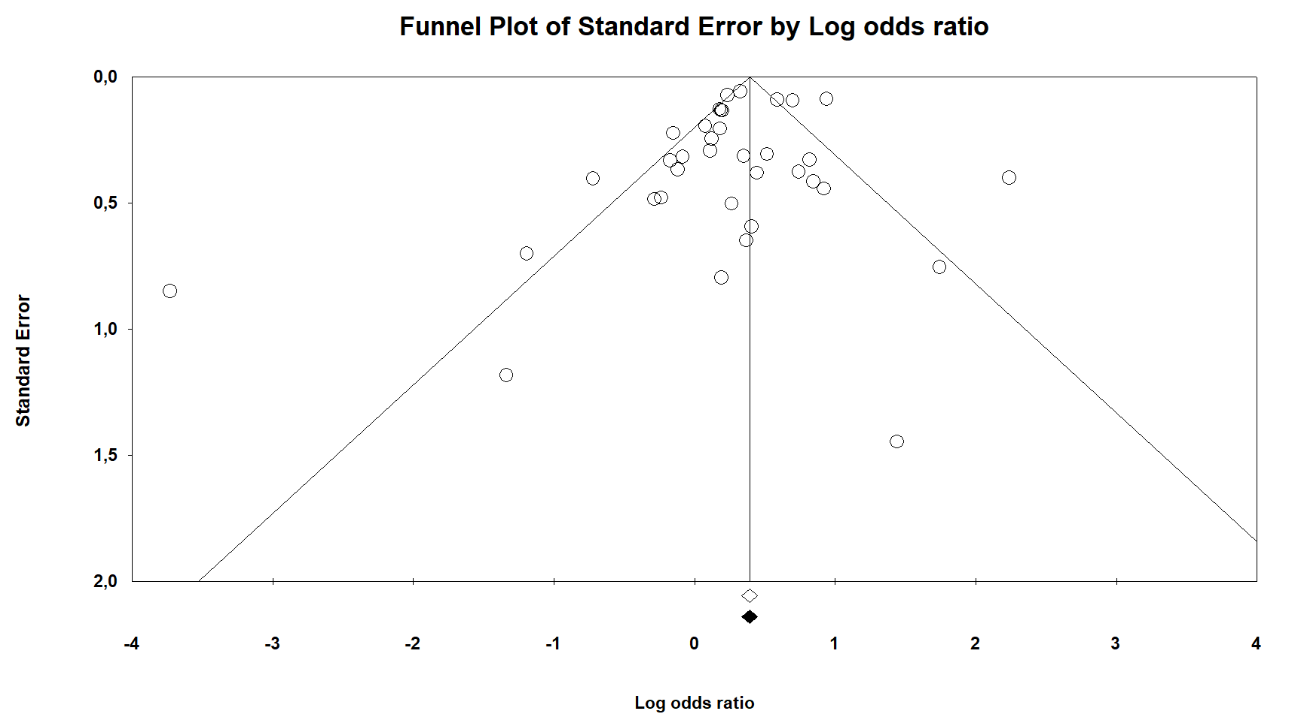


**Fig. S4.** Funnel plot for the hair length variable. Closed dots correspond to imputed studies using the “trim and fill” method of Duval and Tweedie.


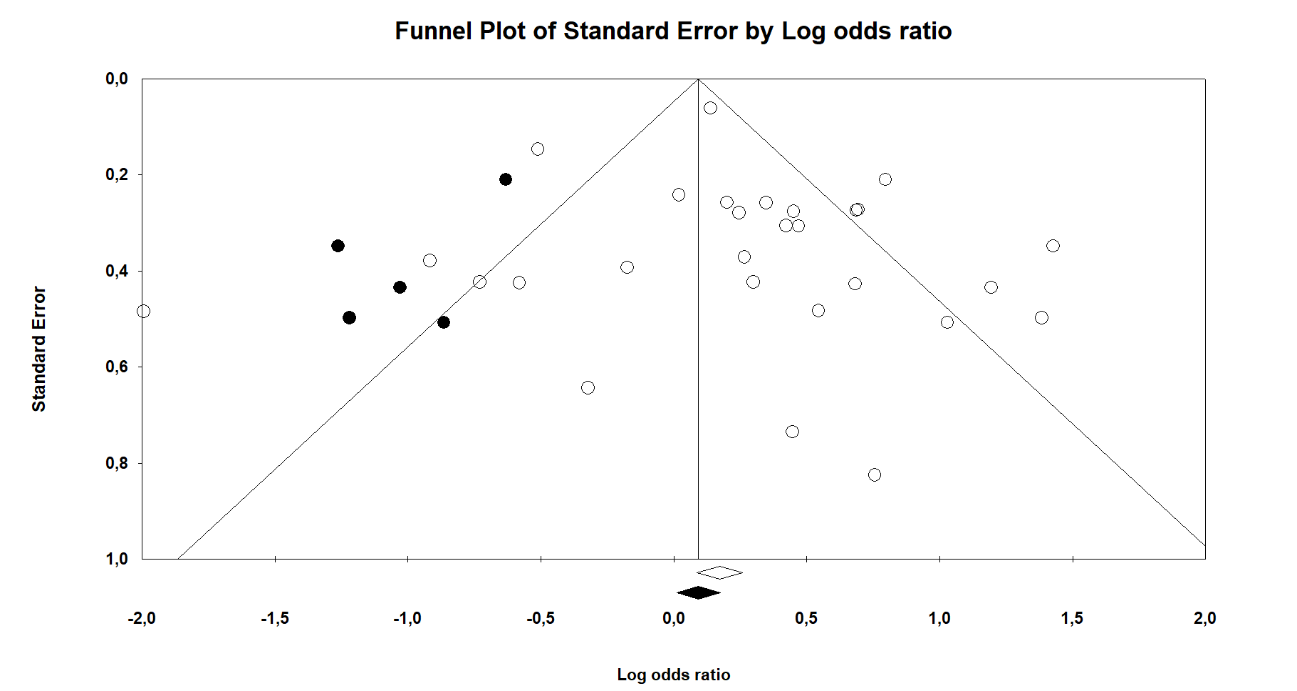


**Fig. S5.** Funnel plot for the presence of chickens and/or chicken coop in the home variable. Closed dots correspond to imputed studies using the “trim and fill” method of Duval and Tweedie.


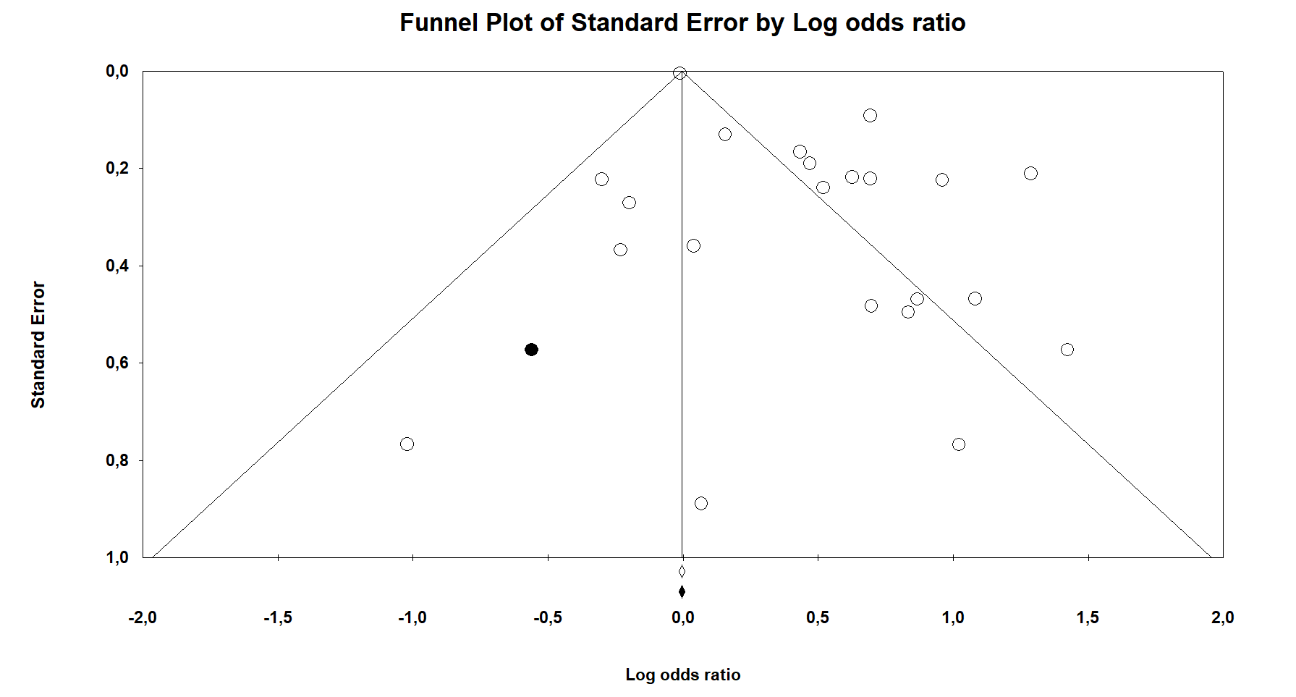


**Fig. S6.** Funnel plot for the presence of vegetation variable. Closed dots correspond to imputed studies using the “trim and fill” method of Duval and Tweedie.


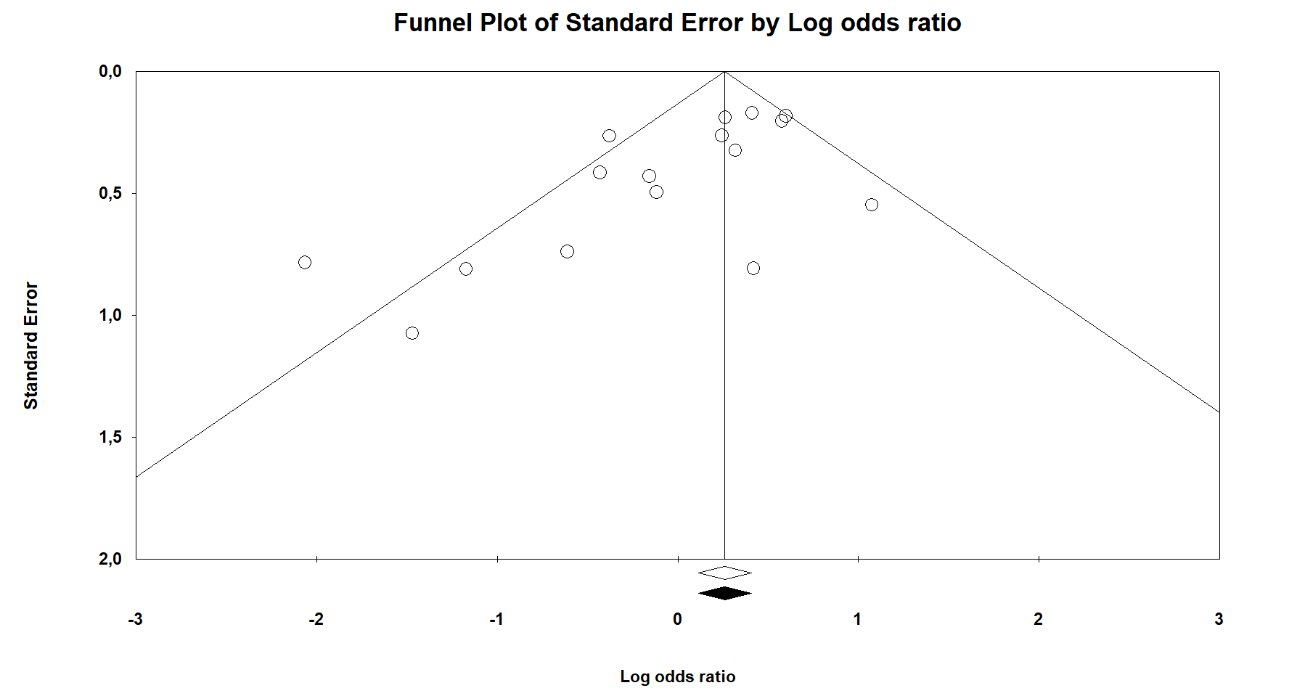


**Fig. S7.** Funnel plot for the presence of ectoparasites variable. Closed dots correspond to imputed studies using the “trim and fill” method of Duval and Tweedie.


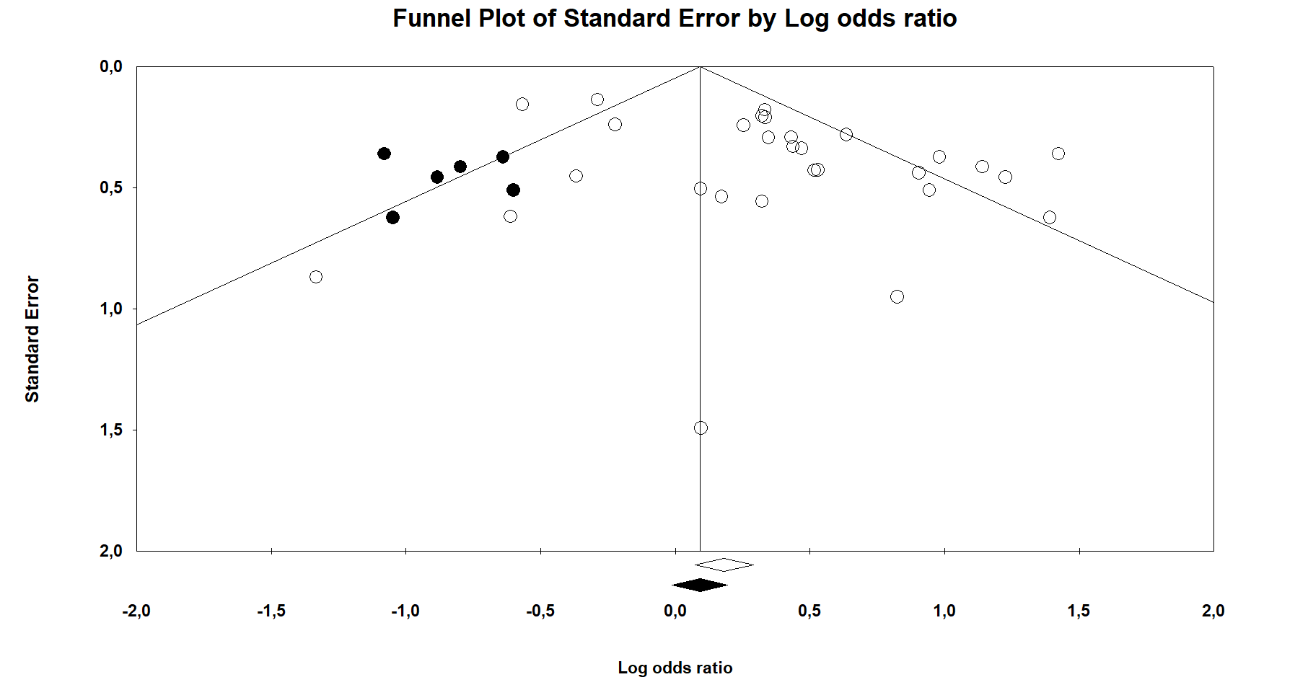


**Fig. S8.** Funnel plot for the street access variable. Closed dots correspond to imputed studies using the “trim and fill” method of Duval and Tweedie.


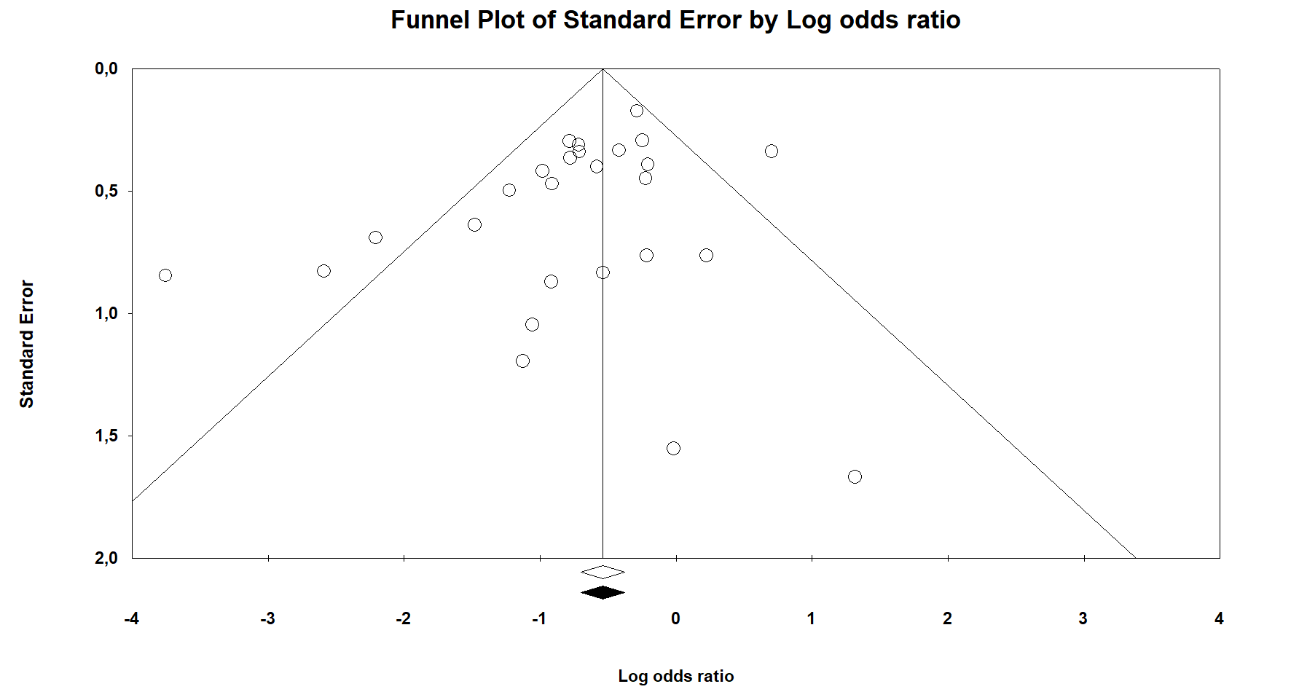


**Fig. S9.** Funnel plot for the dog’s dwelling area variable. Closed dots correspond to imputed studies using the “trim and fill” method of Duval and Tweedie.
