# Supplementary material for: Risk and protective factors for canine visceral leishmaniasis in the Americas: a systematic review update with meta-analysis
Source: Parasit Vectors. 2026 Mar 18;19:185. doi: 10.1186/s13071-026-07325-0 (PMC13122873; doi:10.1186/s13071-026-07325-0)
Supplement: Supplementary file 8 — Additional file 8. Forest plots of socioeconomic variables (Figs. S1–S5). [file 13071_2026_7325_MOESM8_ESM.docx]

**Additional file 8: Forest plots of socioeconomic variables**

*Notes:*

*-Numerical values in the figures are presented with decimal commas due to software formatting and could not be modified*

*-The forest plots include studies from both the 2013 review (search completed up to September 2011) and the current (present) review (studies published from October 2011 up to June 2024).*


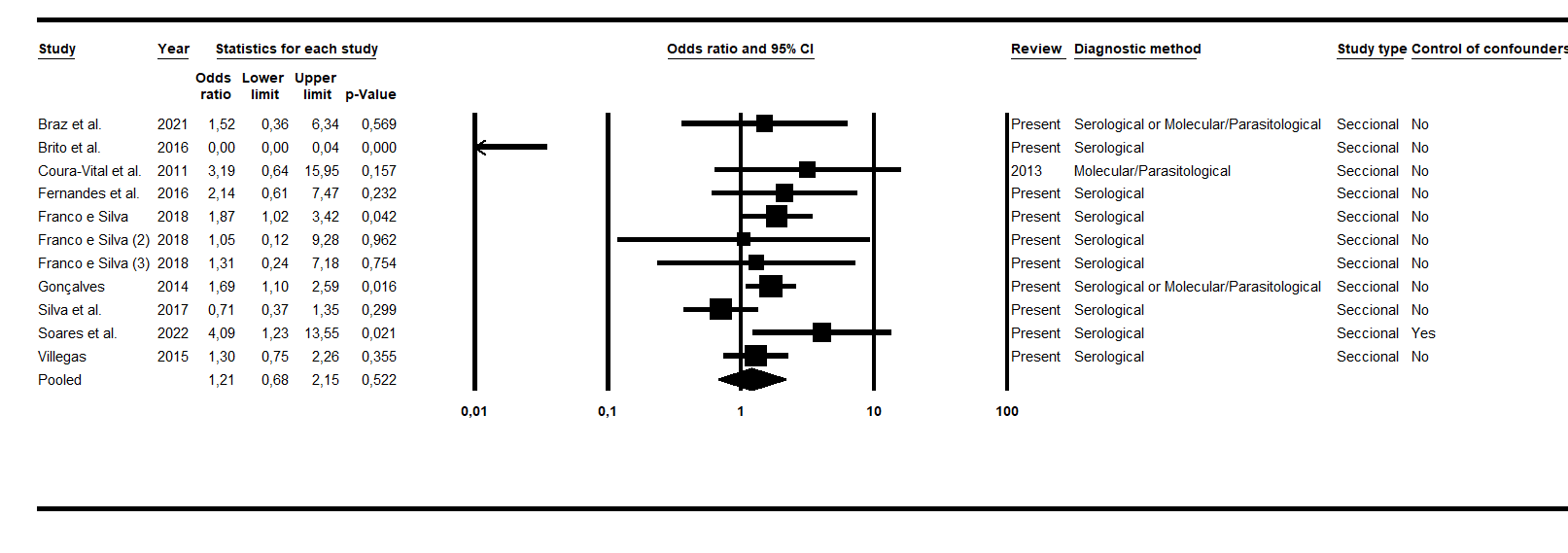


**Fig. S1.** Forest plot for the level of education variable. Superscripts: * result of a serological test in a study involving two diagnostic tests; ** second different serological test result; *** third different serological test result; 1 different studies by the same author and year; 2 second result in a single publication; 3 third result in a single publication; i second result of the same study; ii third result of the same study. Squares represent the weight of each study, whereas diamonds represent the summary estimate of each subgroup. Reference: Some schooling, odds ratio = 1.


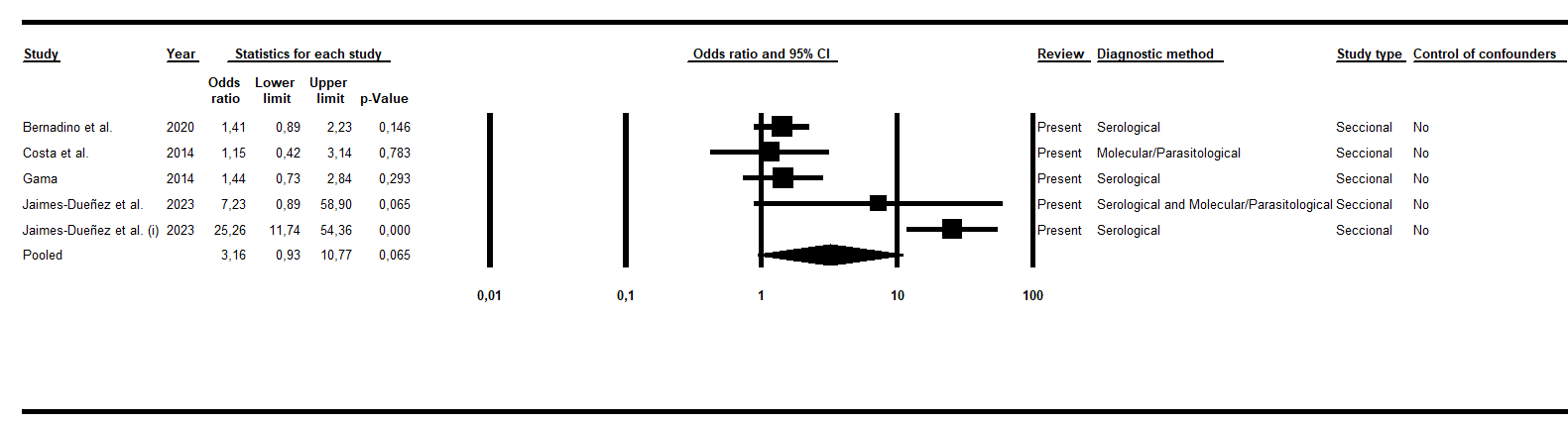


**Fig. S2.** Forest plot for the income variable. Superscripts: * result of a serological test in a study involving two diagnostic tests; ** second different serological test result; *** third different serological test result; 1 different studies by the same author and year; 2 second result in a single publication; 3 third result in a single publication; i second result of the same study; ii third result of the same study. Squares represent the weight of each study, whereas diamonds represent the summary estimate of each subgroup. Reference: >1 minimum wage, odds ratio = 1.


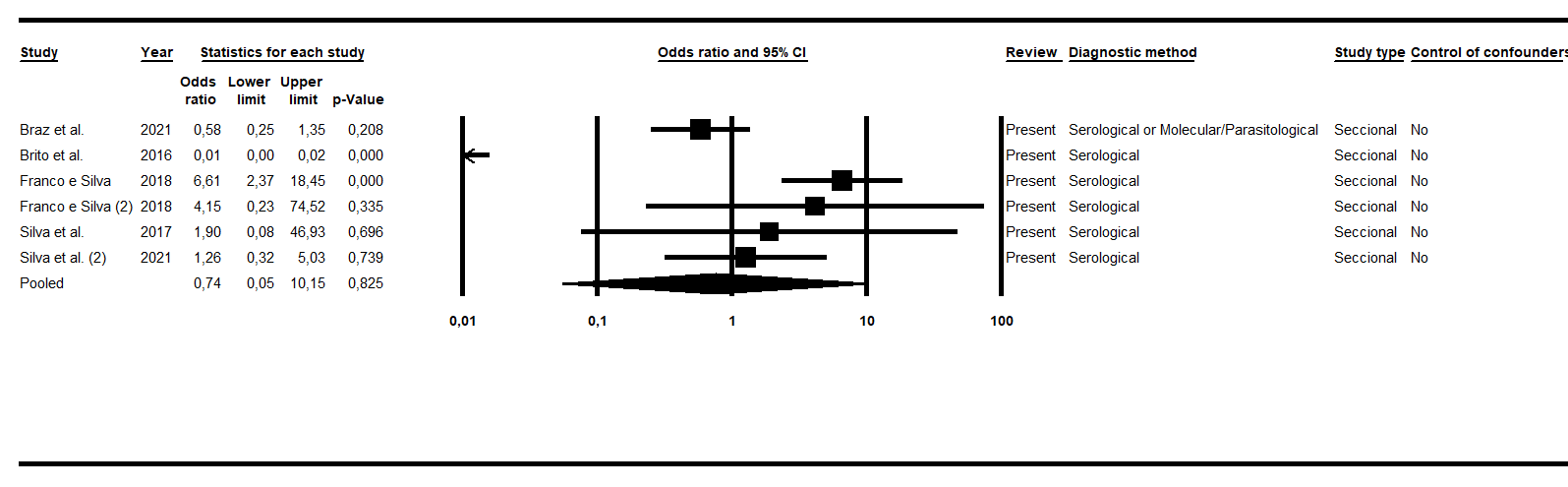


**Fig. S3.** Forest plot for the income variable. Superscripts: * result of a serological test in a study involving two diagnostic tests; ** second different serological test result; *** third different serological test result; 1 different studies by the same author and year; 2 second result in a single publication; 3 third result in a single publication; i second result of the same study; ii third result of the same study. Squares represent the weight of each study, whereas diamonds represent the summary estimate of each subgroup. Reference: ≥2 minimum wages, odds ratio = 1.


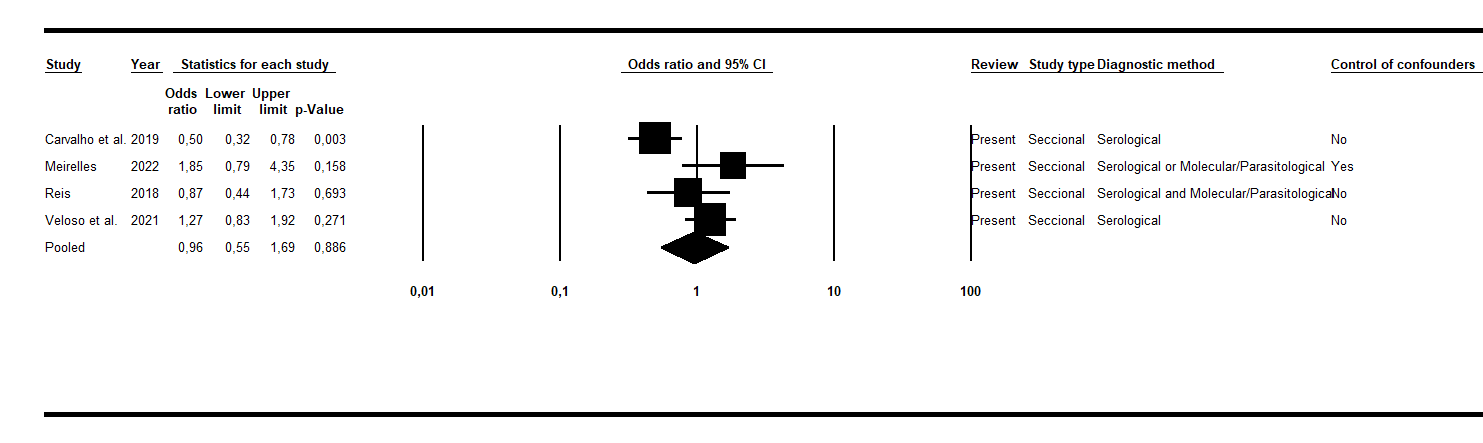


**Fig. S4.** Forest plot for the sewage collection variable. Superscripts: * result of a serological test in a study involving two diagnostic tests; ** second different serological test result; *** third different serological test result; 1 different studies by the same author and year; 2 second result in a single publication; 3 third result in a single publication; i second result of the same study; ii third result of the same study. Squares represent the weight of each study, whereas diamonds represent the summary estimate of each subgroup. Reference: Inadequate, odds ratio = 1.


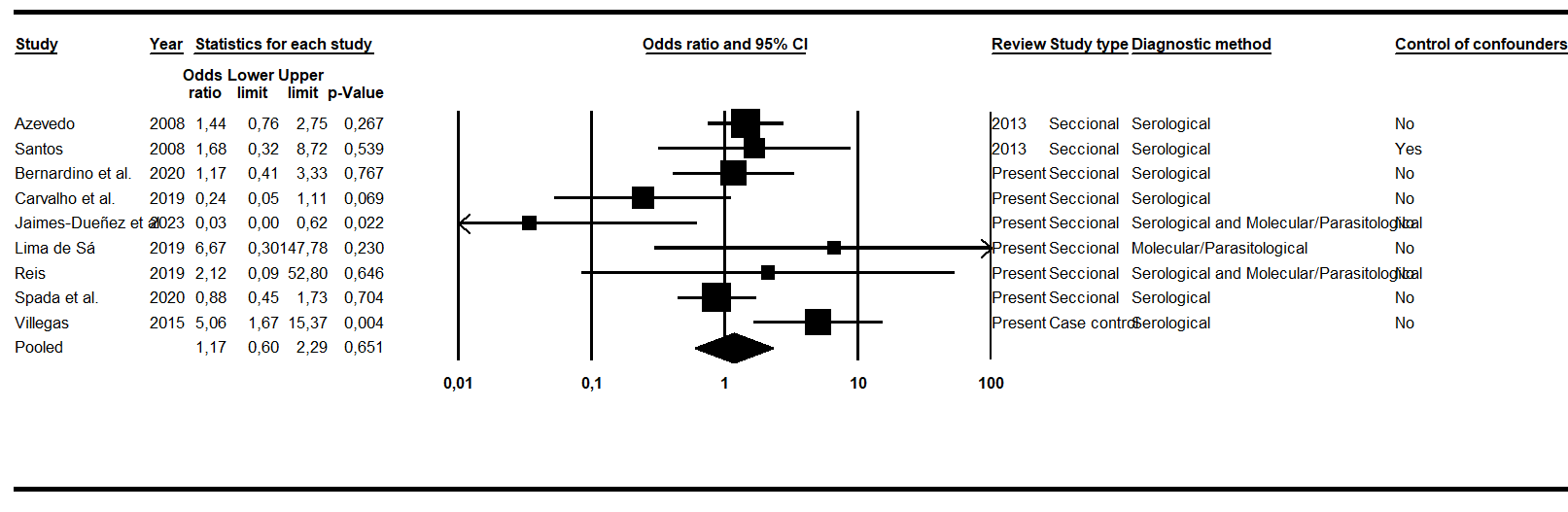


**Fig. S5.** Forest plot for the garbage collection variable. Superscripts: * result of a serological test in a study involving two diagnostic tests; ** second different serological test result; *** third different serological test result; 1 different studies by the same author and year; 2 second result in a single publication; 3 third result in a single publication; i second result of the same study; ii third result of the same study. Squares represent the weight of each study, whereas diamonds represent the summary estimate of each subgroup. Reference: No, odds ratio = 1.
