# Supplementary material for: Risk and protective factors for canine visceral leishmaniasis in the Americas: a systematic review update with meta-analysis
Source: Parasit Vectors. 2026 Mar 18;19:185. doi: 10.1186/s13071-026-07325-0 (PMC13122873; doi:10.1186/s13071-026-07325-0)
Supplement: Supplementary file 9 — Additional file 9. Forest plots of other variables (Figs. S1–S15). [file 13071_2026_7325_MOESM9_ESM.docx]

**Additional file 9: Forest plots of other variables**

*Notes:*

*-Numerical values in the figures are presented with decimal commas due to software formatting and could not be modified*

*-The forest plots include studies from both the 2013 review (search completed up to September 2011) and the current (present) review (studies published from October 2011 up to June 2024).*

**
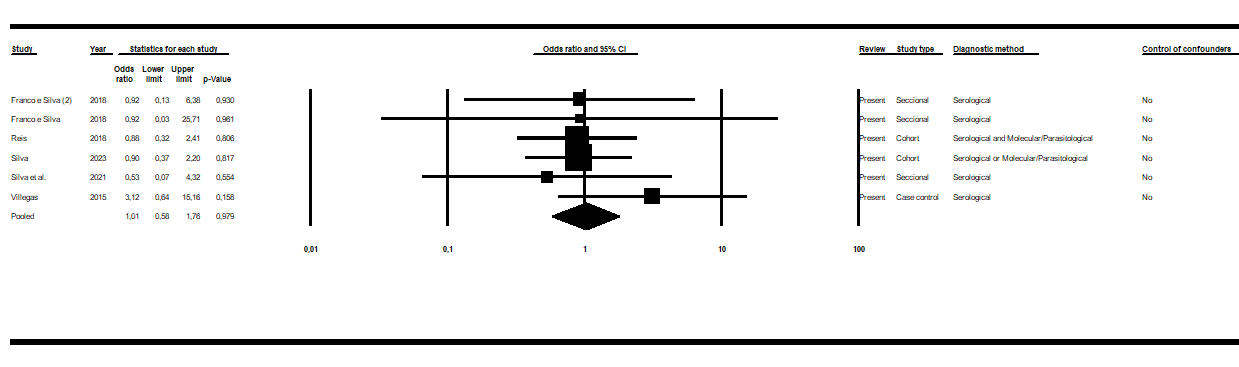
**

**Fig. S1.** Forest plot for the sleeping place (street vs indoors) variable. Superscripts: * result of a serological test in a study involving two diagnostic tests; ** second different serological test result; *** third different serological test result; 1 different studies by the same author and year; 2 second result in a single publication; 3 third result in a single publication; i second result of the same study; ii third result of the same study. Squares represent the weight of each study, whereas diamonds represent the summary estimate of each subgroup. Reference: Indoors, odds ratio = 1.


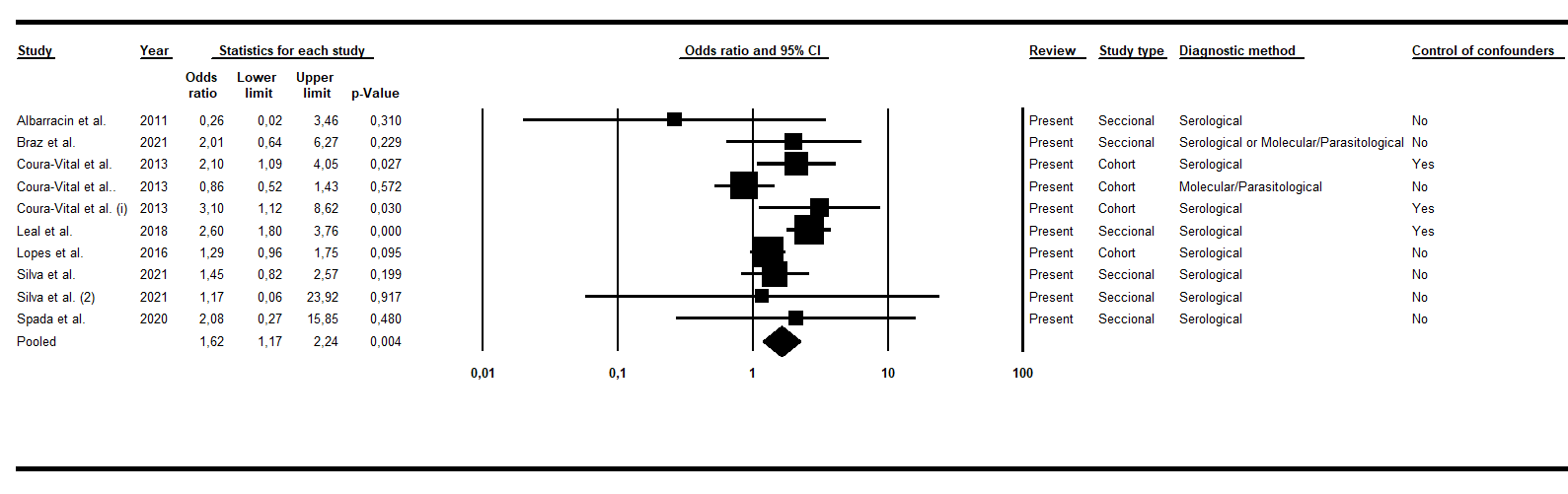


**Fig. S2.** Forest plot for the sleeping place (peridomicile vs inside the house) variable. Superscripts: * result of a serological test in a study involving two diagnostic tests; ** second different serological test result; *** third different serological test result; 1 different studies by the same author and year; 2 second result in a single publication; 3 third result in a single publication; i second result of the same study; ii third result of the same study. Squares represent the weight of each study, whereas diamonds represent the summary estimate of each subgroup. Reference: Inside the house, odds ratio = 1.

**
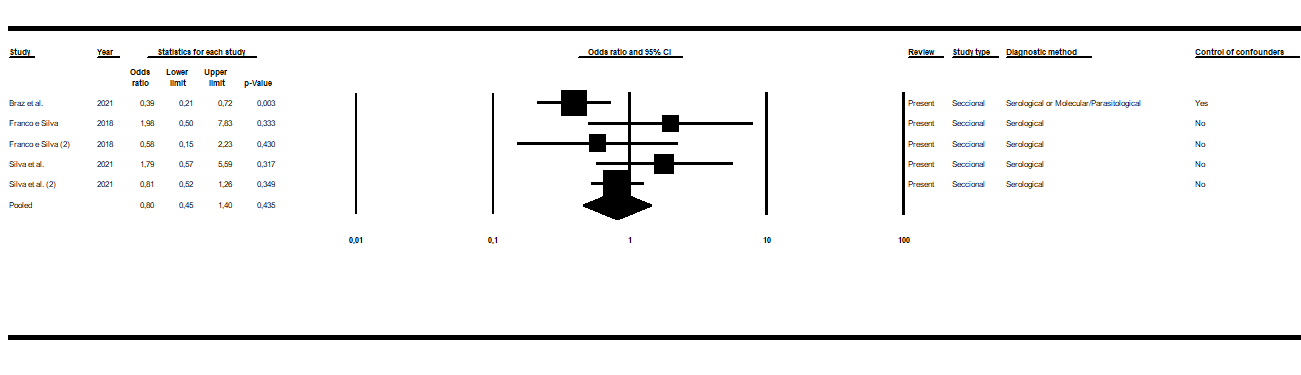
**

**Fig. S3.** Forest plot for the dog’s nighttime restraint variable. Superscripts: * result of a serological test in a study involving two diagnostic tests; ** second different serological test result; *** third different serological test result; 1 different studies by the same author and year; 2 second result in a single publication; 3 third result in a single publication; i second result of the same study; ii third result of the same study. Squares represent the weight of each study, whereas diamonds represent the summary estimate of each subgroup. Reference: Tied, odds ratio = 1..


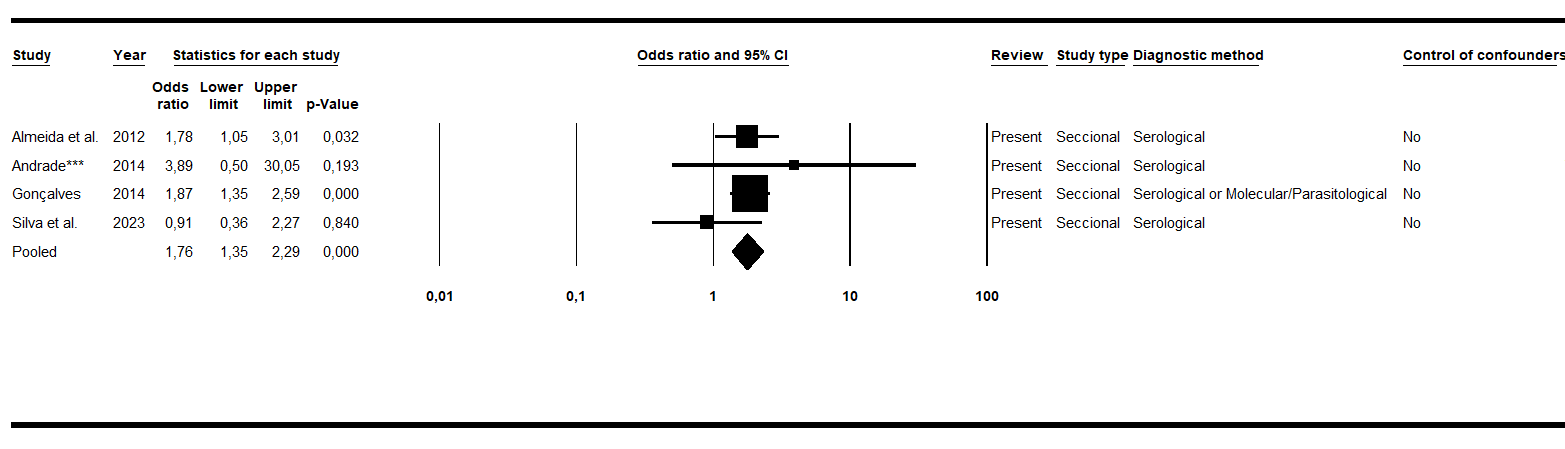


**Fig. S4.** Forest plot for the watchdog (guard dog) variable. Superscripts: * result of a serological test in a study involving two diagnostic tests; ** second different serological test result; *** third different serological test result; 1 different studies by the same author and year; 2 second result in a single publication; 3 third result in a single publication; i second result of the same study; ii third result of the same study. Squares represent the weight of each study, whereas diamonds represent the summary estimate of each subgroup. Reference: No, odds ratio = 1.


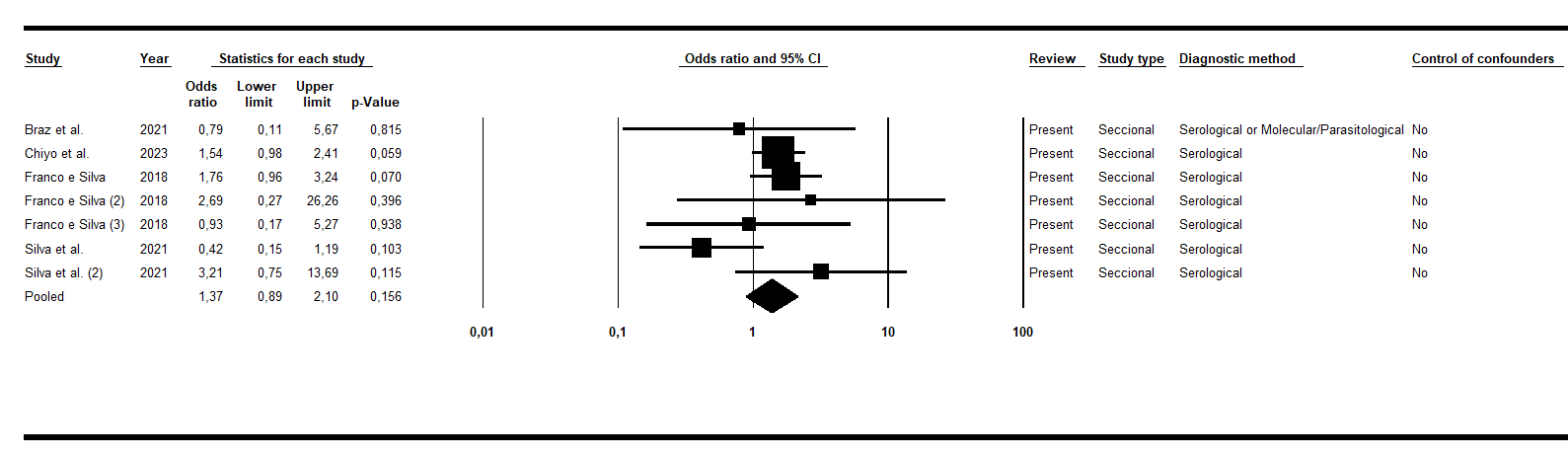


**Fig. S5.** Forest plot for the dog acquisition method variable. Superscripts: * result of a serological test in a study involving two diagnostic tests; ** second different serological test result; *** third different serological test result; 1 different studies by the same author and year; 2 second result in a single publication; 3 third result in a single publication; i second result of the same study; ii third result of the same study. Squares represent the weight of each study, whereas diamonds represent the summary estimate of each subgroup. Reference: Other (in relation to adopted from the streets), odds ratio = 1.


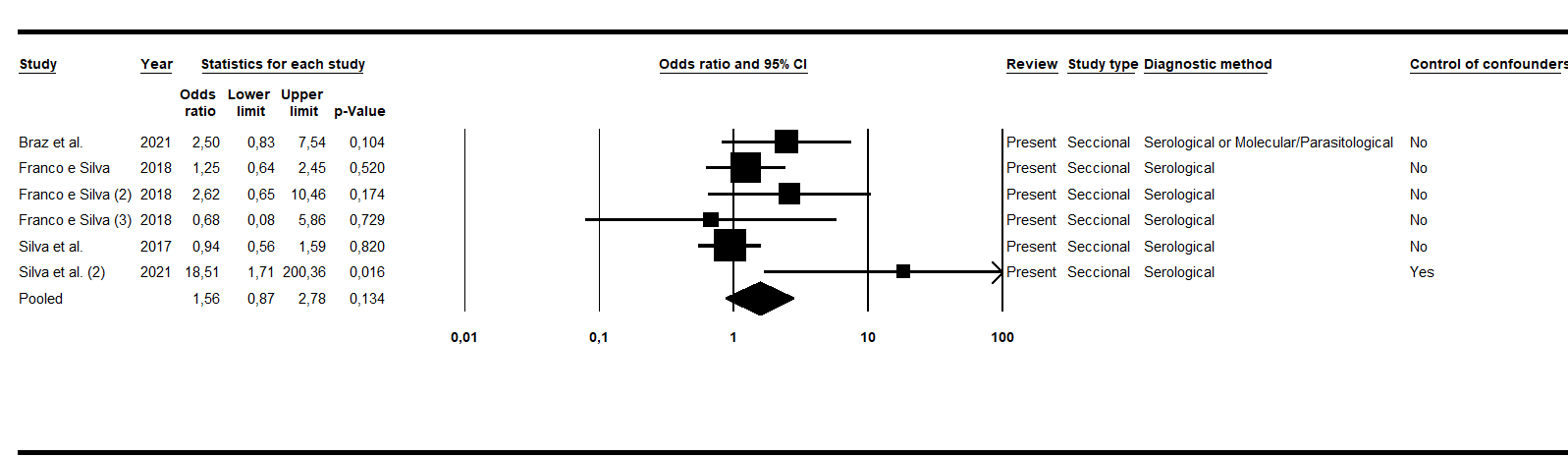


**Fig. S6.** Forest plot for the hunting dog variable. Superscripts: * result of a serological test in a study involving two diagnostic tests; ** second different serological test result; *** third different serological test result; 1 different studies by the same author and year; 2 second result in a single publication; 3 third result in a single publication; i second result of the same study; ii third result of the same study. Squares represent the weight of each study, whereas diamonds represent the summary estimate of each subgroup. Reference: No, odds ratio = 1.


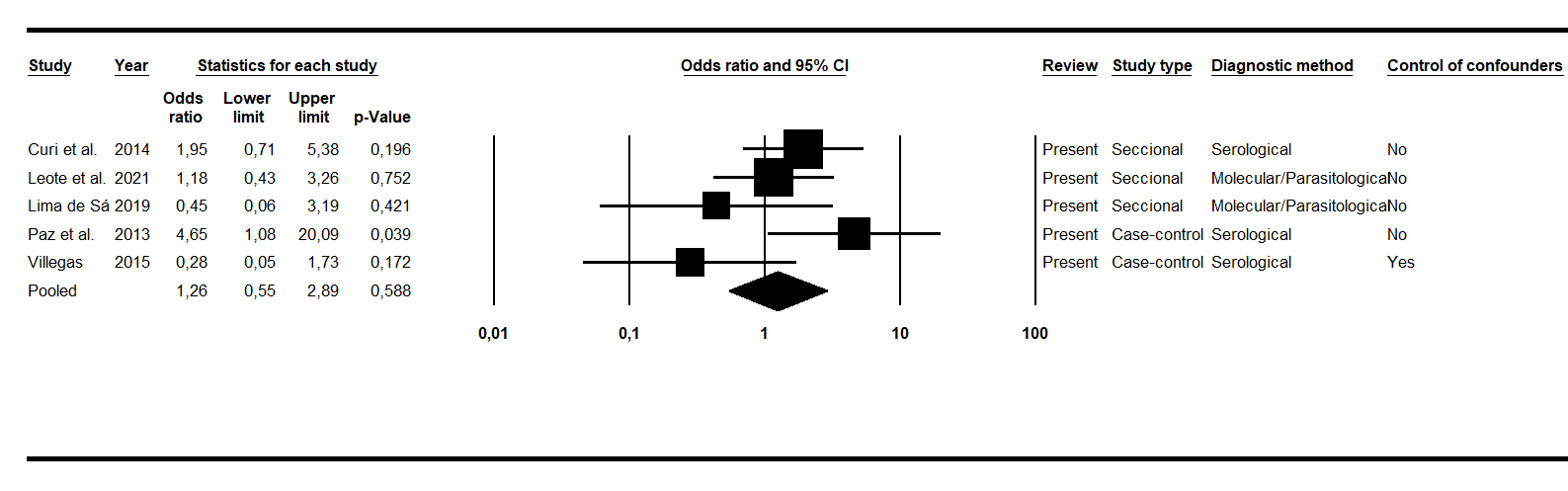


**Fig. S7.** Forest plot for the neutered dog variable. Superscripts: * result of a serological test in a study involving two diagnostic tests; ** second different serological test result; *** third different serological test result; 1 different studies by the same author and year; 2 second result in a single publication; 3 third result in a single publication; i second result of the same study; ii third result of the same study. Squares represent the weight of each study, whereas diamonds represent the summary estimate of each subgroup. Reference: No, odds ratio = 1.


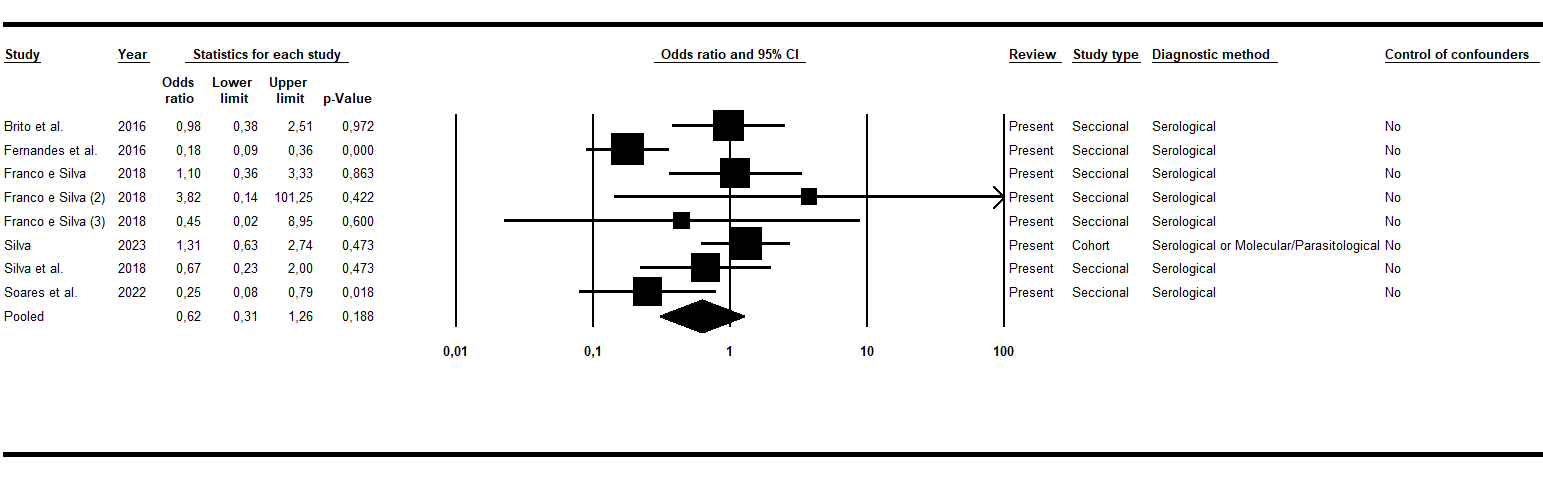


**Fig. S8.** Forest plot for the dog food variable. Superscripts: * result of a serological test in a study involving two diagnostic tests; ** second different serological test result; *** third different serological test result; 1 different studies by the same author and year; 2 second result in a single publication; 3 third result in a single publication; i second result of the same study; ii third result of the same study. Squares represent the weight of each study, whereas diamonds represent the summary estimate of each subgroup. Reference: Home-prepared food, odds ratio = 1.


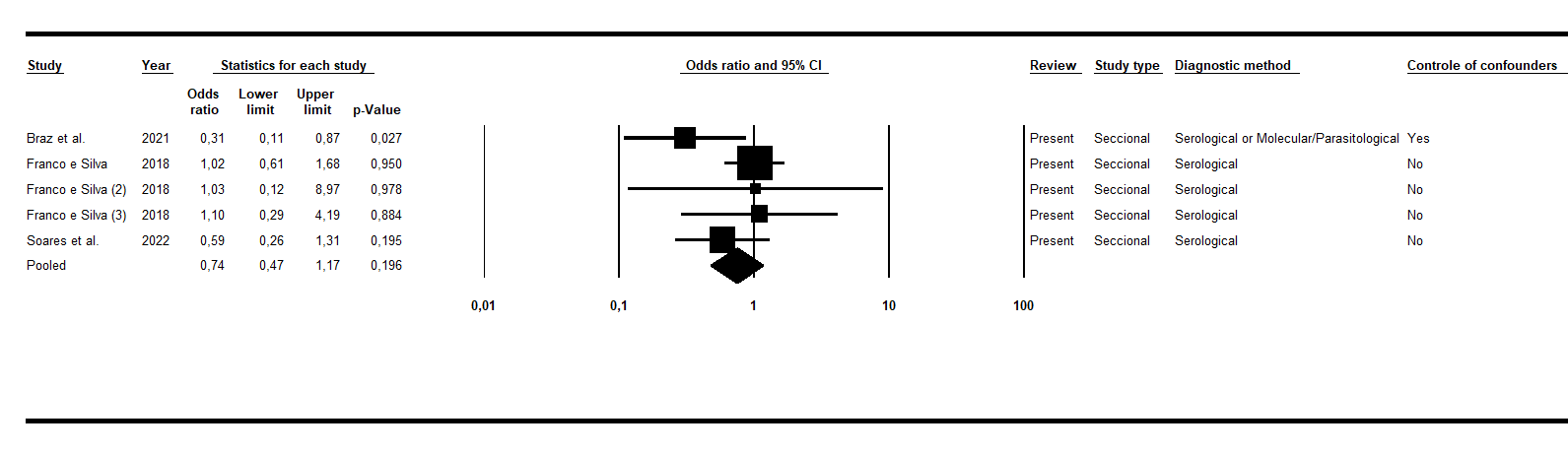


**Fig. S9.** Forest plot for the dewormed dog variable. Superscripts: * result of a serological test in a study involving two diagnostic tests; ** second different serological test result; *** third different serological test result; 1 different studies by the same author and year; 2 second result in a single publication; 3 third result in a single publication; i second result of the same study; ii third result of the same study. Squares represent the weight of each study, whereas diamonds represent the summary estimate of each subgroup. Reference: No, odds ratio = 1.


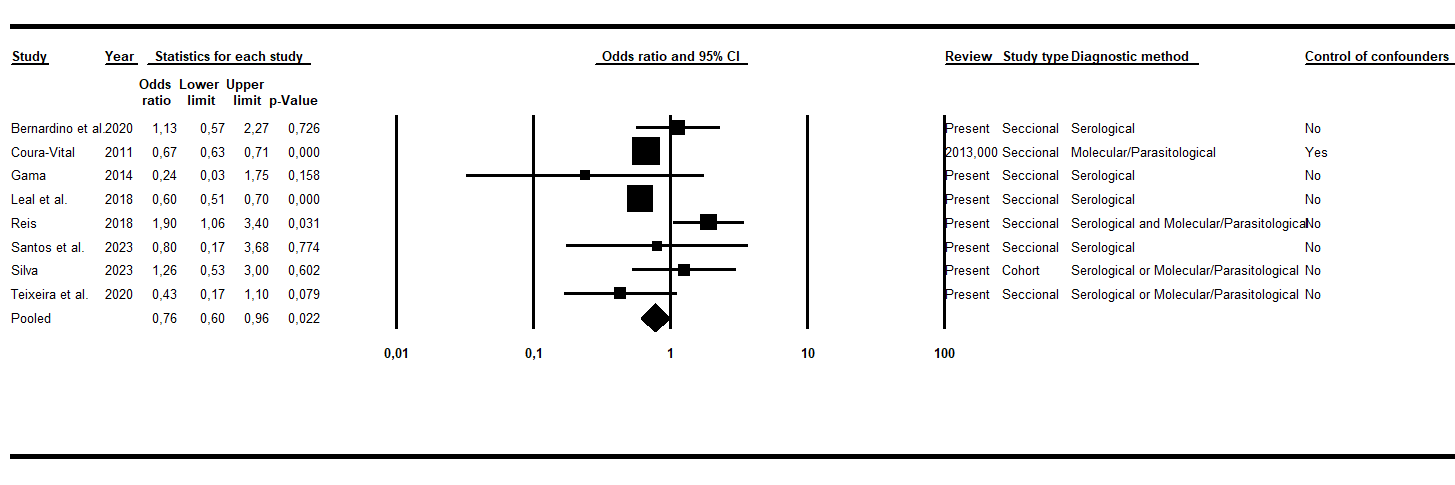


**Fig. S10.** Forest plot for the regular veterinary evaluation variable. Superscripts: * result of a serological test in a study involving two diagnostic tests; ** second different serological test result; *** third different serological test result; 1 different studies by the same author and year; 2 second result in a single publication; 3 third result in a single publication; i second result of the same study; ii third result of the same study. Squares represent the weight of each study, whereas diamonds represent the summary estimate of each subgroup. Reference: No, odds ratio = 1.


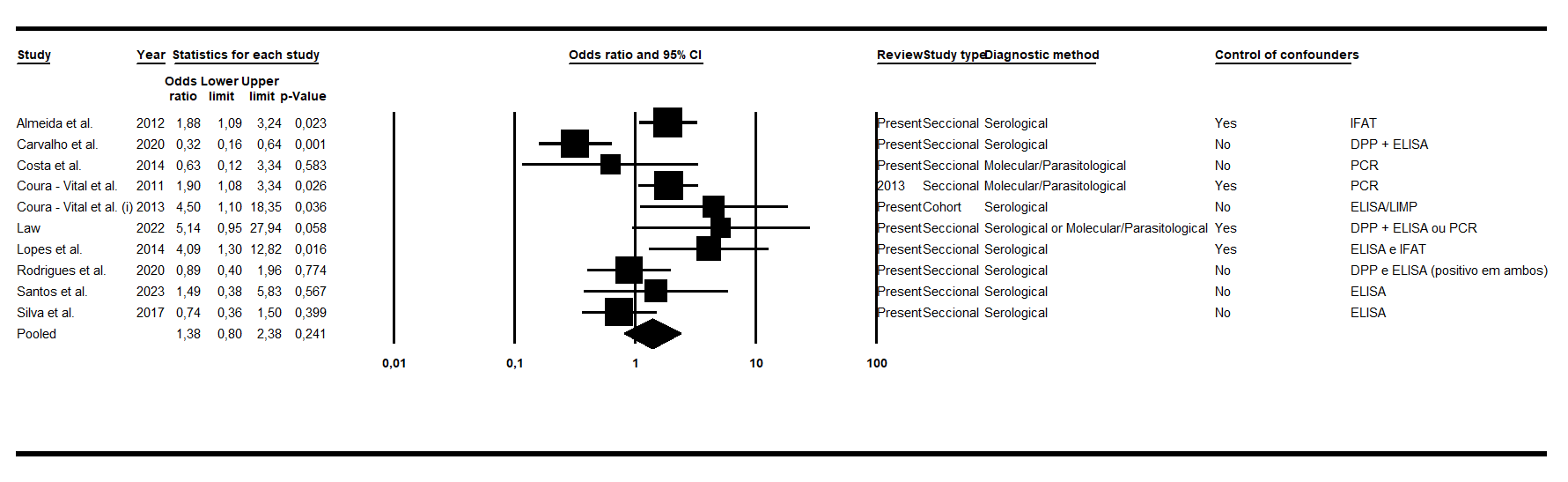


**Fig. S11.** Forest plot for the understanding of the disease variable. Superscripts: * result of a serological test in a study involving two diagnostic tests; ** second different serological test result; *** third different serological test result; 1 different studies by the same author and year; 2 second result in a single publication; 3 third result in a single publication; i second result of the same study; ii third result of the same study. Squares represent the weight of each study, whereas diamonds represent the summary estimate of each subgroup. Reference: No, odds ratio = 1.


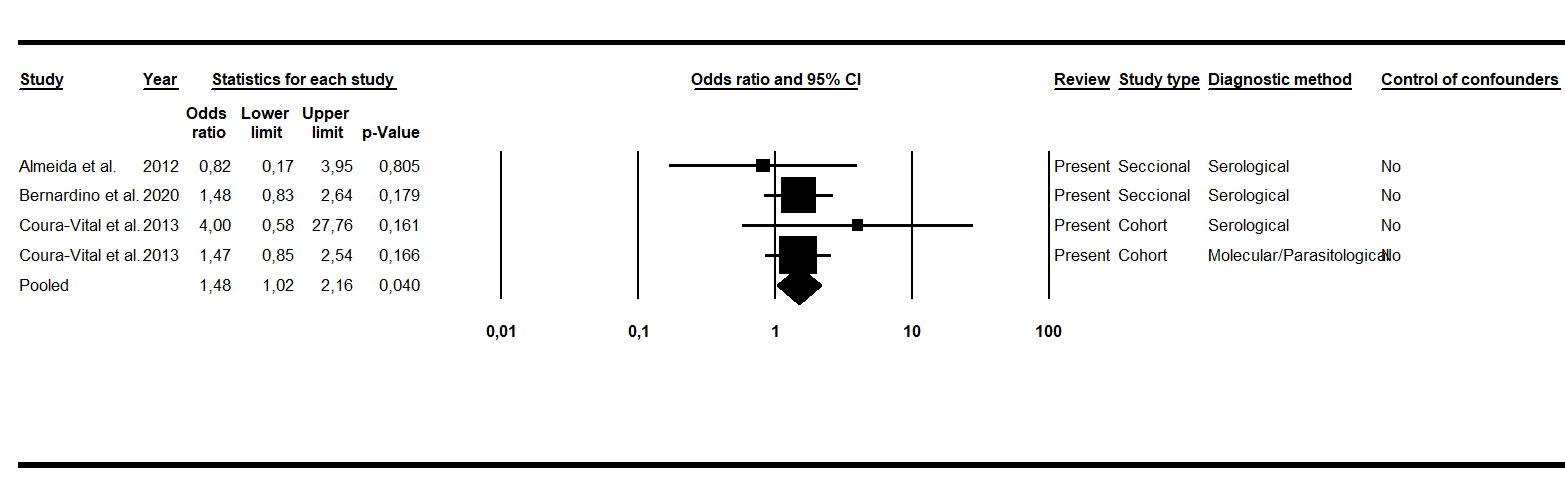


**Fig. S12.** Forest plot for the presence of vectors inside the house variable. Superscripts: * result of a serological test in a study involving two diagnostic tests; ** second different serological test result; *** third different serological test result; 1 different studies by the same author and year; 2 second result in a single publication; 3 third result in a single publication; i second result of the same study; ii third result of the same study. Squares represent the weight of each study, whereas diamonds represent the summary estimate of each subgroup. Reference: No, odds ratio = 1.


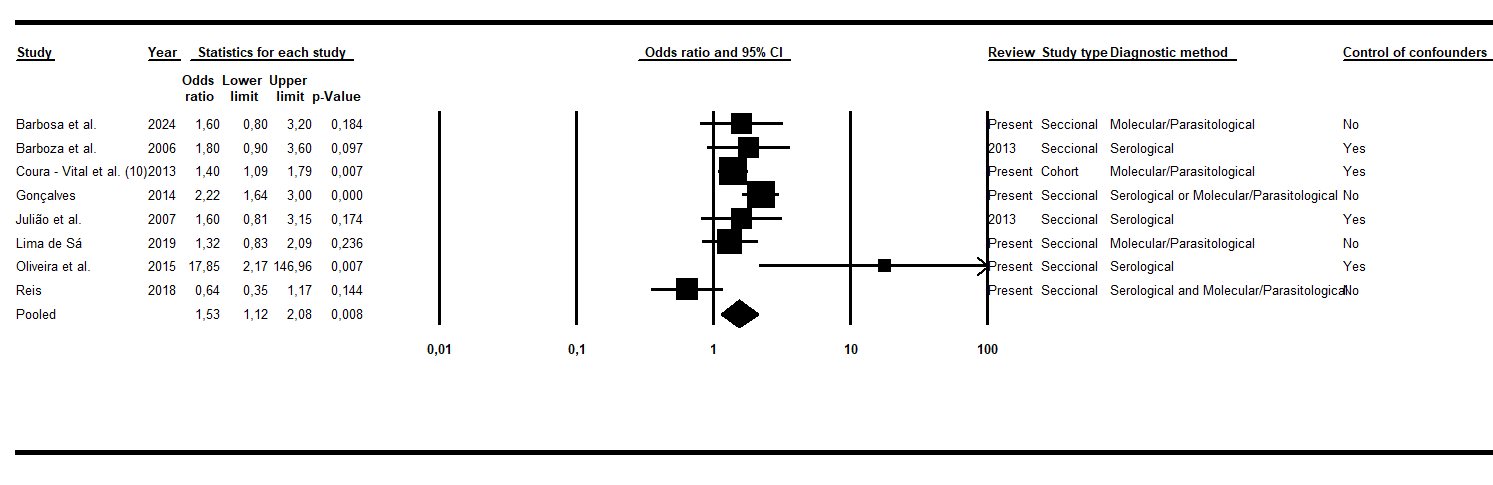


**Fig. S13.** Forest plot for the previous case of CVL in the household variable. Superscripts: * result of a serological test in a study involving two diagnostic tests; ** second different serological test result; *** third different serological test result; 1 different studies by the same author and year; 2 second result in a single publication; 3 third result in a single publication; i second result of the same study; ii third result of the same study. Squares represent the weight of each study, whereas diamonds represent the summary estimate of each subgroup. Reference: No, odds ratio = 1.

**
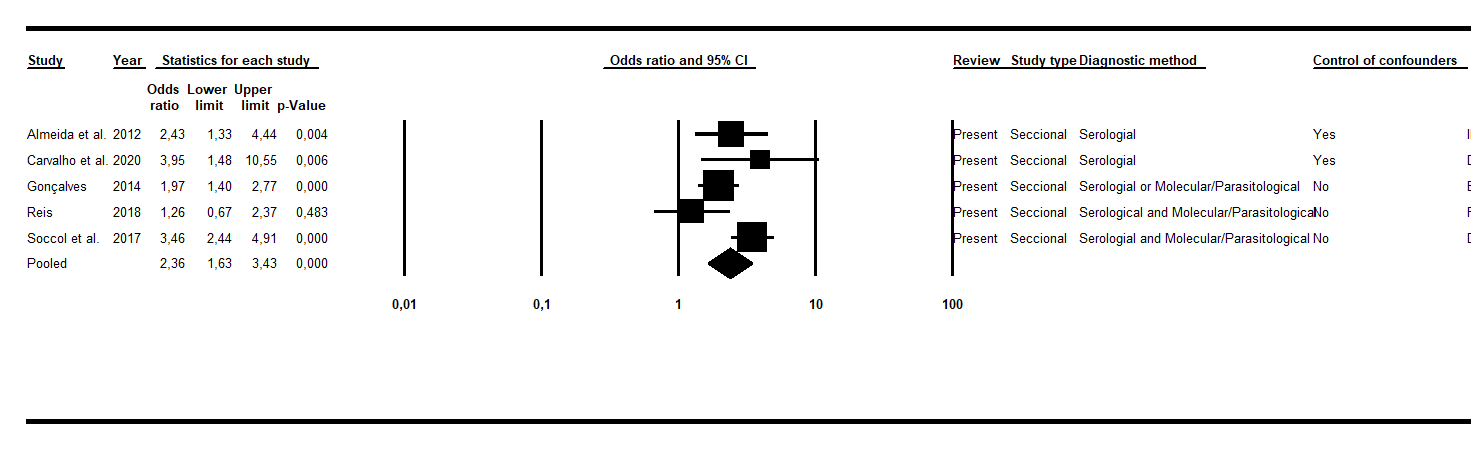
**

**Fig. S14.** Forest plot for the presence of neighbors with CVL variable. Superscripts: * result of a serological test in a study involving two diagnostic tests; ** second different serological test result; *** third different serological test result; 1 different studies by the same author and year; 2 second result in a single publication; 3 third result in a single publication; i second result of the same study; ii third result of the same study. Squares represent the weight of each study, whereas diamonds represent the summary estimate of each subgroup. Reference: No, odds ratio = 1.


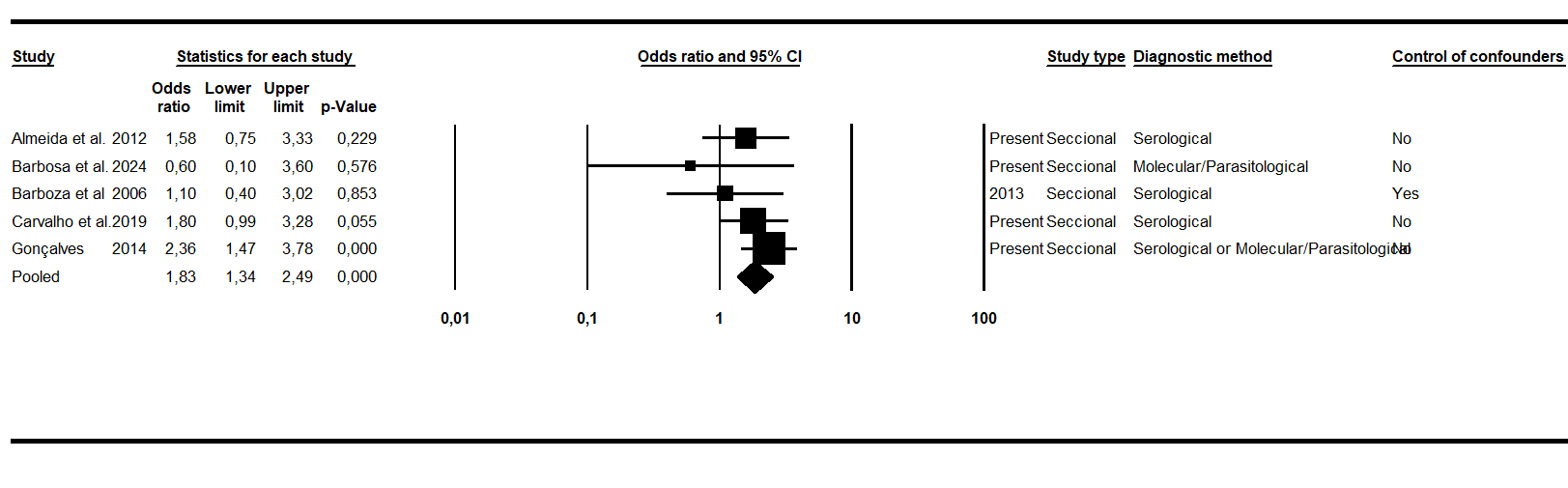


**Fig. S15.** Forest plot for the previous occurrence of human visceral leishmaniasis variable. Superscripts: * result of a serological test in a study involving two diagnostic tests; ** second different serological test result; *** third different serological test result; 1 different studies by the same author and year; 2 second result in a single publication; 3 third result in a single publication; i second result of the same study; ii third result of the same study. Squares represent the weight of each study, whereas diamonds represent the summary estimate of each subgroup. Reference: No, odds ratio = 1.
